# Synthesis and Biological Activity Evaluation of Novel Heterocyclic Pleuromutilin Derivatives

**DOI:** 10.3390/molecules22060996

**Published:** 2017-06-15

**Authors:** Yunpeng Yi, Yunxing Fu, Pengcheng Dong, Wenwen Qin, Yu Liu, Jiangping Liang, Ruofeng Shang

**Affiliations:** Key Laboratory of New Animal Drug Project of Gansu Province, Key Laboratory of Veterinary Pharmaceutical Development, Ministry of Agriculture, Lanzhou Institute of Husbandry and Pharmaceutical Sciences of CAAS, Lanzhou 730050, China; yiyp@foxmail.com (Y.Y.); 1394305674@163.com (Y.F.); dpch258@sina.com (P.D.); qinwenwen1103@163.com (W.Q.); yangguang8684@163.com (Y.L.); liangjp100@sina.com (J.L.)

**Keywords:** pleuromutilin derivatives, antibacterial activity, synthesis, molecular docking

## Abstract

A series of pleuromutilin derivatives were synthesized by two synthetic procedures under mild reaction conditions and characterized by Nuclear Magnetic Resonance (NMR), Infrared Spectroscopy (IR), and High Resolution Mass Spectrometer (HRMS). Most of the derivatives with heterocyclic groups at the C-14 side of pleuromutilin exhibited excellent in vitro antibacterial activities against *Staphylococcus aureus*, methicillin-resistant *Staphylococcus aureus* (MRSA), methicillin-resistant *Staphylococcus epidermidis* (MRSE), and vancomycin-resistant *Enterococcus* (VRE) in vitro antibacterial activity. The synthesized derivatives which contained pyrimidine rings, **3a**, **3b**, and **3f**, displayed modest antibacterial activities. Compound **3a**, the most active antibacterial agent, displayed rapid bactericidal activity and affected bacterial growth in the same manner as that of tiamulin fumarate. Moreover, molecular docking studies of **3a** and lefamulin provided similar information about the interactions between the compounds and 50S ribosomal subunit. The results of the study show that pyrimidine rings should be considered in the drug design of pleuromutilin derivatives.

## 1. Introduction

Antibiotics have been necessary life-saving drugs since the advent of penicillin in 1928 [1]. However, antibiotic-treatment faces total defeat as a result from drug resistance. Meanwhile, the discovery of antibiotics has gotten stuck in bottleneck. Although there have been more than 31 lead classes of antibiotics found and 160 kinds of antibiotics approved from the 1890s–1980s, few lead class antibiotics have been found in the past thirty years [1].

Many antibiotic treatment failures were associated with the spread of bacterial drug resistance, such as methicillin-resistant *Staphylococcus aureus* (MRSA), methicillin-resistant *Staphylococcus epidermidis* (MRSE), and vancomycin-resistant *Enterococcus* (VRE) [2]. Consequently, the study for clinically-available potent antibiotics that are effective against multidrug-resistant pathogens is becoming exceedingly urgent [3].

**Figure 1:**
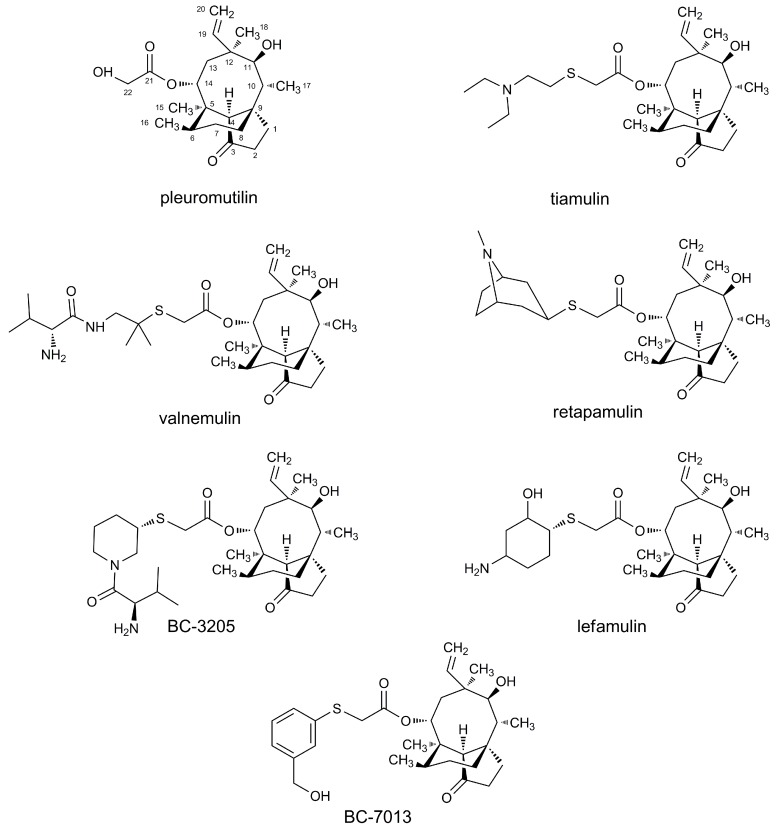
Structural formulas of pleuromutilin and derivation thereof.

Pleuromutilin was first discovered and isolated from basidiomycetes, Pleurotus mutilus, and P. passeckerianus in 1951 [4]. Tiamulin (Figure 1) was the first derivative approved for veterinary use in 1979, following which valnemulin (Figure 1) was approved in 1999. It was not until 2007 that retapamulin (Figure 1) was used in human medicine [4]. The C-14 side of pleuromutilin is the modest modification position for designing a high antibacterial agent and thus improves biological activity and enhances water-solubility [5,6,7,8].

The antibacterial mechanisms of pleuromutilin derivatives are to inhibit protein synthesis by blocking the peptidyl transferase center (PTC). Their tricyclic mutilin core of pleuromutlin derivative intervenes the A-site and the C-14 side chain extends to the P-site, which disturbs the translation of mRNA [4,9,10,11,12].

Many authors realized that thioether’s presence in C-14 could improve its antibacterial activities [11,12]. Recently, three new potential drugs, BC-3205, BC-7013, and lefamulin, which contain heterocyclic groups, have been approved in clinical trials. Nabriva’s lead product, lefamulin, has entered phase III of clinical trials on community-acquired bacterial pneumonia [12] and has received U.S. Food and Drug Administrationfast-track status [13].

In the present project, we decided to screen potential derivatives on the pleuromutilin skeleton, as well as to increase our knowledge of the binding site by molecular docking.

## 2. Results and Discussion

### 2.1. Chemistry

**Scheme 1:**
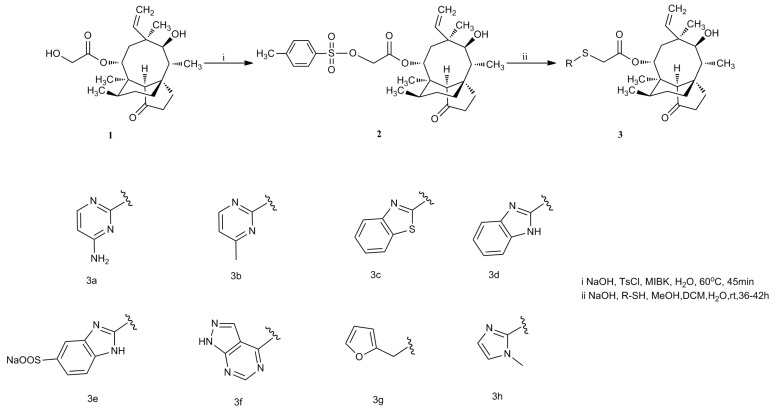
Synthesis of compounds **3a**–**3h**.

**Scheme 2:**
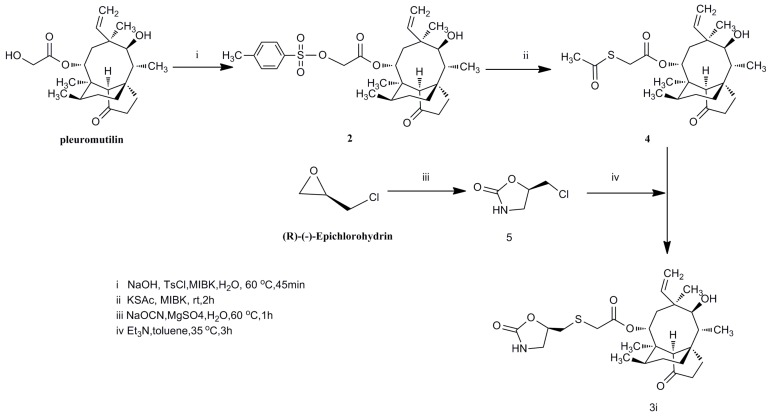
Synthesis of compound **3i**.

Mutilin 14-tosyloxyacrtate (**2**) was prepared by pleuromutilin and p-toluene sulfonyl chloride under basic conditions in 78% yield. The pleuromutilin derivatives **3a**–**h** were formed, as shown in Scheme 1. Mercaptan, used as an nucleophilic agent, easily attacked the C21-position of 14-tosyloxyacrtate under basic conditions. The synthetic route for compound **3i** is depicted in Scheme 2. Compound **4** was prepared by reacting mutilin 14-tosyloxyacrtate (2) with sulfocarbamide under alkaline conditions. Compound **3i** was obtained directly from compound **4** and **5** with a one pot reaction.

**Figure 2:**
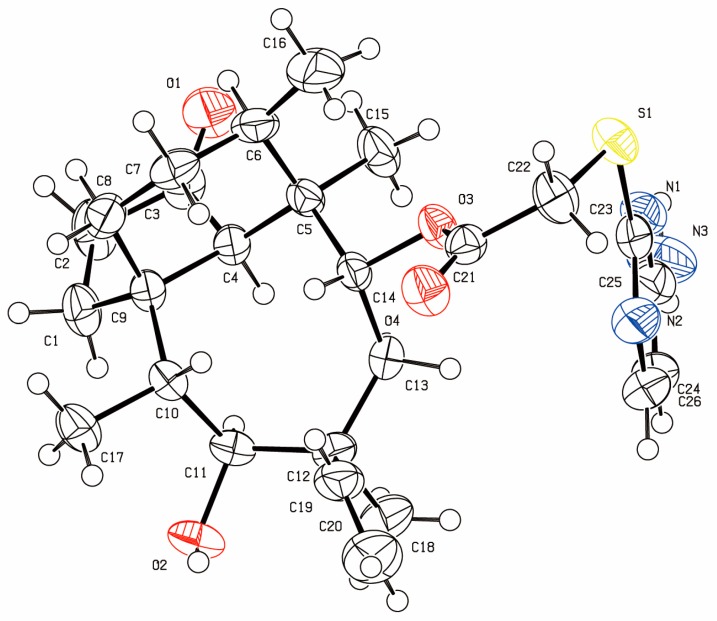
Oak Ridge Thermal-Ellipsoid Plot Program (ORTEP) diagram for compound **3a** with ellipsoids set at 75% probability.

All synthesis compounds were characterized by Nuclear Magnetic Resonance (NMR), Infrared Spectroscopy (IR) (Thermo Nicolet Corporation, Waltham, MA, USA.), and High Resolution Mass Spectrometer (HRMS) (Bruker Corporation, Billerica, MA, U.SA). A colorless crystal of compound **3a**, block-like, was obtained by slow evaporation of a chloroform solution (Figure 2).

### 2.2. Antibacterial Activity

The synthesized derivatives **3a**–**i** were evaluated for their antibacterial activity against several representative Gram-positive strains, including *Staphylococcus aureus*, methicillin-resistant *Staphylococcus aureus* (MRSA), methicillin-resistant *Staphylococcus epidermidis* (MRSE), and vancomycin-resistant *Enterococcus* (VRE), and a Gram-negative bacterium, *E. coli*. Tiamulin fumarate served as a control. The results of minimum inhibitory concentrations (MICs) and lipophilicities (Clogp), which were predicted by ACD/Labs (Toronto, Ontario, ON, Canada), are shown in Table 1. The synthesized pleuromutilin derivatives exhibited modest to excellent antibacterial activity against *S. aureus*, MRSA, MRSE, and VRE (MIC, 0.0625–2 μg/mL), respectively, while they showed poor activity against *E. coli.* Compounds **3a**, **3b**, and **3f** with a pyrimidine group showed higher antimicrobial activity than the other derivatives.

**Figure 3:**
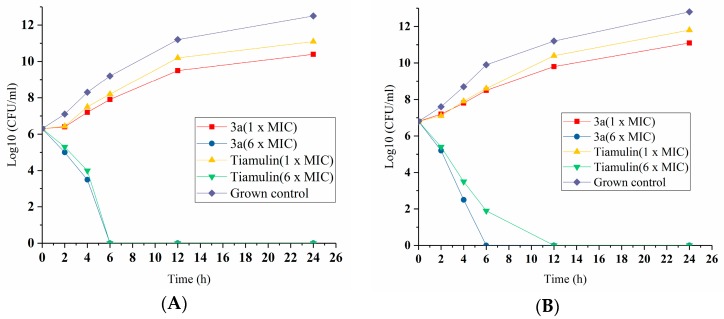
Time-kill kinetics of compound **3a** against *S. aureus* (ATCC 25923) (**A**) and MRSA (ATCC 43300) (**B**). Mean values of the CFU/mL (colony forming units per milliliter) were obtained from measurements taken in triplicate.

The most potent compound, **3a**, displayed promising antibacterial activity, the lowest MIC value in **3a**–**i** and was therefore further evaluated for in vitro time-kill assay [14]. The bactericidal properties of **3a** were compared at 1 × MIC and 6 × MIC against *S. aureus* (**3a** MIC = 0.0625 μg/mL, tiamulin MIC = 0.0625 μg/mL), and MRSA (**3a** MIC = 0.125 μg/mL, tiamulin MIC = 0.25 μg/mL) (Figure 2). As shown in Figure 3, **3a** displayed a concentration-dependent effect, with faster killing kinetics at higher concentrations. Although 1 × MIC of compound **6a** and tiamulin slowed bacterial propagation, their 6 × MIC achieved a 3-log_10_ reduction in 4–6 h. Compared to tiamulin, **3a** showed more rapid bactericidal kinetics against *S. aureus* and MRSA with the same concentration (1 × MIC). Notably, **3a** at 6 × MIC concentration reduced the viable count by approximately 6.8-log_10_ and achieved complete killing after 6 h of incubation. 

Further comparison of the physicochemical parameters of compounds **3a**–**i**, having a relatively hydrophilic C-14 side chain, shows that at good solubility of all of these derivatives. The lipophilicity of compound **3a** (ClogP = 3.23) is close to that of tiamulin, which contributes to its antibacterial activity comparable to that of tiamulin [15]. In summary, **3a** exhibited signigicantly improved bactericidal activity compared to that of tiamulin.

### 2.3. Molecular Docking Studies

**Figure 4:**
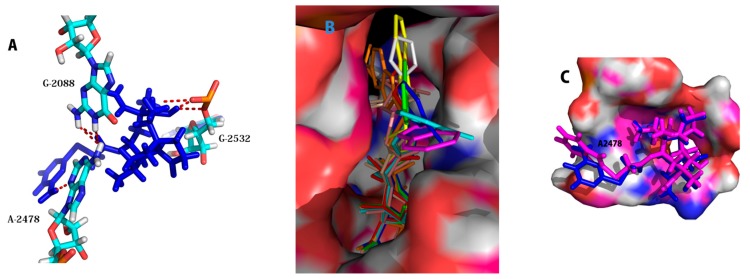
(**A**) Docking modes of the synthesized compounds into 5HL7. Key amino acid residues and ligand-active site interactions are shown. **3a** (blue) to 5HL7; Important residues are drawn in sticks and different color. Hydrogen bonds are showed as dashed red lines; (**B**) The best pose of the compounds obtained from the docking study in the active site of the peptidyl transferase center. (**3a** blue, **3b** cyan, **3c** yellow, **3d** magentas, **3e** oranges, **3f** wheat, **3g** gray, **3h** green, **3i** salmon, lefamulin red); (**C**) **3a** (blue) and **3d** (magentas) in 5HL7.

Molecular docking was performed with the aim of revealing the relations of the derivatives with their antibacterial activity at the atomic level. The suggestion that all pleuromutilin derivatives have been demonstrated to be effective might owe to their binding of the bacterial ribosome to PTC. To understand this phenomenon, we performed molecular docking to determine the reliability of this proposal. On applying Homdock software [16], the redocking of lefamulin into 5Hl7 [12] placed the compound in the PTC as that X-ray crystallography structure. The crystal structures was a typical complex of the 50 s ribosome about *S. aureus* and lefamulin. The docking results showed that the eight-membered ring can bind to the active site of the ribosome in the same manner as lefamulin with RMSD (Root-mean-square Deviation) at 0.88–1.12 Å (Figure 4, Table 2). Hydroxyl groups in all of the derivatives were located in a suitable position to form hydrogen bonds with G-2532, and some ester carbonyl groups were bound to C-2088 as hydrogen bonds. Although the tricyclic mutilin core did not form any hydrogen bonds with the PTC, it is stabilized by hydrophobic and Van der Waals interactions. Compounds **3b** and **3d** formed three hydrogen bonds with similar docking modes. Compound **3a**, the most promising of the candidates (ΔGb = −8.855 kcal/mol), bonded to PTC as a typical characteristic based on the docking results. Interestingly, **3a**, **3b**, and **3f** exhibited generally low energy and MIC values. The results were probably caused by its amine in pyrimidine, which plays an important role in the binding of the complex (Figure 4B).

Despite their similarities in H-binding, the activity of **3a** was better than **3d**. When we modeled the structural overlaps of compounds **3a** and **3d**, their side chain appeared markedly different. The pocket of PTC showed hydrophilicity (blue in Figure 4C) at the A-2478 position, which could make **3a** more stable than **3d**.

The binding sites of the pleuromutilin derivatives were different from that of other antibiotics, like macrocyclic and clindamycin. The macrocyclic ring was bound at aminoacyl-tRNAs at the bacterial ribosome (A-site) [17], which is the same side of the ribosomal tunnel as its side chain. In the crystal structure (PDB ID:1JZX), clindamycin also binds to the A-site of their PTC of bacterial ribosomes [18].

## 3. Experimental Section

### 3.1. Synthesis

#### General

All chemical reagents were purchased from *J* and K Chemical or Sigma-Aldrich Chemistry Co. (Darmstadt, Germany). Unless otherwise noted, all reactions were conducted under atmosphere. Thin-layer chromatography (TLC) analysis (Qingdao Haiyang Chemical Co., Ltd., Shandong, Qingdao, China) was used to monitor the reaction process. Column chromatography was carried out on silica gel (200–300 mesh). The products were eluted in an appropriate solvent mixture under air pressure. Concentration and evaporation of the solvent after reaction or extraction was carried out on a rotary evaporator. IR spectra were obtained on a Thermo Nicolet NEXUS-670 spectrometer (Thermo Nicolet Corporationcompany, Waltham, MA, USA) and recorded as KBr thin films and absorptions are reported in cm^−1^. HRMS were obtained with a Bruker Daltonics APEX II 47e mass spectrometer. NMR spectra (Bruker Corporation, Billerica, MA, USA) were recorded on a Bruker-400 MHz spectrometer (Bruker Corporation, Billerica, MA, USA) in appropriate solvents. Chemical shifts (δ) were expressed in parts per million (ppm) relative to the tetramethylsilane. Multiplicities of NMR signals are designated as s (singlet), d (doublet), t (triplet), q (quartet), m (multiplet), br (broad), etc. ^13^C-NMR spectra were recorded on 100 MHz spectrometers. The single-crystal structure of the title compound was determined on a Bruker SMART APEX II X-diffractometer (Bruker Corporation, Billerica, MA, USA). All NMR, IR and HRMS datum were been added in Supplementary Materials.

*14-O-(p-Toluene sulfonyloxyacetyl)mutilin* (**2**). A 5 mL of NaOH aqueous solution (2 g, 50 mmol) was added dropwise to a mixture of pleuromutilin (7.57 g, 20 mmol) and *p*-toluenesulfonyl chloride (4.2 g, 22 mmol) in methyl isobutyl ketone (10 mL) and water (5 mL). The mixture was vigorously stirred for 45 min at 60 °C, then the reaction mixture was cooled to 10 °C and separated. The organic layer was washed with 5 mL water and 5 mL saturated sodium carbonate solution. The organic phase was dried overnight with anhydrous sodium sulfate. After filteration, the solvent was concentrated in vacuo to give 10.56 g of yellow oil. It was used in the next step without further purification. Yield: 93%. IR (KBr): 3446 (OH), 2924 (CH2), 2863 (CH2), 1732 (C=O), 1633 (C-C), 1597 (C=C), 1456 (C=C), 1371 (CH3), 1297 (C-O-C), 1233 (CH), 1117 (C-(C=O)-C), 1035 (C-O-C), 832 (CH), 664 (CH2=), 560 (CH2=) cm^−1^. ^1^H-NMR (400 MHz, CDCl3) δ 7.74 (d, *J* = 8.3 Hz, 2H), 7.26 (t, *J* = 13.3 Hz, 2H), 6.34 (dd, *J* = 17.4, 11.0 Hz, 1H), 5.70 (d, *J* = 8.5 Hz, 1H), 5.19 (dd, *J* = 55.1, 14.2 Hz, 2H), 4.47–4.33 (m, 2H), 3.28 (s, 1H), 2.38 (s, 3H), 2.25–2.09 (m, 3H), 2.01 (s, 1H), 1.99–1.88 (m, 1H), 1.71–1.62 (m, 1H), 1.61–1.51 (m, 2H), 1.46–1.38 (m, 2H), 1.35 (d, *J* = 7.8 Hz, 3H), 1.27 (d, *J* = 11.5 Hz, 1H), 1.18 (dd, *J* = 11.6, 4.5 Hz, 2H), 1.12–1.00 (m, 4H), 0.80 (d, *J* = 7.0 Hz, 3H), 0.55 (d, *J* = 7.0 Hz, 3H). ^13^C-NMR(100 MHz, CDCl3) δ215.71 (C=O), 163.87 (C=O), 144.29 (benzene-C), 137.70 (CH=), 131.63 (benzene-C), 128.91 (benzene-C), 127.09 (benzene-C), 116.38 (CH2=), 73.54 (CH), 69.29 (CH), 64.03 (CH), 57.02 (CH), 44.39 (C), 43.51 (CH2), 42.97 (C), 40.84 (C), 35.54 (CH), 35.03 (CH), 33.40 (CH2), 29.34 (CH3), 25.77(CH2), 25.39 (CH2), 23.81 (CH2), 20.68 (CH3), 15.53 (CH3), 13.76 (CH3), 10.47 (CH3). HRMS (ESI) calcd. [M + H]^+^ for C29H40O7S 533.250, found 533.2507.

*14-O-(Acetic acidthioacetyl)mutilin* (**4**). Compound **3** was prepared by stirring a mixing of compound **2** (10 mmol), potassium thioglycolate (20 mmol), and methyl isobutyl ketone (30 mL) in room temperature, and the mixture was stirred for 2 h. The mixture was extracted with water (10 mL). The organic phase were combined, dried over Na_2_SO_4_, and concentrated to give compound **3**. Yield: 80%. IR (KBr): 3448 (OH), 2967 (CH2), 2924 (CH3), 2865 (CH2), 1731 (C=O), 1702 (C=O), 1453 (C-C), 1419, 1384 (CH3), 1295 (C-O-C), 1183 (CH), 1154 (C-O), 1115 (C-(C=O)-C), 1017 (C-O-C) cm^−1^. ^1^H-NMR (400 MHz, CDCl3) δ 6.58–6.30 (m, 1H), 5.72 (d, *J* = 8.4 Hz, 1H), 5.43–5.14 (m, 2H), 3.63 (s, 2H), 3.36 (d, *J* = 6.4 Hz, 1H), 2.36 (dd, *J* = 13.5, 3.5 Hz, 3H), 2.35–2.27 (m, 1H), 2.21 (dd, *J* = 17.4, 7.9 Hz, 1H), 2.13 (d, *J* = 14.9 Hz, 1H), 2.04 (dd, *J* = 26.1, 17.5 Hz, 1H), 1.73 (dd, *J* = 31.8, 10.6 Hz, 2H), 1.68–1.62 (m, 2H), 1.62–1.41 (m, 6H), 1.41–1.24 (m, 2H), 1.24–0.99 (m, 4H), 0.89 (t, *J* = 8.4 Hz, 3H), 0.78–0.61 (m, 3H). ^13^C-NMR (101 MHz, CDCl3) δ 216.90 (C=O), 193.45 (C=O), 167.32 (C=O), 138.92 (CH=), 117.19 (CH2=), 74.60 (CH), 70.09 (CH), 58.13 (CH), 45.45 (C), 44.71 (CH2), 44.01 (C), 41.89 (C), 36.74 (CH), 36.00 (CH3), 34.45 (CH2), 32.20 (CH2), 30.42 (CH2), 30.06 (CH2), 26.84 (CH2), 26.41 (CH3), 24.82 (CH2), 16.74 (CH3), 14.81(CH3), 11.43 (CH3). HRMS (ESI) calcd. [M + H]^+^ for C24H36O5S 437.2356, found 437.2339.

*(R)-5-ChloroMethyl-2-oxazolidinone* (**5**). The title compound was prepared by stirring a mixing of magnesium sulphate (10 mmol), sodium cyanate (10 mmol) and water (50 mL) in room temperature. (R)-(−)-Epichlorohydrin to the solution (5 mmol) was added dropwise to the mixture. The result mixture was stirred under 60 °C for 1 h. The reaction mixture was concentrated in vacuo and extracted by ethyl acetate. The two layers were separated, and the organic layer was dried over Na_2_SO_4_, filtered, and concentrated to dryness. Yield: 57%. IR (KBr): 3365 (NH), 1744 (C=O), 1429 (C-N), 1240 (C-C(=O)-O), 736 (C-Cl) cm^−1^. ^1^H-NMR (400 MHz, DMSO) δ 7.60 (s, 1H), 4.84 (dd, *J* = 9.6, 4.7 Hz, 1H), 4.04–3.73 (m, 2H), 3.59 (t, *J* = 9.0 Hz, 1H), 3.25 (dd, *J* = 9.0, 6.2 Hz, 1H). ^13^C-NMR (101 MHz, d6-DMSO) δ 158.68 (C=O), 74.35 (CH), 46.66 (CH2), 43.03(CH2). HRMS (ESI) calcd. [M + H]^+^ for C4H6ClNO 136.0159, found 136.0150.

General Procedure for Synthesis of Compounds **3a**–**3g**. A mixing of thiols (1 mmol), sodium hydroxide (1.1 mmol), water (0.5 mL) and methanol (3 mL) were stirred in room temperature. After 30 min, compound **2** (1.1 mmol) in 5 mL CH_2_Cl_2_ was added dropwise to the mixture for 36 h–42 h. The mixture was concentrated in vacuo. The residue was dissolved by CH_2_Cl_2_. The solution was extracted three times with water. The organic phase was dried overnight with anhydrous sodium sulfate. The solvent was concentrated in vacuo to give crude products. The crude product was purified by silica gel column chromatography. 

*14-O-[(4-Amino-pyrimidinone-2-yl)thioacetyl]mutilin* (**3a**). Compound **3a** was prepared according to the general procedure from 14-*O*-(*p*-toluene sulfonyloxyacetyl) mutilinmutilin (2) and 4-amino-2-Pyrimidinone. The crude product was purified over silica gel column chromatography to give 4.09 g. Yield: 84%. IR (KBr): 3448 (OH), 2933 (CH2), 1730 (C=O), 1629 (C-C), 1583 (C=N), 1543 (C=C), 1467 (C=C), 1372 (CH3), 1249(C-C(=O)-O), 1153(C-O), 1117 (C-(C=O)-C), 1018 (C-O-C) cm^−1^. ^1^H-NMR (400 MHz, CDCl3) δ 7.89 (d, *J* = 5.1 Hz, 1H), 6.42 (dd, *J* = 17.1, 11.2 Hz, 1H), 6.04 (d, *J* = 5.2 Hz, 1H), 5.68 (d, *J* = 7.9 Hz, 1H), 5.17 (dd, *J* = 51.9, 14.0 Hz, 2H), 4.94 (s, 2H), 3.72 (dd, *J* = 32.8, 16.1 Hz, 2H), 3.28 (s, 1H), 2.24 (d, *J* = 6.5 Hz, 1H), 2.14 (dd, *J* = 14.8, 9.1 Hz, 2H), 2.02 (s, 1H), 1.94 (dd, *J* = 15.6, 8.5 Hz, 1H), 1.69 (d, *J* = 13.7 Hz, 1H), 1.61–1.52 (m, 2H), 1.48 (d, *J* = 12.0 Hz, 2H), 1.37 (s, 4H), 1.32–1.22 (m, 2H), 1.07 (s, 4H), 0.79 (d, *J* = 6.2 Hz, 3H), 0.68 (d, *J* = 6.2 Hz, 3H). ^13^C-NMR (101 MHz, CDCl3) δ 216.11 (C=O), 168.72 (pyrimidine-C), 167.20 (C=O), 161.35 (pyrimidine-C), 154.88 (pyrimidine-C), 138.26 (CH=), 116.02 (CH2=), 100.18 (pyrimidine-C), 73.60 (CH), 68.51(CH), 57.36 (CH), 57.21(CH2), 44.46 (CH2), 43.47 (C), 42.93 (C), 40.87 (CH), 35.81 (CH), 35.02 (CH), 33.48 (CH2), 33.06 (CH2), 29.45(CH2), 25.89 (CH3), 23.84 (CH2), 15.74 (CH3), 13.92 (CH3), 10.44 (CH3). HRMS (ESI) calcd. [M + H]^+^ for C26H37N3O4S 488.2578, found 488.2570.

*14-O-[(4-Methylpyrimidine-2-yl)thioacetyl]mutilin* (**3b**). Compound **3b** was prepared according to the general procedure from 14-*O*-(*p*-toluene sulfonyloxyacetyl) mutilin(2) and 4-methy-2-pyrimidinone. The crude product was purified over silica gel column chromatography to give 3.55 g. Yield: 73%. IR (KBr): 3439 (OH), 2935 (CH2), 1733 (C=O), 1658 (C-C), 1580 (C=N), 1535 (C=C), 1458 (C=C), 1396 (CH3), 1285 (C-O-C), 1118 (C-(C=O)-C) cm^−1^. ^1^H-NMR (400 MHz, CDCl3) δ 6.37 (dt, *J* = 33.7, 16.8 Hz, 1H), 5.99 (s, 1H), 5.69 (d, *J* = 8.4 Hz, 1H), 5.19 (dd, *J* = 56.5, 14.2 Hz, 2H), 3.88–3.73 (m, 2H), 3.29 (d, *J* = 5.6 Hz, 1H), 2.27–2.15 (m, 2H), 2.11 (d, *J* = 13.1 Hz, 3H), 2.02 (d, *J* = 6.4 Hz, 1H), 1.96 (d, *J* = 10.9 Hz, 1H), 1.69 (d, *J* = 14.2 Hz, 1H), 1.58 (dd, *J* = 21.0, 10.8 Hz, 2H), 1.49 (dd, *J* = 26.7, 13.3 Hz, 2H), 1.43–1.26 (m, 6H), 1.20 (dd, *J* = 17.5, 11.2 Hz, 2H), 1.05 (d, *J* = 20.9 Hz, 4H), 0.80 (d, *J* = 6.9 Hz, 3H), 0.67 (d, *J* = 6.9 Hz, 3H). ^13^C-NMR (101 MHz, CDCl3) δ 215.94 (C=O), 165.80(C=O), 164.69 (pyrimidine-C), 164.11 (pyrimidine-C), 157.85 (pyrimidine-C), 137.90 (CH=), 116.27 (CH2=), 107.63 (pyrimidine-C), 73.55 (CH), 69.14 (CH), 57.08 (CH), 44.43 (C), 43.49 (CH2), 42.94 (C), 40.87 (C), 35.70 (CH), 35.00 (CH3), 33.44 (CH2), 32.23 (CH2), 29.39 (CH2), 25.84(CH3), 25.36(CH2), 23.82 (CH2), 23.09 (CH2), 15.84(CH3), 13.84 (CH3), 10.46 (CH3). HRMS (ESI) calcd. [M + H]^+^ for C27H38N2O4S 487.2625, found 487.2623. 

*14-O-[(Benzimidazole-2-yl)thioacetyl]mutilin* (**3c**). Compound **3c** was prepared according to the general procedure from 14-*O*-(*p*-toluene sulfonyloxyacetyl) mutilin (2) and 2-mercaptobenzothiazole. The crude product was purified over silica gel column chromatography to give 3.54 g. Yield: 67%. IR (KBr): 3442 (OH), 2929 (CH2), 1731 (C=O), 1458 (C=C), 1429 (C-C), 1274 (C-O-C), 1153 (C-O), 1117 (C-(C=O)-C) cm^−1^. ^1^H-NMR (400 MHz, CDCl3) δ 7.77 (dd, *J* = 19.9, 8.0 Hz, 2H), 7.39 (t, *J* = 7.7 Hz, 1H), 7.29 (t, *J* = 7.6 Hz, 1H), 6.42 (dd, *J* = 17.4, 11.0 Hz, 1H), 5.76 (d, *J* = 8.5 Hz, 1H), 5.19 (dd, *J* = 57.6, 14.2 Hz, 2H), 4.08 (dd, *J* = 41.4, 16.2 Hz, 2H), 3.31 (d, *J* = 6.4 Hz, 1H), 2.29 (dd, *J* = 14.1, 7.2 Hz, 1H), 2.20 (dd, *J* = 11.6, 6.3 Hz, 1H), 2.06 (s, 1H), 1.98 (dd, *J* = 16.0, 8.5 Hz, 1H), 1.82–1.64 (m, 2H), 1.64–1.51 (m, 2H), 1.50–1.33 (m, 6H), 1.24 (dd, *J* = 14.3, 7.4 Hz, 2H), 1.13–0.97 (m, 4H), 0.85 (d, *J* = 7.0 Hz, 3H), 0.77 (d, *J* = 6.9 Hz, 3H). ^13^C-NMR (101 MHz, CDCl3) δ 215.94 (C=O), 165.84 (C=O), 163.54 (benzothiazole-C), 151.74 (benzothiazole-C), 137.76 (CH=), 134.49(benzothiazole-C), 125.02 (benzothiazole-C), 123.46 (benzothiazole-C), 120.68 (benzothiazole-C), 120.05 (benzothiazole-C), 116.21 (CH2=), 73.57 (CH), 69.20 (CH), 57.10 (CH), 44.42 (C), 43.41 (CH2), 42.90 (C), 40.86 (C), 35.74 (CH), 34.98 (CH), 34.67 (CH2), 33.43(CH2), 29.40 (CH2), 25.84 (CH2), 25.28 (CH3), 23.81 (CH2), 15.80(CH3), 13.81 (CH3), 10.44 (CH3). HRMS (ESI) calcd. [M + H]^+^ for C29H37NO4S 528.2237, found 528.2234.

*14-O-[(Benzothiazole-2-yl)thioacetyl]mutilin* (**3d**). Compound **3d** was prepared according to the general procedure from 14-*O*-(*p*-toluene sulfonyloxyacetyl) mutilin (2) and 2-mercaptobenzimidazole. The crude product was purified over silica gel column chromatography to give 3.68 g. Yield 72%. IR (KBr): 3423 (OH), 2925 (CH2), 1726 (C=O), 1458 (C=C), 1439 (C-C), 1271 (C-O-C), 1152 (C-O), 1117 (C-(C=O)-C) cm^−1^. ^1^H-NMR (400 MHz, CDCl3) δ 7.50 (dd, *J* = 5.8, 3.1 Hz, 2H), 7.20 (dd, *J* = 6.0, 3.2 Hz, 2H), 6.41 (dd, *J* = 17.4, 11.0 Hz, 1H), 5.79 (d, *J* = 8.4 Hz, 1H), 5.18 (dd, *J* = 43.7, 14.2 Hz, 2H), 3.91 (s, 2H), 3.73 (q, *J* = 7.0 Hz, 1H), 3.35 (d, *J* = 6.4 Hz, 1H), 2.35–2.28 (m, 1H), 2.27–2.17 (m, 1H), 2.08 (s, 1H), 2.03 (dd, *J* = 16.1, 8.6 Hz, 1H), 1.82–1.65 (m, 2H), 1.64–1.52 (m, 2H), 1.49–1.32 (m, 6H), 1.24 (t, *J* = 7.0 Hz, 2H), 1.16–1.05 (m, 4H), 0.88 (d, *J* = 6.9 Hz, 3H), 0.72 (d, *J* = 7.0 Hz, 3H). ^13^C-NMR (101 MHz, CDCl3) δ 215.89 (C=O), 167.78 (C=O), 147.20 (benzimidazole-C), 137.75 (CH=), 121.68 (benzimidazole-C), 116.28 (CH2=), 73.61 (CH), 69.82 (CH), 57.43 (CH), 57.09 (benzimidazole-C), 44.42 (C), 43.52 (CH2), 42.97 (C), 40.84 (C), 35.68 (CH), 35.04 (CH2), 34.16 (CH2), 33.42, 29.38 (CH2), 25.84 (CH2), 25.44 (CH3), 23.83 (CH2), 17.42 (CH2), 15.80 (CH3), 13.80 (CH3), 10.50 (CH3). HRMS (ESI) calcd. [M + H]^+^ for C29H38N2O4S 511.2526, found 511.2531.

*14-O-[(5-Benzimidazolesulfonate-2-yl)thioacetyl]mutilin* (**3e**). Compound **3e** was prepared according to the general procedure from 14-*O*-(*p*-toluene sulfonyloxyacetyl) mutilin (2) and sodium 2-mercapto-5-benzimidazolesulfonate dihydrate. The crude product was purified over silica gel column chromatography to give 3.2 g. Yield: 54.2%. IR (KBr): 3448 (OH), 2940 (CH2), 1732 (C=O) 1298 (C-O-C), 1190 (S=O), 1178 (S=O) cm^−1^. ^1^H-NMR (400 MHz, DMSO) δ 7.80 (s, 1H), 7.69 (d, *J* = 8.5 Hz, 1H), 7.60 (d, *J* = 8.5 Hz, 1H), 6.04 (dd, *J* = 17.8, 11.2 Hz, 1H), 5.50 (d, *J* = 8.1 Hz, 1H), 4.96 (dd, *J* = 38.2, 14.5 Hz, 2H), 4.38 (d, *J* = 4.4 Hz, 1H), 3.36 (d, *J* = 5.5 Hz, 1H), 2.51 (s, 1H), 2.35 (s, 1H), 2.13 (dd, *J* = 21.0, 10.7 Hz, 1H), 2.08–1.96 (m, 2H), 1.90 (dd, *J* = 16.0, 8.1 Hz, 1H), 1.60 (s, 2H), 1.44 (d, *J* = 6.9 Hz, 1H), 1.33 (d, *J* = 7.6 Hz, 2H), 1.27–1.12 (m, 6H), 1.10–1.02 (m, 1H), 1.02–0.90 (m, 4H), 0.78 (d, *J* = 6.8 Hz, 3H), 0.58 (t, *J* = 9.3 Hz, 3H). ^13^C-NMR (101 MHz, DMSO) δ 217.48 (C=O), 166.34 (C=O), 150.52 (benzimidazole-C), 145.67 (benzimidazole-C), 141.16 (benzimidazole-C), 133.41 (CH=), 128.59 (benzimidazole-C), 125.97 (benzimidazole-C), 123.22 (benzimidazole-C), 115.68 (CH2=), 113.22 (CH2), 110.71 (benzimidazole-C), 72.92 (CH), 71.44 (CH), 57.42 (CH), 45.35 (C), 44.58 (CH2), 41.95 (C), 36.89 (CH), 36.64 (CH), 35.03 (CH2), 34.40 (CH2), 30.53 (CH2), 29.06 (CH2), 26.99 (CH2), 24.85 (CH2), 16.47 (CH3), 14.64 (CH3), 11.96 (CH3). HRMS (ESI) calcd. [M + H]^+^ for C29H38N2O7S2 591.2193, found 591.2192.

*14-O-[(Pyryrazolo[3,4d]pyrimidine-4-yl)thioacetyl]mutilin* (**3f**). Compound **3f** was prepared according to the general procedure from 14-*O*-(*p*-toluene sulfonyloxyacetyl) mutilin (2) and 4-mercaptopyrazolo[3,4-*d*]pyrimidine. The crude product was purified over silica gel column chromatography to give 4.30 g. Yield: 84%. IR (KBr): 3431 (OH), 2934 (CH2), 1732 (C=O), 1567 (C=C), 1456 (C=C), 1406 (C-C), 1271 (C-O-C), 1152 (C-O), 1117 (C-(C=O)-C), 981 (CH) cm-1. ^1^H-NMR (400 MHz, CDCl3) δ 8.65 (d, *J* = 37.7 Hz, 1H), 8.29–8.08 (m, 1H), 6.43 (dt, *J* = 21.8, 10.9 Hz, 1H), 5.80 (d, *J* = 8.4 Hz, 1H), 5.38–5.29 (m, 1H), 5.24 (dd, *J* = 33.0, 14.5 Hz, 2H), 4.18–4.02 (m, 2H), 3.38 (d, *J* = 6.3 Hz, 1H), 2.31 (dd, *J* = 13.9, 7.1 Hz, 1H), 2.22 (dd, *J* = 13.0, 7.5 Hz, 1H), 2.11 (t, *J* = 8.4 Hz, 1H), 2.04 (d, *J* = 7.9 Hz, 1H), 1.73 (dd, *J* = 31.9, 9.2 Hz, 2H), 1.65 (dd, *J* = 17.1, 7.1 Hz, 2H), 1.58–1.37 (m, 6H), 1.36–1.22 (m, 2H), 1.19–1.06 (m, 4H), 0.88 (t, *J* = 11.3 Hz, 3H), 0.79 (t, *J* = 12.6 Hz, 3H). ^13^C-NMR (101 MHz, CDCl3) δ 216.06 (C=O), 166.18 (C=O), 162.56 (pyrimidine-C), 153.05 (pyrimidine-C), 151.46 (pyrimidine-C), 137.88 (CH=), 131.82 (pyrazolo-C), 116.28 (CH2=), 110.65 (pyrimidine-C), 73.59 (CH), 69.22 (CH), 57.14 (CH), 44.46 (C), 43.57 (CH2), 42.92 (C), 40.88 (C), 35.75 (CH), 35.01 (CH2), 33.46 (CH2), 31.11 (CH2), 29.41 (CH2), 25.44 (CH3), 23.83 (CH2), 17.40(CH2), 15.74 (CH3), 13.86 (CH3), 10.47 (CH3). HRMS (ESI) calcd. [M + H]^+^ for C27H36N4O4S 513.2530, found 513.2527.

*14-O-[(Furfuryl-2-yl)thioacetyl]mutilin* (**3g**). Compound **3g** was prepared according to the general procedure from 14-*O*-(*p*-toluene sulfonyloxyacetyl) mutilin (2) and furfuryl mercaptan. The crude product was purified over silica gel column chromatography to give 3.53 g. Yield: 74%. IR (KBr): 3547 (OH), 2933 (CH2), 2882 (CH2), 1731 (C=O), 1455 (C=C), 1281 (C-O-C), 1150 (C-O), 1115 (C-(C=O)-C) cm^−1^. ^1^H-NMR (400 MHz, CDCl3) δ 7.29 (s, 1H), 6.42 (dd, *J* = 17.4, 11.0 Hz, 1H), 6.31–6.06 (m, 2H), 5.71 (d, *J* = 8.4 Hz, 1H), 5.23 (dd, *J* = 57.0, 14.2 Hz, 2H), 3.75 (s, 2H), 3.30 (s, 1H), 3.02 (s, 2H), 2.29 (dd, *J* = 13.7, 6.8 Hz, 1H), 2.15 (dt, *J* = 19.6, 8.8 Hz, 2H), 2.09–1.98 (m, 2H), 1.75–1.66 (m, 1H), 1.64–1.55 (m, 2H), 1.53–1.35 (m, 6H), 1.30 (t, *J* = 14.9 Hz, 2H), 1.17–1.05 (m, 4H), 0.82 (d, *J* = 7.0 Hz, 3H), 0.68 (d, *J* = 6.8 Hz, 3H). ^13^C-NMR (101 MHz, CDCl3) δ 215.99 (C=O), 167.67 (C=O), 149.27 (furan-C), 141.51(furan-C), 138.12 (CH=), 116.19 (CH2=), 109.37 (furan-C), 107.39 (furan-C), 73.65 (CH), 68.31 (CH), 57.21 (CH), 44.46 (C), 43.85 (CH2), 42.94 (C), 40.77 (C), 35.78 (CH), 35.04 (CH), 33.45 (CH2), 32.11 (CH2), 29.44 (CH2), 27.36 (CH2), 25.85 (CH3), 25.42 (CH2), 23.86 (CH2), 15.81 (CH3), 13.92 (CH3), 10.49 (CH3). HRMS (ESI) calcd. [M + H]^+^ for C27H38O5S 475.2513, found 475.2522.

*14-O-[(1-Methylimidazole-2-yl)thioacetyl]mutilin* (**3h**). Compound **3h** was prepared according to the general procedure from 14-*O*-(*p*-toluene sulfonyloxyacetyl) mutilin (2) and 2-mercapto-1-methylimidazole. The crude product was purified over silica gel column chromatography to give 3.46 g. Yield: 73%. IR (KBr): 3423 (OH), 2924 (CH2), 2863 (CH2), 1717 (C=O), 1455 (C=C), 1410 (C-C), 1280 (C-O-C), 1145 (C-O), 1117 (C-(C=O)-C) cm^−1^. ^1^H-NMR (400 MHz, CDCl3) δ 6.88 (d, *J* = 49.1 Hz, 2H), 6.35 (dd, *J* = 17.4, 11.0 Hz, 1H), 5.63 (d, *J* = 8.5 Hz, 1H), 5.32–5.01 (m, 2H), 3.92–3.62 (m, 2H), 3.56 (d, *J* = 11.0 Hz, 3H), 3.27 (s, 1H), 2.22 (dt, *J* = 13.8, 7.0 Hz, 1H), 2.19–2.07 (m, 2H), 2.03 (d, *J* = 21.6 Hz, 1H), 1.93 (dd, *J* = 16.0, 8.6 Hz, 1H), 1.77–1.61 (m, 1H), 1.58–1.46 (m, 3H), 1.45–1.35 (m, 2H), 1.31 (d, *J* = 14.3 Hz, 3H), 1.26–1.13 (m, 2H), 1.12–0.97 (m, 4H), 0.80 (d, *J* = 7.0 Hz, 3H), 0.59 (d, *J* = 6.9 Hz, 3H). ^13^C-NMR (101 MHz, CDCl3) δ 215.93 (C=O), 166.75 (C=O), 139.06 (imidazole-C), 138.01 (CH=), 128.55 (imidazole-C), 121.39 (imidazole-C), 116.07 (CH2=), 73.57 (CH), 68.77 (CH), 57.12 (CH), 44.42 (C), 43.48 (CH2), 42.94 (C), 40.77 (C), 36.08 (CH), 35.70 (CH2), 35.00 (CH3), 33.44 (CH2), 32.35 (CH2), 29.40 (CH2), 25.83 (CH2), 25.41(CH3), 23.82 (CH2), 15.61 (CH3), 13.79 (CH3), 10.44 (CH3). HRMS (ESI) calcd. [M + H]^+^ for C26H38N2O4S 475.2625, found 475.2630.

*14-O-(2-oxazolidinone,5-(methyl)-)(thioacetyl)mutilin* (**3i**)*.* A mixing of (*R*)-5-chloroMethyl-2-oxazolidinone (**5**) (1 mmol), sodiumiodide (0.1 mmol), and acetone (10 mL) were stirred in room temperature. After 30 min, the reaction solution was filtered and concentrated. Compound **4** (1.1 mmol) and triethylamine (20 mL) was added under N_2_. The solvent was stirred in 40 °C for 10 h. The mixture was extracted with water (10ml) and HCl (2N, 10 mL). The organic layers were concentrated in vacuo to give crude products. The crude product was purified by silica gel column chromatography. Yield: 62%. IR (KBr): 3422 (O), 2933 (CH2), 1735 (C=O), 1686 (C=O), 1458 (C=C), 1420 (C-C), 1284 (C-O-C), 1151 (C-O), 1117 (C-(C=O)-C) cm^−1^. ^1^H-NMR (400 MHz, CDCl3) δ 6.63–6.35 (m, 1H), 6.17 (d, *J* = 8.2 Hz, 1H), 5.75 (d, *J* = 8.2 Hz, 1H), 5.27 (dd, *J* = 51.9, 14.2 Hz, 2H), 4.92–4.67 (m, 1H), 3.82–3.62 (m, 1H), 3.45–3.32 (m, 2H), 3.30–3.07 (m, 2H), 2.99 (ddd, *J* = 9.9, 8.9, 4.4 Hz, 1H), 2.87 (dt, *J* = 12.8, 5.2 Hz, 1H), 2.34 (s, 1H), 2.28–2.17 (m, 2H), 2.11 (s, 1H), 2.08 (d, *J* = 8.6 Hz, 1H), 1.77 (d, *J* = 14.3 Hz, 1H), 1.65 (d, *J* = 10.4 Hz, 2H), 1.52 (dd, *J* = 25.3, 6.7 Hz, 2H), 1.44 (d, *J* = 1.0 Hz, 4H), 1.39 (s, 1H), 1.35–1.26 (m, 1H), 1.16 (d, *J* = 14.9 Hz, 4H), 0.89 (d, *J* = 6.8 Hz, 3H), 0.73 (d, *J* = 6.8 Hz, 3H). ^13^C-NMR (101 MHz, CDCl3) δ 216.99 (C=O), 168.63 (C=O), 159.41 (C=O), 139.16 (CH=), 117.14 (CH2=), 75.66 (CH), 74.61 (C), 69.70 (CH), 58.17 (CH), 45.46 (C), 45.06 (CH2), 44.88 (CH2), 43.95 (C), 41.78 (C), 36.75 (CH), 36.03 (CH), 35.99, 34.81 (CH), 34.45 (CH2), 30.42 (CH2), 26.86 (CH3), 26.41 (CH2), 24.84 (CH2), 16.82 (CH3), 14.88 (CH3), 11.48 (CH3). HRMS (ESI) calcd. [M + Na]^+^ for C26H39NO6S 516.2395, found 516.2394. 

### 3.2. Biological Evaluation 

#### 3.2.1. MIC Testing

The MIC values of **3a**–**i** and tiamulin fumarate against bacteria were determined using the broth dilution method [13]. Stock solutions of compounds were prepared in DMSO. The compounds were added to the test tube and serially diluted in Mueller-Hinton broth (the final concentration is 0.0625 μg/mL). Five bacteria, including *S. aureus*, MRSA, MRSE, VRE, and one Gram-negative bacterium, *E. coli*, were cultivated and added to the tube. The initial concentration of bacteria cannot be lower than 10^5^ CFU/mL. The broth was incubated at 36.7 °C for 18–24 h. MICs were read when the change of clarity in the broth was observed in the control test tube.

#### 3.2.2. Bactericidal time-kill kinetics

The two bacterias were prepared in Muellere Hinton broth at 37 °C for 6 h with shaking. The solution of compound **3a** and tiamulin fumarate, 1 × MIC and 6 × MIC, were added to the bacterial suspension so that the final concentrations were 10^6^~10^7^ CFU/mL, respectively. After specified time intervals (0, 1, 2, 4, 6, 12, and 24 h), 20 mL aliquots were serially diluted in 0.9% saline, plated on sterile Muellere Hinton agar plates, and incubated at 37 °C for 24 h. The viable colonies were counted and represented as log_10_ (CFU/mL). The same procedure was repeated in triplicate.

### 3.3. Molecular Modeling Studies

The 50 s ribosomal of *S. aureus* in complex with lefamulin (PDB ID: 5HL7) [12] was simulated useHomdock software in the Chil2 package (University of Pittsburgh, Pittsburgh, PA, U.S., version 0.99). The package contains a Graph-based molecular alignment (GMA) tool and a Monte-Carlo/Simulated Annealing (MC/SA) algorithm-based docking (GlamDock) (University of Pittsburghcompany, Pittsburgh, PA, USA, version 0.99) tool. Lefamulin was the template for flexible molecular alignment, and the interaction was optimized by GlamDock according to the ChillScore scoring function based on ChemScore with a smooth, improved potential. All the compounds were prepared with Avogadro software [19], including a 5000 steps Steepest Descent and 1000 steps Conjugate Gradients geometry optimization based on the MMFF94 force field. The docking site was set to base lefamulin. All compounds have compared to original conformation of 5HL7, which was kept for binding affinity comparison. Compounds and receptors were estimated by ChillScore. Hydrogen bonds and other interactions were detected and generated by PyMol 1.5.03 [20,21].

## 4. Conclusions

In summary, a series of novel pleuromutilin derivatives bearing heterocyclic ring at the C-14 side chain were synthesized. Our results show that a heterocyclic substituent bearing a amine group leads to excellent in vitro antibacterial activity against *S. aureus*, MRSA, MRSE, and VRE. Compounds that contain pyrimidine rings prepared in this thesis showed moderate to excellent biological activities, and the hydrophilicity of the side chain showed some correlations to its activity. Compound **3a**, the most effective compound, showed rapid bactericidal activity against *S. aureus* and MRSA in time-kill assay. Molecular docking studies also revealed that **3a** displayed lower Gibbs free energy. Thus, compound **3a** has been selected for further evaluation as a promising candidate for treating bacterial infection.

## Figures and Tables

**Table 1:** Antibacterial activity (Minimum Inhibitory Concentration) of the synthesized pleuromutilin derivatives.

Compound	MIC(μg/mL)					ClogP
	*E. coli* (ATCC25922)	MRSA (ATCC43300)	*S. aureus* (ATCC25923)	MRSE (ATCC51625)	VRE (ATCC51559)	
**3a**	4	0.125	0.0625	0.0625	0.0625	3.23
**3b**	4	0.5	0.0625	0.5	0.25	4.96
**3c**	8	0.25	0.5	0.5	1	4.99
**3d**	8	0.25	0.125	0.25	0.25	4.46
**3e**	16	1	0.5	1	2	2.49
**3f**	2	0.125	0.0625	0.25	2	2.95
**3g**	8	0.5	0.5	1	1	3.83
**3h**	16	0.5	1	1	0.125	2.89
**3i**	8	0.25	0.0625	0.25	0.5	1.85
Tiamulin	8	0.25	0.0625	0.5	0.5	3.63

**Table 2:** Binding RMSD (Root-mean-square Deviation), number of noncovalent molecular interactions and free energy.

Compound	RMSD ^a^	Residue	Atom of Compound	Hydro I Interaction	Distance (Å) ^b^	Angles (°)	△Gb (kcal/mol)
**3a**	0.98	G2532	OH (eight membered ring)	H-bonding	2.06	168.5	−8.855
		G2088	C=O ester	H-bonding	2.2	128.1	
		A2478	NH2	H-bonding	1.67	170.6	
**3b**	0.91	G2532	OH (eight membered ring)	H-bonding	2.08	175.1	−8.583
		G2088	C=O ester	H-bonding	2.21	132.6	
		C2090	NH	H-bonding	1.97	143.9	
**3c**	0.94	G2532	OH (eight membered ring)	H-bonding	1.67	175.8	−7.816
		G2088	C=O ester	H-bonding	2.22	133.1	
**3d**	0.92	G2532	OH (eight membered ring)	H-bonding	1.67	175.1	−8.065
		G2088	C=O ester	H-bonding	2.257	131.5	
		C2090	imidazole NH	H-bonding	1.87	147.5	
**3e**	1.12	G2532	OH (eight membered ring)	H-bondingπ-π	1.86	170.3	−7.523
		A2089	benzene ring	interaction	4.37		
**3f**	0.91	G2532	OH (eight membered ring)	H-bondingπ-π	1.75	163.3	−8.218
		A2089	pyrimidine ring	interaction	4.34		
**3g**	0.92	G2532	OH (eight membered ring)	H-bonding	1.67	177.0	−7.501
		G2088	C=O ester	H-bonding	2.15	131.9	
**3h**	0.91	G2532	OH (eight membered ring)	H-bonding	1.78	170.7	−8.483
		G2088	C=O ester	H-bonding	2.04	128.1	
**3i**	0.92	G2532	OH (eight membered ring)	H-bonding	2.02	170.6	−8.413
		G2088	C=O ester C24 C=O	H-bonding	2.20 2.7	140.0 123.6	
		A2089	C24 C=O	H-bonding	1.812.34	105.9	
			NH			137.6	

^a^ A predicted pose was considered successful if the RMSD between the predicted pose and the native ligand was less than 2.0 Å. ^b^ The H-binding and π-π interaction of distance. These bonding distance could evaluate their strong or weak interaction.

## Supplementary Materials

Samples of Compounds **2**, **4**, **5**, **3a**, **3b**, **3c**, **3d**, **3e**, **3f**, **3g**, **3h** and **3i** are available from Supplementary Materials.

## References

[1] Singh S.B., Young K., Silver L.L. (2017). What is an “Ideal” Antibiotic? Discovery Challenges and Path forward. Biochem. Pharmacol..

[2] Ai X., Pu X., Yi Y., Liu Y., Xu S., Liang J., Shang R. (2016). Synthesis and Pharmacological Evaluation of Novel Pleuromutilin Derivatives with Substituted Benzimidazole Moieties. Molecules.

[3] Kavanagh F., Hervey A., Robbins W.J. (1951). Antibiotic Substances from Basidiomycetes VIII. *Pleurotus Multilus* (Fr.) Sacc. and Pleurotus Passeckerianus Pilat. Proc. Natl. Acad. Sci. USA.

[4] Eyal Z., Matzov D., Krupkin M., Paukner S., Riedl R., Rozenberg H., Zimmerman E., Bashan A., Yonath A. (2016). A novel pleuromutilin antibacterial compound, its binding mode and selectivity mechanism. Sci. Rep..

[5] Novak R., Shlaes D.M. (2010). The pleuromutilin antibiotics: A new class for human use. Curr. Opin. Investig. Drugs.

[6] Riedl K. (1976). Studies on pleuromutilin and some of its derivatives. J. Antibiot..

[7] Bacque E., Pautrat F., Zard S.Z. (2002). A Flexible Strategy for the Divergent Modification of Pleuromutilin. Chem. Commun..

[8] Egger H., Reinshagen H. (1976). New pleuromutilin derivatives with enhanced antimicrobial activity. I. Synthesis. Jpn. J. Antibiot..

[9] Poulsen S.M., Karlsson M., Johansson L.B., Vester B. (2001). The pleuromutilin drugs tiamulin and valnemulin bind to the RNA at the peptidyl transferase centre on the ribosome. Mol. Microbiol..

[10] Schlünzen F., Pyetan E., Fucini P., Yonath A., Harms J.M. (2004). Inhibition of peptide bond formation by pleuromutilins: The structure of the 50S ribosomal subunit from Deinococcus radiodurans in complex with tiamulin. Mol. Microbiol..

[11] Chen D., Bashan A., Auerbach-Nevo T., Yaggie R.D., Gontarek R.R., Yonath A. (2007). Induced-Fit Tightens Pleuromutilins Binding to Ribosomes and Remote Interactions Enable Their Selectivity. Proc. Natl. Acad. Sci. USA.

[12] Eyal Z., Matzov D., Krupkin M., Wekselman I., Paukner S., Zimmerman E., Rozenberg H., Bashan A., Yonath A. (2015). Structural insights into species-specific features of the ribosome from the pathogen *Staphylococcus aureus*. Proc. Natl. Acad. Sci. USA.

[13] Thakare R., Dasgupta A., Chopra S. (2016). LEFAMULIN Pleuromutilin antibacterial, Treatment of pneumonia and ABSSI. Drugs Future.

[14] Barry A.L., Craig W.A., Nadler H., Reller L.B., Sanders C.C., Swenson J.M. (1999). Methods for determining bactericidal activity of antimicrobial agents: Approved guideline. NCCLS Document M26-A.

[15] Klich K., Pyta K., Kubicka M.M., Ruszkowski P., Celewicz L., Gajecka M., Przybylski P. (2016). Synthesis, Antibacterial, and Anticancer Evaluation of Novel Spiramycin-Like Conjugates Containing C(5) Triazole Arm. J. Med. Chem..

[16] Marialke J., Tietze S., Apostolakis J. (2008). Similarity Based Docking. J. Chem. Inf. Model..

[17] Domagalska J., Janas A., Pyta K., Pecyna P., Ruszkowski P., Celewicz L., Gajecka M., Bartl F., Przybylski P. (2016). 16-Membered Macrolide Lactone Derivatives Bearing a Triazole-Functionalized Arm at the Aglycone C13 Position as Antibacterial and Anticancer Agents. ChemMedChem.

[18] Wilson D.N. (2011). On the specificity of antibiotics targeting the large ribosomal subunit. Ann. N. Y. Acad. Sci..

[19] Hanwell M.D., Curtis D.E., Lonie D.C., Vandermeersch T., Zurek E., Hutchison G.R. (2012). Avogadro: An advanced semantic chemical editor, visualization, and analysis platform. J. Cheminformatics.

[20] The PyMOL User’s Manual. https://www.scienceopen.com/document?vid=602cf44e-e5ab-49bd-bb13-5f4fef98741d.

[21] The PyMOL Molecular Graphics System. http://www.mendeley.com/catalog/pymol-molecular-graphics-system-version-11/.

