# Supplementary material for: Synthesis and Biological Activity Evaluation of Novel Heterocyclic Pleuromutilin Derivatives
Source: Molecules. 2017 Jun 15;22(6):996. doi: 10.3390/molecules22060996 (PMC6152684; doi:10.3390/molecules22060996)
Supplement: Supplementary file 1 [file molecules-22-00996-s001.pdf]

Supplementary Materials: Synthesis and Biological Activity Evaluation of Novel Heterocyclic Pleuromutilin Derivatives

Yunpeng Yi, Yunxing Fu, Pengcheng Dong, Wenwen Qin, Yu Liu, Jiangping Liang, Ruofeng Shang

*14-O-(p-toluene sulfonyloxyacetyl) mutilin(**2**)*:

A 5 mL of NaOH aqueous solution (2 g, 50 mmol) was added dropwise to a mixture of pleuromutilin (7.57 g, 20 mmol) and p-toluenesulfonyl chloride (4.2 g, 22 mmol) in methyl isobutyl ketone (10 mL) and water (5 mL). The mixture was vigorously stirred for 45min at 60 ^oC, then the reaction mixture was cooled to 10 ^oC and separated. The organic layer was washed with 5 mL water and 5 mL saturated sodium carbonate solution. The organic phase was dried overnight with anhydrous sodium sulfate. After filteration, the solvent was concentrated in vacuo to give 10.56 g of yellow oil. It was used in the next step without further purification. Yield: 93%. IR (KBr): 3446 (OH), 2924 (CH_2), 2863 (CH_2), 1732 (C=O), 1633 (C-C), 1597 (C=C), 1456 (C=C), 1371 (CH_3), 1297 (C-O-C), 1233 (CH), 1117 (C-(C=O)-C), 1035 (C-O-C), 832 (CH), 664 (CH_2=), 560 (CH_2=) cm^{-1}. ^1H NMR (400 MHz, $CDCl_3$) δ 7.74 (d, J = 8.3 Hz, 2H), 7.26 (t, J = 13.3 Hz, 2H), 6.34 (dd, J = 17.4, 11.0 Hz, 1H), 5.70 (d, J = 8.5 Hz, 1H), 5.19 (dd, J = 55.1, 14.2 Hz, 2H), 4.47 – 4.33 (m, 2H), 3.28 (s, 1H), 2.38 (s, 3H), 2.25 – 2.09 (m, 3H), 2.01 (s, 1H), 1.99 – 1.88 (m, 1H), 1.71 – 1.62 (m, 1H), 1.61 – 1.51 (m, 2H), 1.46 – 1.38 (m, 2H), 1.35 (d, J = 7.8 Hz, 3H), 1.27 (d, J = 11.5 Hz, 1H), 1.18 (dd, J = 11.6, 4.5 Hz, 2H), 1.12 – 1.00 (m, 4H), 0.80 (d, J = 7.0 Hz, 3H), 0.55 (d, J = 7.0 Hz, 3H). ^{13}C NMR(100 MHz, $CDCl_3$) δ215.71 (C=O), 163.87 (C=O), 144.29 (benzene-C), 137.70 (CH=), 131.63 (benzene-C), 128.91 (benzene-C), 127.09 (benzene-C), 116.38 (CH_2=), 73.54 (CH), 69.29 (CH), 64.03 (CH), 57.02 (CH), 44.39 (C), 43.51 (CH_2), 42.97 (C), 40.84 (C), 35.54 (CH), 35.03 (CH), 33.40 (CH_2), 29.34 (CH_3), 25.77(CH_2), 25.39 (CH_2), 23.81 (CH_2), 20.68 (CH_3), 15.53 (CH_3), 13.76 (CH_3), 10.47 (CH_3). HRMS (ESI) calcd $[M+H]^+$ for $C_{29}H_{40}O_7S$ 533.250, found 533.2507.

*14-O-(acetic acidthioacetyl) mutilin(**4**)*:

Compound 3 was prepared by stirring a mixing of compound **2** (10 mmol), potassium thioglycolate (20 mmol) , methyl isobutyl ketone (30ml) in room temperature, and the mixture

was stirred for 2h. The mixtrue was extracted with water(10ml). The organic were combined,dried over Na_2SO_4, and concentrated to give compound 3. Yield: 80%. IR (KBr): 3448 (OH), 2967 (CH_2), 2924 (CH_3), 2865 (CH_2), 1731 (C=O), 1702 (C=O), 1453 (C-C), 1419, 1384 (CH_3), 1295 (C-O-C), 1183 (CH), 1154 (C-O), 1115 (C-(C=O)-C), 1017 (C-O-C) cm^{-1}. ^{1}H NMR (400 MHz, $CDCl_3$) δ 6.58 – 6.30 (m, 1H), 5.72 (d, J = 8.4 Hz, 1H), 5.43 – 5.14 (m, 2H), 3.63 (s, 2H), 3.36 (d, J = 6.4 Hz, 1H), 2.36 (dd, J = 13.5, 3.5 Hz, 3H), 2.35 – 2.27 (m, 1H), 2.21 (dd, J = 17.4, 7.9 Hz, 1H), 2.13 (d, J = 14.9 Hz, 1H), 2.04 (dd, J = 26.1, 17.5 Hz, 1H), 1.73 (dd, J = 31.8, 10.6 Hz, 2H), 1.68 – 1.62 (m, 2H), 1.62 – 1.41 (m, 6H), 1.41 – 1.24 (m, 2H), 1.24 – 0.99 (m, 4H), 0.89 (t, J = 8.4 Hz, 3H), 0.78 – 0.61 (m, 3H).^{13}C NMR (101 MHz, $CDCl_3$) δ 216.90 (C=O), 193.45 (C=O), 167.32 (C=O), 138.92 (CH=), 117.19 (CH_2=), 74.60 (CH), 70.09 (CH), 58.13 (CH), 45.45 (C), 44.71 (CH_2), 44.01 (C), 41.89 (C), 36.74 (CH), 36.00 (CH_3), 34.45 (CH_2), 32.20 (CH_2), 30.42 (CH_2), 30.06 (CH_2), 26.84 (CH_2), 26.41 (CH_3), 24.82 (CH_2), 16.74 (CH_3), 14.81(CH_3), 11.43 (CH_3). HRMS (ESI) calcd [M+H]+ for $C_{24}H_{36}O_5S$ 437.2356, found 437.2339.

*(R)-5-ChloroMethyl-2-oxazolidinone(**5**)*:

The title compound was prepared by stirring a mixing of magnesium sulphate (10 mmol), Sodium Cyanate (10 mmol), water (50ml) in room temperature. (R)-(-)-Epichlorohydrin to the solution (5mmol) was added dropwise to the mixture. The result mixture was stirred under 60° C for 1h.The reaction mixture was concentrated in vacuo and extracted by ethyl acetate. The two layers were separated, and the organic layer was dried over Na_2SO_4, filtered, and concentrated to dryness. Yield: 57%. IR (KBr): 3365 (NH), 1744 (C=O), 1429 (C-N), 1240 (C-C(=O)-O), 736 (C-Cl) cm^{-1}. ^{1}H NMR (400 MHz, DMSO) δ 7.60 (s, 1H), 4.84 (dd, J = 9.6, 4.7 Hz, 1H), 4.04 – 3.73 (m, 2H), 3.59 (t, J = 9.0 Hz, 1H), 3.25 (dd, J = 9.0, 6.2 Hz, 1H).^{13}C NMR (101 MHz, d_6-DMSO) δ 158.68 (C=O), 74.35 (CH), 46.66 (CH_2), 43.03(CH_2). HRMS (ESI) calcd $[M+H]^+$ for C_4H_6ClNO 136.0159, found 136.0150.

*General Procedure for Synthesis of Compounds **3a-3g***

A mixing of thiols (1mmol), sodium hydroxide (1.1mmol), water (0.5ml) and methanol (3ml) were stirred in room temperature. After 30 minute, compound **2** (1.1mmol) in 5ml CH_2Cl_2 was added dropwise to the mixture for 36h-42h. The mixture was concentrated in vacuo. The residue was dissolved by CH_2Cl_2. The solution was extracted three times with water. The organic phase was dried overnight with anhydrous sodium sulfate. The solvent was concentrated in vacuo to give

crude products. The crude product was purified by silica gel column chromatography.

*14-O-[(4-amino-pyrimidinone-2-yl) thioacetyl] mutilin (**3a**)*:

Compound **3a** was prepared according to the general procedure from 14-O-(p-toluene sulfonyloxyacetyl) mutilin(**2**) and 4-amino-2-Pyrimidinone. The crude product was purified over silica gel column chromatography to give 4.09 g. Yield: 84%. IR (KBr): 3448 (OH), 2933 (CH_2), 1730 (C=O), 1629 (C-C), 1583 (C=N), 1543 (C=C), 1467 (C=C), 1372 (CH_3), 1249(C-C(=O)-O), 1153(C-O), 1117 (C-(C=O)-C), 1018 (C-O-C) cm^{-1}. ^1H NMR (400 MHz, $CDCl_3$) δ 7.89 (d, *J* = 5.1 Hz, 1H), 6.42 (dd, *J* = 17.1, 11.2 Hz, 1H), 6.04 (d, *J* = 5.2 Hz, 1H), 5.68 (d, *J* = 7.9 Hz, 1H), 5.17 (dd, *J* = 51.9, 14.0 Hz, 2H), 4.94 (s, 2H), 3.72 (dd, *J* = 32.8, 16.1 Hz, 2H), 3.28 (s, 1H), 2.24 (d, *J* = 6.5 Hz, 1H), 2.14 (dd, *J* = 14.8, 9.1 Hz, 2H), 2.02 (s, 1H), 1.94 (dd, *J* = 15.6, 8.5 Hz, 1H), 1.69 (d, *J* = 13.7 Hz, 1H), 1.61 – 1.52 (m, 2H), 1.48 (d, *J* = 12.0 Hz, 2H), 1.37 (s, 4H), 1.32 – 1.22 (m, 2H), 1.07 (s, 4H), 0.79 (d, *J* = 6.2 Hz, 3H), 0.68 (d, *J* = 6.2 Hz, 3H). ^{13}C NMR (101 MHz, $CDCl_3$) δ 216.11 (C=O), 168.72 (pyrimidine-C), 167.20 (C=O), 161.35 (pyrimidine-C), 154.88 (pyrimidine-C), 138.26 (CH=), 116.02 (CH_2=), 100.18 (pyrimidine-C), 73.60 (CH), 68.51(CH), 57.36 (CH), 57.21(CH_2), 44.46 (CH_2), 43.47 (C), 42.93 (C), 40.87 (CH), 35.81 (CH), 35.02 (CH), 33.48 (CH_2), 33.06 (CH_2), 29.45(CH_2), 25.89 (CH_3), 23.84 (CH_2), 15.74 (CH_3), 13.92 (CH_3), 10.44 (CH_3). HRMS (ESI) calcd $[M+H]^+$ for $C_{26}H_{37}N_3O_4S$ 488.2578, found 488.2570.

*14-O-[(4-methylpyrimidine-2-yl) thioacetyl] mutilin (**3b**)*:

Compound **3b** was prepared according to the general procedure from 14-O-(p-toluene sulfonyloxyacetyl) mutilin(**2**) and 4-methy-2-pyrimidinone. The crude product was purified over silica gel column chromatography to give3.55 g. Yield: 73%. IR (KBr): 3439 (OH), 2935 (CH_2), 1733 (C=O), 1658 (C-C), 1580 (C=N), 1535 (C=C), 1458 (C=C), 1396 (CH_3), 1285 (C-O-C), 1118 (C-(C=O)-C) cm^{-1}. ^1H NMR (400 MHz, $CDCl_3$) δ 6.37 (dt, J = 33.7, 16.8 Hz, 1H), 5.99 (s, 1H), 5.69 (d, J = 8.4 Hz, 1H), 5.19 (dd, J = 56.5, 14.2 Hz, 2H), 3.88 – 3.73 (m, 2H), 3.29 (d, J = 5.6 Hz, 1H), 2.27 – 2.15 (m, 2H), 2.11 (d, J = 13.1 Hz, 3H), 2.02 (d, J = 6.4 Hz, 1H), 1.96 (d, J = 10.9 Hz, 1H), 1.69 (d, J = 14.2 Hz, 1H), 1.58 (dd, J = 21.0, 10.8 Hz, 2H), 1.49 (dd, J = 26.7, 13.3 Hz, 2H), 1.43 – 1.26 (m, 6H), 1.20 (dd, J = 17.5, 11.2 Hz, 2H), 1.05 (d, J = 20.9 Hz, 4H), 0.80 (d, J = 6.9 Hz, 3H), 0.67 (d, J = 6.9 Hz, 3H). ^{13}C NMR (101 MHz, $CDCl_3$) δ 215.94 (C=O), 165.80(C=O), 164.69 (pyrimidine-C), 164.11 (pyrimidine-C), 157.85 (pyrimidine-C), 137.90 (CH=), 116.27 (CH_2=), 107.63 (pyrimidine-C), 73.55 (CH), 69.14 (CH), 57.08 (CH), 44.43 (C),

43.49 (CH_2), 42.94 (C), 40.87 (C), 35.70 (CH), 35.00 (CH_3), 33.44 (CH_2), 32.23 (CH_2), 29.39 (CH_2), 25.84(CH_3), 25.36(CH_2), 23.82 (CH_2), 23.09 (CH_2), 15.84(CH_3), 13.84 (CH_3), 10.46 (CH_3). HRMS (ESI) calcd $[M+H]^+$ for $C_{27}H_{38}N_2O_4S$ 487.2625, found 487.2623.

*14-O-[(benzimidazole-2-yl) thioacetyl] mutilin (**3c**)*:

Compound **3c** was prepared according to the general procedure from 14-O-(p-toluene sulfonyloxyacetyl) mutilin(**2**) and 2-mercaptobenzothiazole. The crude product was purified over silica gel column chromatography to give 3.54 g. Yield: 67%. IR (KBr): 3442 (OH), 2929 (CH_2), 1731 (C=O), 1458 (C=C), 1429 (C-C), 1274 (C-O-C), 1153 (C-O), 1117 (C-(C=O)-C) cm^{-1}. ^1H NMR (400 MHz, $CDCl_3$) δ 7.77 (dd, *J* = 19.9, 8.0 Hz, 2H), 7.39 (t, *J* = 7.7 Hz, 1H), 7.29 (t, *J* = 7.6 Hz, 1H), 6.42 (dd, *J* = 17.4, 11.0 Hz, 1H), 5.76 (d, *J* = 8.5 Hz, 1H), 5.19 (dd, *J* = 57.6, 14.2 Hz, 2H), 4.08 (dd, *J* = 41.4, 16.2 Hz, 2H), 3.31 (d, *J* = 6.4 Hz, 1H), 2.29 (dd, *J* = 14.1, 7.2 Hz, 1H), 2.20 (dd, *J* = 11.6, 6.3 Hz, 1H), 2.06 (s, 1H), 1.98 (dd, *J* = 16.0, 8.5 Hz, 1H), 1.82 – 1.64 (m, 2H), 1.64 – 1.51 (m, 2H), 1.50 – 1.33 (m, 6H), 1.24 (dd, *J* = 14.3, 7.4 Hz, 2H), 1.13 – 0.97 (m, 4H), 0.85 (d, *J* = 7.0 Hz, 3H), 0.77 (d, *J* = 6.9 Hz, 3H). ^{13}C NMR (101 MHz, $CDCl_3$) δ 215.94 (C=O), 165.84 (C=O), 163.54 (benzothiazole-C), 151.74 (benzothiazole-C), 137.76 (CH=), 134.49(benzothiazole-C), 125.02 (benzothiazole-C), 123.46 (benzothiazole-C), 120.68 (benzothiazole-C), 120.05 (benzothiazole-C), 116.21 (CH_2=), 73.57 (CH), 69.20 (CH), 57.10 (CH), 44.42 (C), 43.41 (CH_2), 42.90 (C), 40.86 (C), 35.74 (CH), 34.98 (CH), 34.67 (CH_2), 33.43(CH_2), 29.40 (CH_2), 25.84 (CH_2), 25.28 (CH_3), 23.81 (CH_2), 15.80(CH_3), 13.81 (CH_3), 10.44 (CH_3). HRMS (ESI) calcd $[M+H]^+$ for $C_{29}H_{37}NO_4S$ 528.2237, found 528.2234.

*14-O-[(benzothiazole-2-yl) thioacetyl] mutilin (**3d**)*:

Compound **3d** was prepared according to the general procedure from 14-O-(p-toluene sulfonyloxyacetyl) mutilin(**2**) and 2-mercaptobenzimidazole. The crude product was purified over silica gel column chromatography to give 3.68 g. Yield 72%. IR (KBr): 3423 (OH), 2925 (CH_2), 1726 (C=O), 1458 (C=C), 1439 (C-C), 1271 (C-O-C), 1152 (C-O), 1117 (C-(C=O)-C) cm^{-1}. ^1H NMR (400 MHz, $CDCl_3$) δ 7.50 (dd, *J* = 5.8, 3.1 Hz, 2H), 7.20 (dd, *J* = 6.0, 3.2 Hz, 2H), 6.41 (dd, *J* = 17.4, 11.0 Hz, 1H), 5.79 (d, *J* = 8.4 Hz, 1H), 5.18 (dd, *J* = 43.7, 14.2 Hz, 2H), 3.91 (s, 2H), 3.73 (q, *J* = 7.0 Hz, 1H), 3.35 (d, *J* = 6.4 Hz, 1H), 2.35 – 2.28 (m, 1H), 2.27 – 2.17 (m, 1H), 2.08 (s, 1H), 2.03 (dd, *J* = 16.1, 8.6 Hz, 1H), 1.82 – 1.65 (m, 2H), 1.64 – 1.52 (m, 2H), 1.49 – 1.32 (m, 6H), 1.24 (t, *J* = 7.0 Hz, 2H), 1.16 – 1.05 (m, 4H), 0.88 (d, *J* = 6.9 Hz, 3H), 0.72 (d, *J* = 7.0 Hz,

3H). ^{13}C NMR (101 MHz, $CDCl_3$) δ 215.89 (C=O), 167.78 (C=O), 147.20 (benzimidazole-C), 137.75 (CH=), 121.68 (benzimidazole-C), 116.28 (CH_2=), 73.61 (CH), 69.82 (CH), 57.43 (CH), 57.09 (benzimidazole-C), 44.42 (CH_2), 43.52 (C), 42.97 (C), 40.84 (C), 35.68 (CH), 35.04 (CH_2), 34.16 (CH_2), 33.42, 29.38 (CH_2), 25.84 (CH_2), 25.44 (CH_3), 23.83 (CH_2), 17.42 (CH_2), 15.80 (CH_3), 13.80 (CH_3), 10.50 (CH_3). HRMS (ESI) calcd $[M+H]^+$ for $C_{29}H_{38}N_2O_4S$ 511.2526, found 511.2531.

*14-O-[(5-benzimidazolesulfonate-2-yl) thioacetyl] mutilin (**3e**)*:

Compound **3e** was prepared according to the general procedure from 14-O-(p-toluene sulfonyloxyacetyl) mutilin(**2**) and sodium 2-mercapto-5-benzimidazolesulfonate dihydrate. The crude product was purified over silica gel column chromatography to give 3.2 g. Yield: 54.2%. IR (KBr): 3448 (OH), 2940 (CH_2), 1732 (C=O) 1298 (C-O-C), 1190 (S=O), 1178 (S=O) cm^{-1}. ^{1}H NMR (400 MHz, DMSO) δ 7.80 (s, 1H), 7.69 (d, J = 8.5 Hz, 1H), 7.60 (d, J = 8.5 Hz, 1H), 6.04 (dd, J = 17.8, 11.2 Hz, 1H), 5.50 (d, J = 8.1 Hz, 1H), 4.96 (dd, J = 38.2, 14.5 Hz, 2H), 4.38 (d, J = 4.4 Hz, 1H), 3.36 (d, J = 5.5 Hz, 1H), 2.51 (s, 1H), 2.35 (s, 1H), 2.13 (dd, J = 21.0, 10.7 Hz, 1H), 2.08 – 1.96 (m, 2H), 1.90 (dd, J = 16.0, 8.1 Hz, 1H), 1.60 (s, 2H), 1.44 (d, J = 6.9 Hz, 1H), 1.33 (d, J = 7.6 Hz, 2H), 1.27 – 1.12 (m, 6H), 1.10 – 1.02 (m, 1H), 1.02 – 0.90 (m, 4H), 0.78 (d, J = 6.8 Hz, 3H), 0.58 (t, J = 9.3 Hz, 3H). ^{13}C NMR (101 MHz, DMSO) δ 217.48 (C=O), 166.34 (C=O), 150.52 (benzimidazole-C), 145.67 (benzimidazole-C), 141.16 (benzimidazole-C), 133.41 (CH=), 128.59 (benzimidazole-C), 125.97 (benzimidazole-C), 123.22 (benzimidazole-C), 115.68 (CH2=), 113.22 (CH_2), 110.71 (benzimidazole-C), 72.92 (CH), 71.44 (CH), 57.42 (CH), 45.35 (C), 44.58 (CH_2), 41.95 (C), 36.89 (CH), 36.64 (CH), 35.03 (CH_2), 34.40 (CH_2), 30.53 (CH_2), 29.06 (CH_2), 26.99 (CH_2), 24.85 (CH_2), 16.47 (CH_3), 14.64 (CH_3), 11.96 (CH_3). HRMS (ESI) calcd $[M+H]^+$ for $C_{29}H_{38}N_2O_7S_2$ 591.2193, found 591.2192.

*14-O-[(pyryrazolo[3,4d]pyrimidine-4-yl) thioacetyl] mutilin (**3f**)*:

Compound **3f** was prepared according to the general procedure from 14-O-(p-toluene sulfonyloxyacetyl) mutilin(**2**) and 4-mercaptopyrazolo[3,4-d]pyrimidine. The crude product was purified over silica gel column chromatography to give 4.30g. Yield: 84%. IR (KBr): 3431 (OH), 2934 (CH_2), 1732 (C=O), 1567 (C=C), 1456 (C=C), 1406 (C-C), 1271 (C-O-C), 1152 (C-O), 1117 (C-(C=O)-C), 981 (CH) cm^{-1}. ^{1}H NMR (400 MHz, $CDCl_3$) δ 8.65 (d, J = 37.7 Hz, 1H), 8.29 – 8.08 (m, 1H), 6.43 (dt, J = 21.8, 10.9 Hz, 1H), 5.80 (d, J = 8.4 Hz, 1H), 5.38 – 5.29 (m, 1H), 5.24 (dd, J

= 33.0, 14.5 Hz, 2H), 4.18 – 4.02 (m, 2H), 3.38 (d, *J* = 6.3 Hz, 1H), 2.31 (dd, *J* = 13.9, 7.1 Hz, 1H), 2.22 (dd, *J* = 13.0, 7.5 Hz, 1H), 2.11 (t, *J* = 8.4 Hz, 1H), 2.04 (d, *J* = 7.9 Hz, 1H), 1.73 (dd, *J* = 31.9, 9.2 Hz, 2H), 1.65 (dd, *J* = 17.1, 7.1 Hz, 2H), 1.58 – 1.37 (m, 6H), 1.36 – 1.22 (m, 2H), 1.19 – 1.06 (m, 4H), 0.88 (t, *J* = 11.3 Hz, 3H), 0.79 (t, *J* = 12.6 Hz, 3H). ^{13}C NMR (101 MHz, $CDCl_3$) δ 216.06 (C=O), 166.18 (C=O), 162.56 (pyrimidine-C), 153.05 (pyrimidine-C), 151.46 (pyrimidine-C), 137.88 (CH=), 131.82 (pyrazolo-C), 116.28 (CH_2=), 110.65 (pyrimidine-C), 73.59 (CH), 69.22 (CH), 57.14 (CH), 44.46 (C), 43.57 (CH_2), 42.92 (C), 40.88 (C), 35.75 (CH), 35.01 (CH_2), 33.46 (CH_2), 31.11 (CH_2), 29.41 (CH_2), 25.44 (CH_3), 23.83 (CH_2), 17.40(CH_2), 15.74 (CH_3), 13.86 (CH_3), 10.47 (CH_3). HRMS (ESI) calcd $[M+H]^+$ for $C_{27}H_{36}N_4O_4S$ 513.2530, found 513.2527.

14-O-[(furfuryl-2-yl*) thioacetyl] mutilin (**3g**)*:

Compound **3g** was prepared according to the general procedure from 14-O-(p-toluene sulfonyloxyacetyl) mutilin (**2**) and furfuryl mercaptan. The crude product was purified over silica gel column chromatography to give 3.53 g. Yield: 74%. IR (KBr): 3547 (OH), 2933 (CH_2), 2882 (CH2), 1731 (C=O), 1455 (C=C), 1281 (C-O-C), 1150 (C-O), 1115 (C-(C=O)-C) cm^{-1}. ^{1}H NMR (400 MHz, $CDCl_3$) δ 7.29 (s, 1H), 6.42 (dd, *J* = 17.4, 11.0 Hz, 1H), 6.31 – 6.06 (m, 2H), 5.71 (d, *J* = 8.4 Hz, 1H), 5.23 (dd, *J* = 57.0, 14.2 Hz, 2H), 3.75 (s, 2H), 3.30 (s, 1H), 3.02 (s, 2H), 2.29 (dd, *J* = 13.7, 6.8 Hz, 1H), 2.15 (dt, *J* = 19.6, 8.8 Hz, 2H), 2.09 – 1.98 (m, 2H), 1.75 – 1.66 (m, 1H), 1.64 – 1.55 (m, 2H), 1.53 – 1.35 (m, 6H), 1.30 (t, *J* = 14.9 Hz, 2H), 1.17 – 1.05 (m, 4H), 0.82 (d, *J* = 7.0 Hz, 3H), 0.68 (d, *J* = 6.8 Hz, 3H). ^{13}C NMR (101 MHz, $CDCl_3$) δ 215.99 (C=O), 167.67 (C=O), 149.27 (furan-C), 141.51(furan-C), 138.12 (CH=), 116.19 (CH_2=), 109.37 (furan-C), 107.39 (furan-C), 73.65 (CH), 68.31 (CH), 57.21 (CH), 44.46 (C), 43.85 (CH_2), 42.94 (C), 40.77 (C), 35.78 (CH), 35.04 (CH), 33.45 (CH_2), 32.11 (CH_2), 29.44 (CH_2), 27.36 (CH_2), 25.85 (CH_3), 25.42 (CH_2), 23.86 (CH_2), 15.81 (CH_3), 13.92 (CH_3), 10.49 (CH_3). HRMS (ESI) calcd $[M+H]^+$ for $C_{27}H_{38}O_5S$ 475.2513, found 475.2522.

14-O-[(1-methylimidazole *-2-yl) thioacetyl] mutilin (**3h**)*:

Compound **3h** was prepared according to the general procedure from 14-O-(p-toluene sulfonyloxyacetyl) mutilin(**2**) and 2-mercapto-1-methylimidazole. The crude product was purified over silica gel column chromatography to give 3.46 g. Yield: 73%. IR (KBr): 3423 (OH), 2924 (CH_2), 2863 (CH_2), 1717 (C=O), 1455 (C=C), 1410 (C-C), 1280 (C-O-C), 1145 (C-O), 1117

(C-(C=O)-C) cm^{-1}. ^{1}H NMR (400 MHz, $CDCl_3$) δ 6.88 (d, J = 49.1 Hz, 2H), 6.35 (dd, J = 17.4, 11.0 Hz, 1H), 5.63 (d, J = 8.5 Hz, 1H), 5.32 – 5.01 (m, 2H), 3.92 – 3.62 (m, 2H), 3.56 (d, J = 11.0 Hz, 3H), 3.27 (s, 1H), 2.22 (dt, J = 13.8, 7.0 Hz, 1H), 2.19 – 2.07 (m, 2H), 2.03 (d, J = 21.6 Hz, 1H), 1.93 (dd, J = 16.0, 8.6 Hz, 1H), 1.77 – 1.61 (m, 1H), 1.58 – 1.46 (m, 3H), 1.45 – 1.35 (m, 2H), 1.31 (d, J = 14.3 Hz, 3H), 1.26 – 1.13 (m, 2H), 1.12 – 0.97 (m, 4H), 0.80 (d, J = 7.0 Hz, 3H), 0.59 (d, J = 6.9 Hz, 3H). ^{13}C NMR (101 MHz, $CDCl_3$) δ 215.93 (C=O), 166.75 (C=O), 139.06 (imidazole-C), 138.01 (CH=), 128.55 (imidazole-C), 121.39 (imidazole-C), 116.07 (CH_2=), 73.57 (CH), 68.77 (CH), 57.12 (CH), 44.42 (C), 43.48 (CH_2), 42.94 (C), 40.77 (C), 36.08 (CH), 35.70 (CH_2), 35.00 (CH_3), 33.44 (CH_2), 32.35 (CH_2), 29.40 (CH_2), 25.83 (CH_2), 25.41(CH_3), 23.82 (CH_2), 15.61 (CH_3), 13.79 (CH_3), 10.44 (CH_3). HRMS (ESI) calcd $[M+H]^+$ for $C_{26}H_{38}N_2O_4S$ 475.2625, found 475.2630.

*14-O-(2-oxazolidinone, 5-(methyl)-) (thioacetyl) mutilin(**3i**)*:

A mixing of (R)-5-chloroMethyl-2-oxazolidinone (**5**) (1mmol), Sodiumiodide (0.1mmol), Acetone (10ml) were stirred in room temperature. After 30 minute, the reaction solution was filtered and concencrated. Compound **4** (1.1mmol) and triethylamine (20ml) was added under N_2. The solvent was stirred in 40 °C for 10h. The mixtrue was extracted with water (10ml) and HCl (2N, 10ml). The organic layers were concentrated in vacuo to give crude products. The crude product was purified by silica gel column chromatography. Yield: 62%. IR (KBr): 3422 (O), 2933 (CH_2), 1735 (C=O), 1686 (C=O), 1458 (C=C), 1420 (C-C), 1284 (C-O-C), 1151 (C-O), 1117 (C-(C=O)-C) cm^{-1}. ^{1}H NMR (400 MHz, $CDCl_3$) δ 6.63 – 6.35 (m, 1H), 6.17 (d, *J* = 8.2 Hz, 1H), 5.75 (d, *J* = 8.2 Hz, 1H), 5.27 (dd, *J* = 51.9, 14.2 Hz, 2H), 4.92 – 4.67 (m, 1H), 3.82 – 3.62 (m, 1H), 3.45 – 3.32 (m, 2H), 3.30 – 3.07 (m, 2H), 2.99 (ddd, *J* = 9.9, 8.9, 4.4 Hz, 1H), 2.87 (dt, *J* = 12.8, 5.2 Hz, 1H), 2.34 (s, 1H), 2.28 – 2.17 (m, 2H), 2.11 (s, 1H), 2.08 (d, *J* = 8.6 Hz, 1H), 1.77 (d, *J* = 14.3 Hz, 1H), 1.65 (d, *J* = 10.4 Hz, 2H), 1.52 (dd, *J* = 25.3, 6.7 Hz, 2H), 1.44 (d, *J* = 1.0 Hz, 4H), 1.39 (s, 1H), 1.35 – 1.26 (m, 1H), 1.16 (d, *J* = 14.9 Hz, 4H), 0.89 (d, *J* = 6.8 Hz, 3H), 0.73 (d, *J* = 6.8 Hz, 3H).^{13}C NMR (101 MHz, $CDCl_3$) δ 216.99 (C=O), 168.63 (C=O), 159.41 (C=O), 139.16 (CH=), 117.14 (CH_2=), 75.66 (CH), 74.61 (C), 69.70 (CH), 58.17 (CH), 45.46 (C), 45.06 (CH_2), 44.88 (CH_2), 43.95 (C), 41.78 (C), 36.75 (CH), 36.03 (CH), 35.99, 34.81 (CH), 34.45 (CH_2), 30.42 (CH_2), 26.86 (CH_3), 26.41 (CH_2), 24.84 (CH_2), 16.82 (CH_3), 14.88 (CH_3), 11.48 (CH_3). HRMS (ESI) calcd $[M+Na]^+$ for $C_{26}H_{39}NO_6S$ 516.2395, found 516.2394.

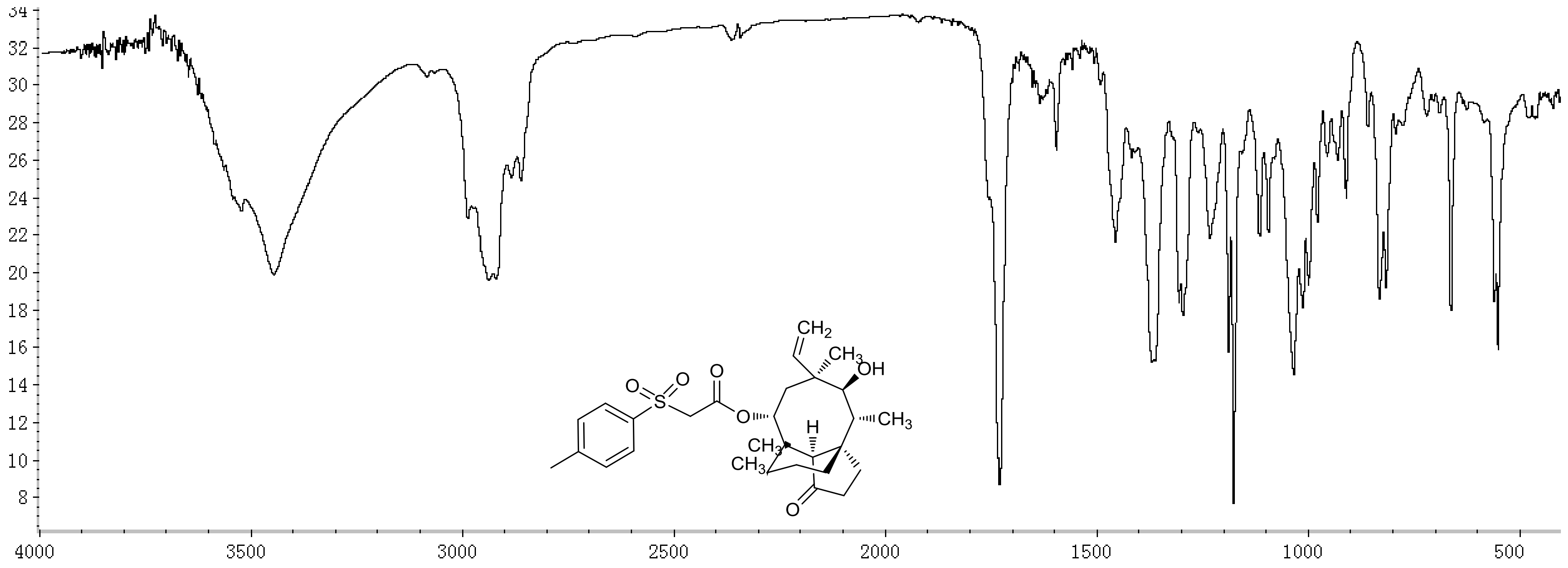
34
32
30
28
26
24
22
20
18
16
14
12
10
8
4000
3500
3000
2500
2000
1500
1000
500
CH2
CH3
OH
O
O
S
O
O
CH3
H
CH3
CH3
O

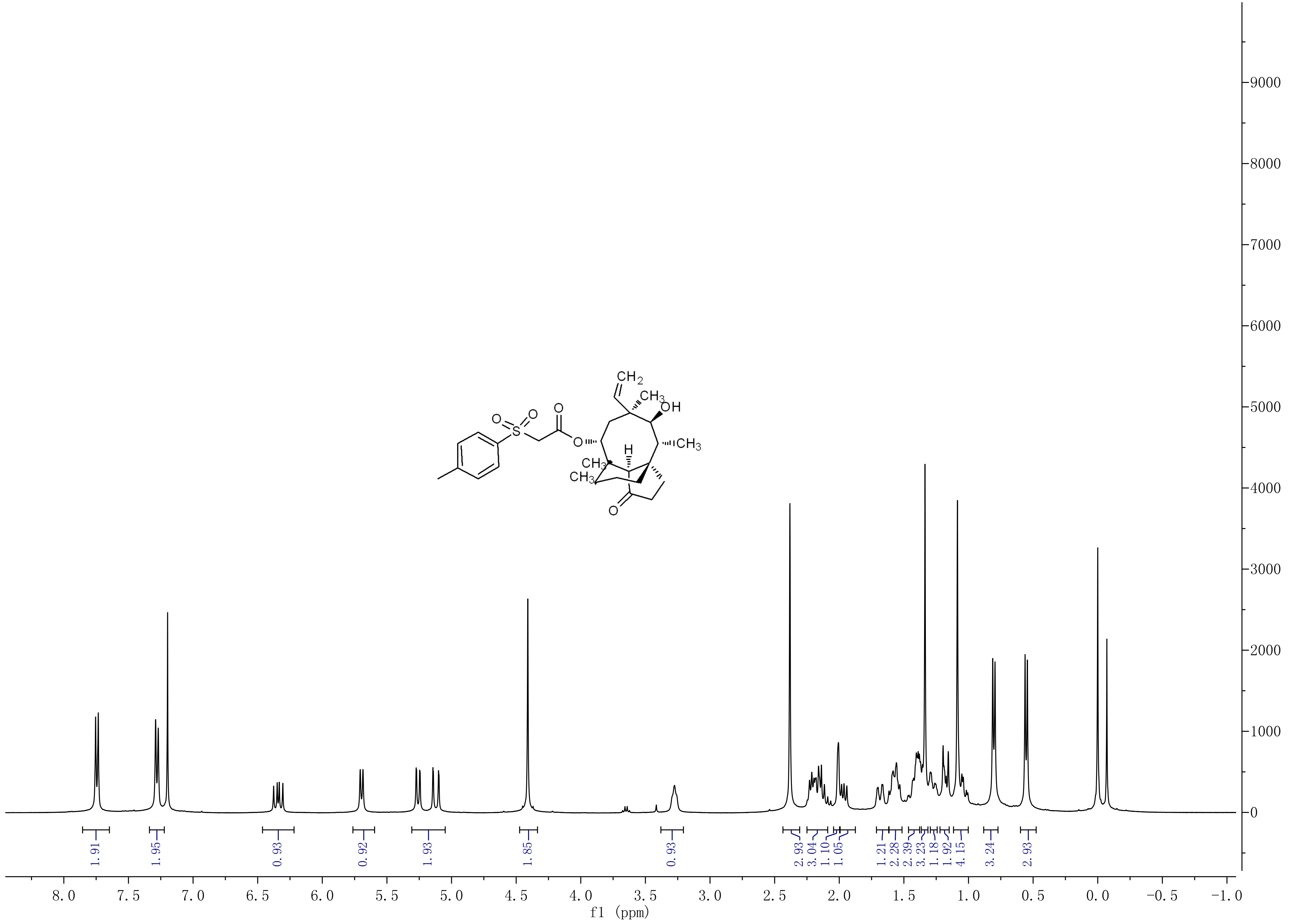
CH2
CH3
OH
CH3
O
O
S
O
O
H
CH3
CH3
O
1.91
1.95
0.93
0.92
1.93
1.85
0.93
2.93
3.04
1.10
1.05
1.21
2.28
2.39
3.23
1.18
1.92
4.15
3.24
2.93
8.0 7.5 7.0 6.5 6.0 5.5 5.0 4.5 4.0 3.5 3.0 2.5 2.0 1.5 1.0 0.5 0.0 -0.5 -1.0
f1 (ppm)
9000
8000
7000
6000
5000
4000
3000
2000
1000
0

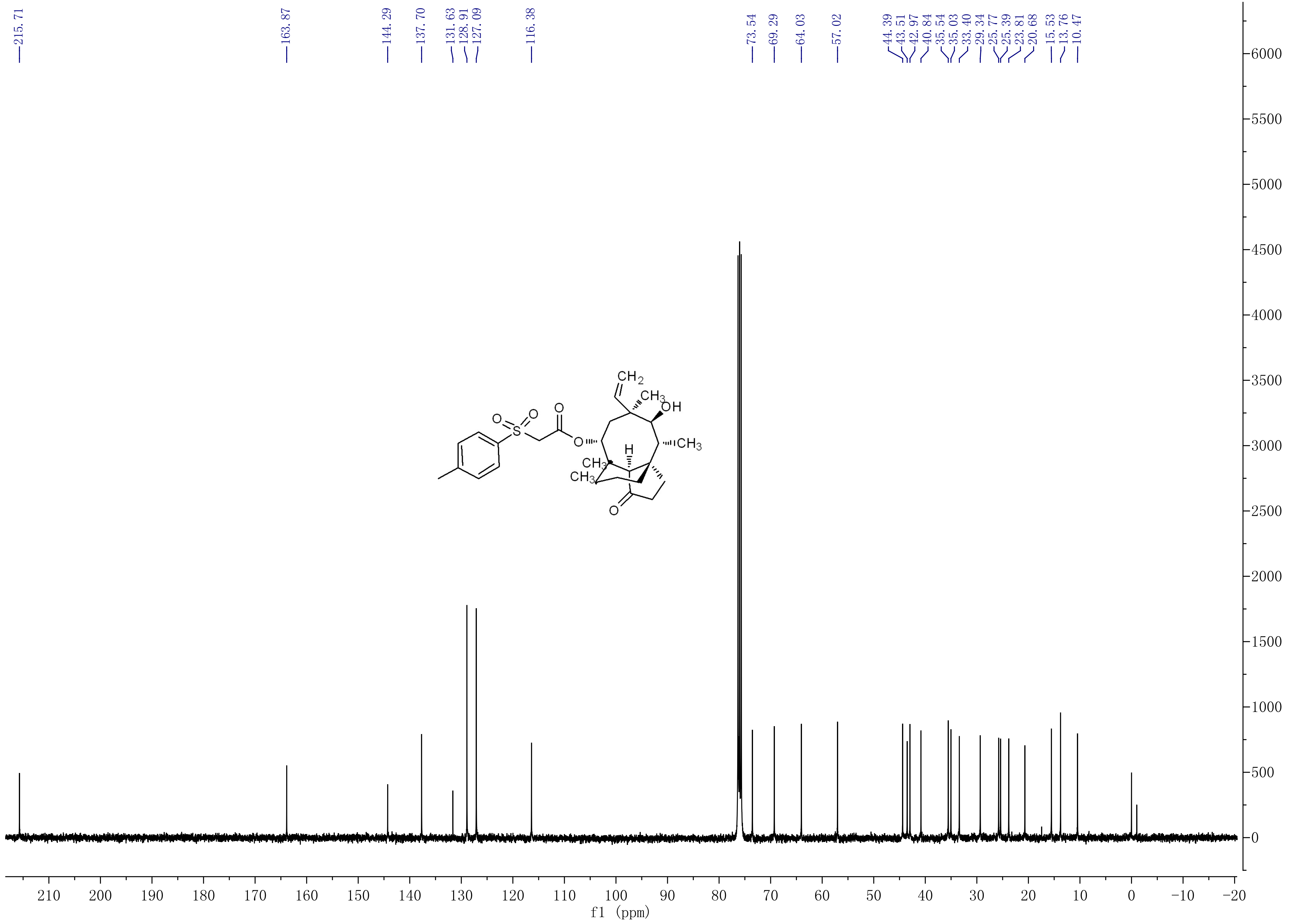

215.71
163.87
144.29
137.70
131.63
128.91
127.09
116.38
73.54
69.29
64.03
57.02
44.39
43.51
42.97
40.84
35.54
35.03
33.40
29.34
25.77
25.39
23.81
20.68
15.53
13.76
10.47
CH2
CH3
OH
O
S
CH3
H
CH3
CH3
O
f1 (ppm)

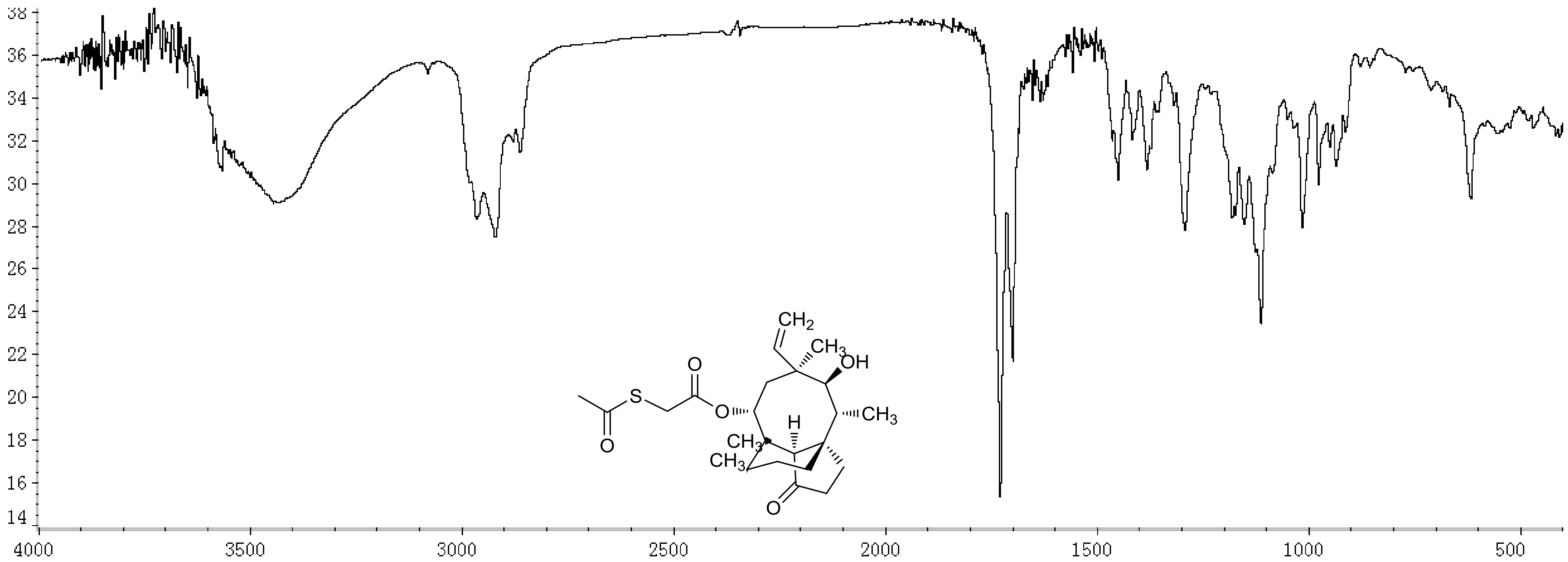

38
36
34
32
30
28
26
24
22
20
18
16
14
4000
3500
3000
2500
2000
1500
1000
500
CH2
CH3
OH
O
S
O
CH3
H
CH3
CH3
O

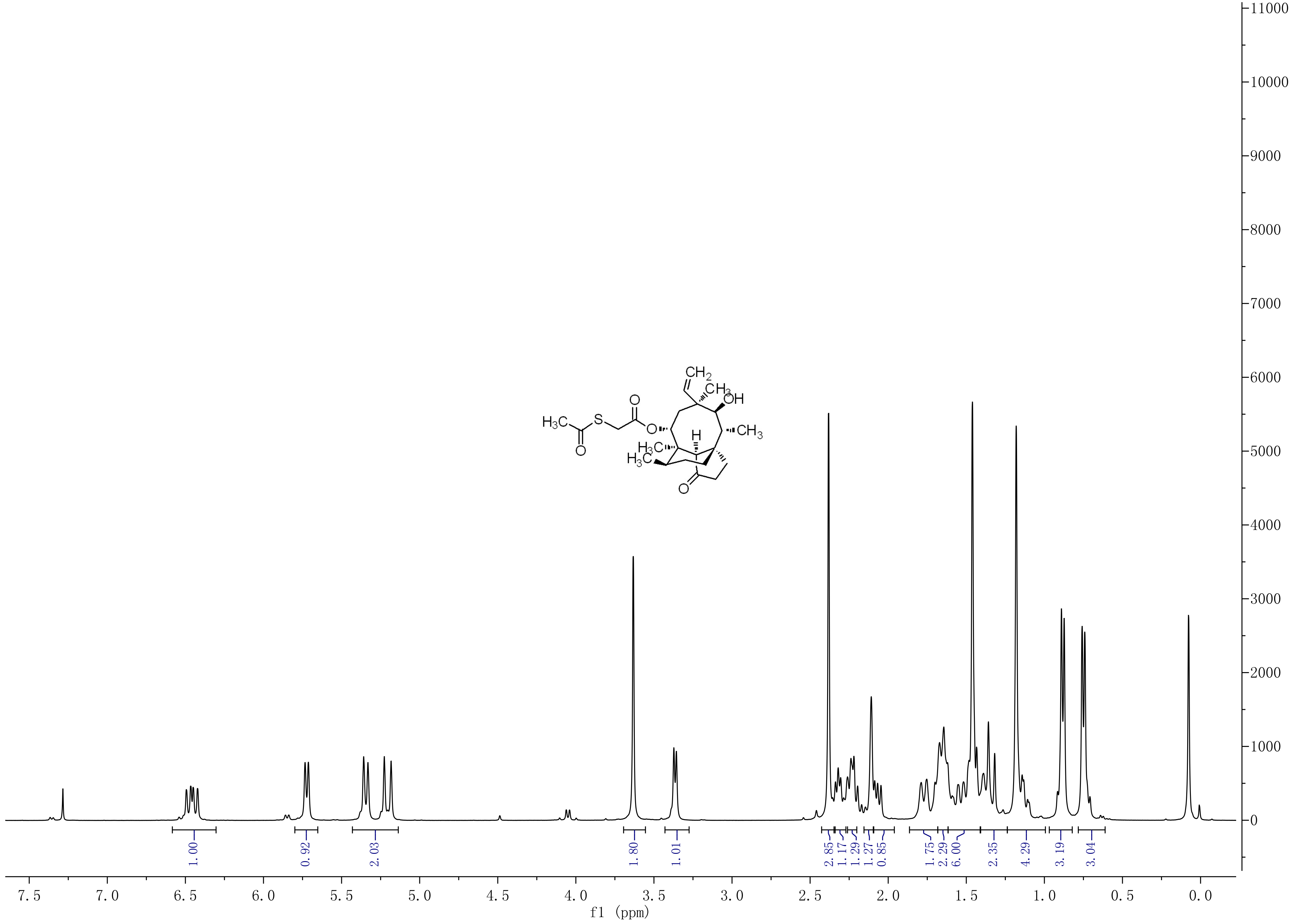

11000
10000
9000
8000
7000
6000
5000
4000
3000
2000
1000
0
1.00
0.92
2.03
1.80
1.01
2.85
1.17
1.29
1.27
0.85
1.75
2.29
6.00
2.35
4.29
3.19
3.04
7.5
7.0
6.5
6.0
5.5
5.0
4.5
4.0
3.5
3.0
2.5
2.0
1.5
1.0
0.5
0.0
f1 (ppm)

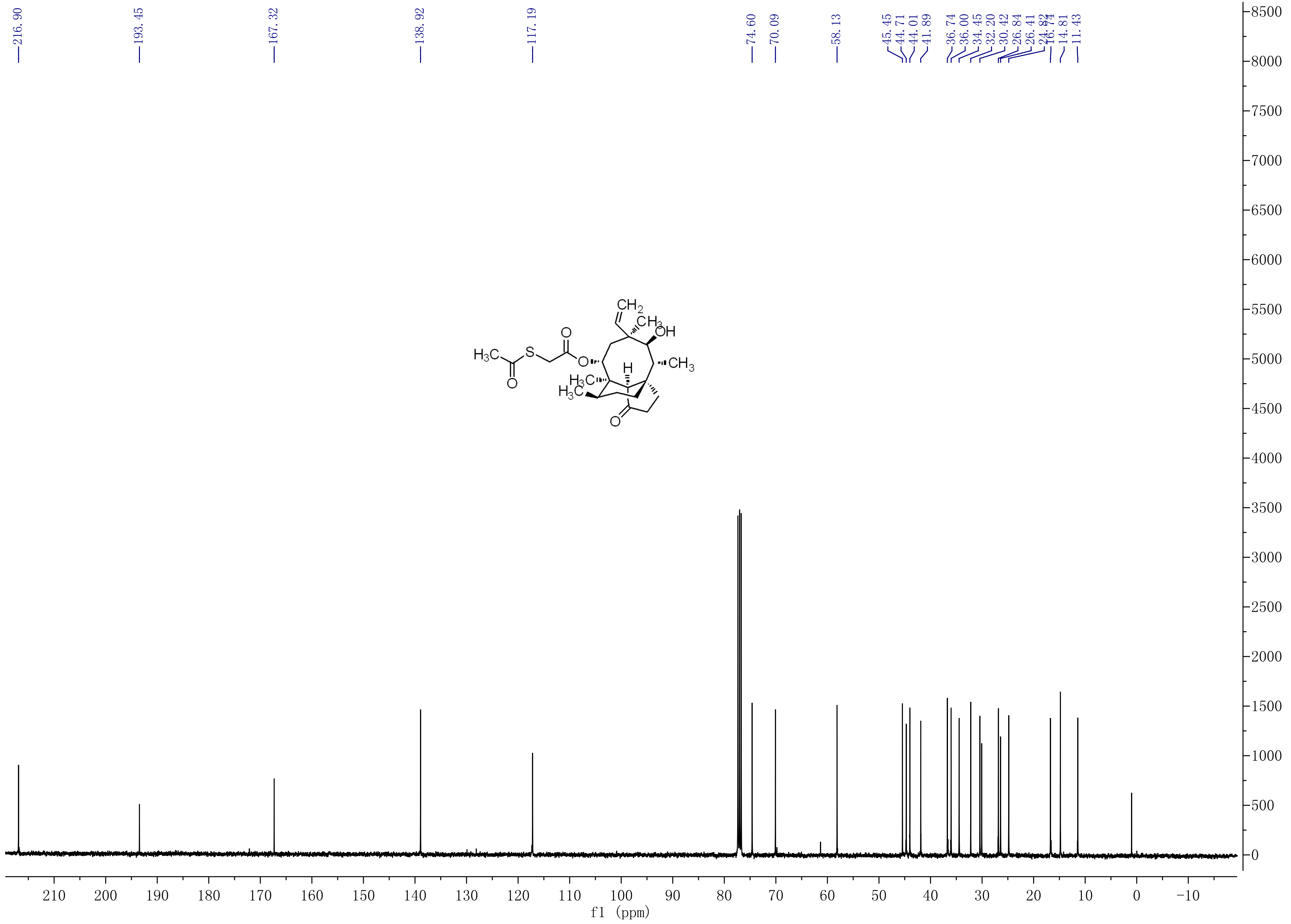
216.90
193.45
167.32
138.92
117.19
74.60
70.09
58.13
45.45
44.71
44.01
41.89
36.74
36.00
34.45
32.20
30.42
26.84
26.41
24.82
16.74
14.81
11.43
CH2
CH3
OH
CH3
O
H3C
S
O
H
H3C
H3C
O
O
f1 (ppm)

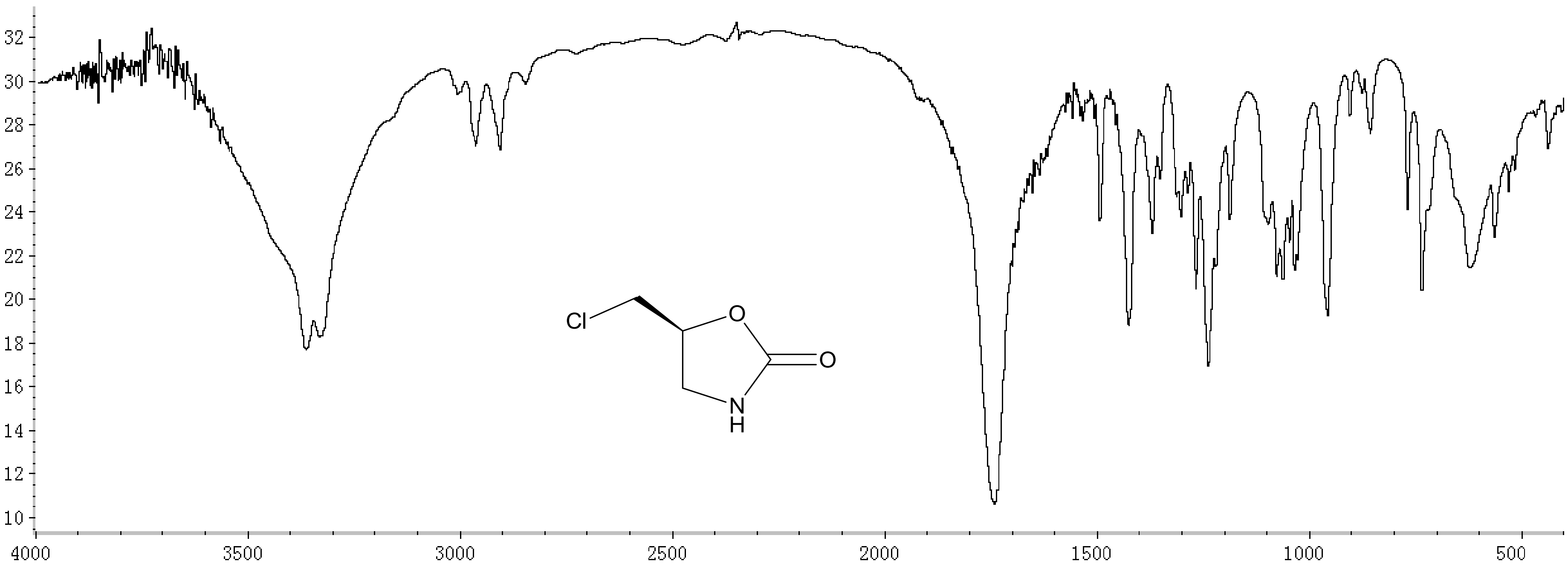

32
30
28
26
24
22
20
18
16
14
12
10
4000
3500
3000
2500
2000
1500
1000
500
Cl
O
O
N
H

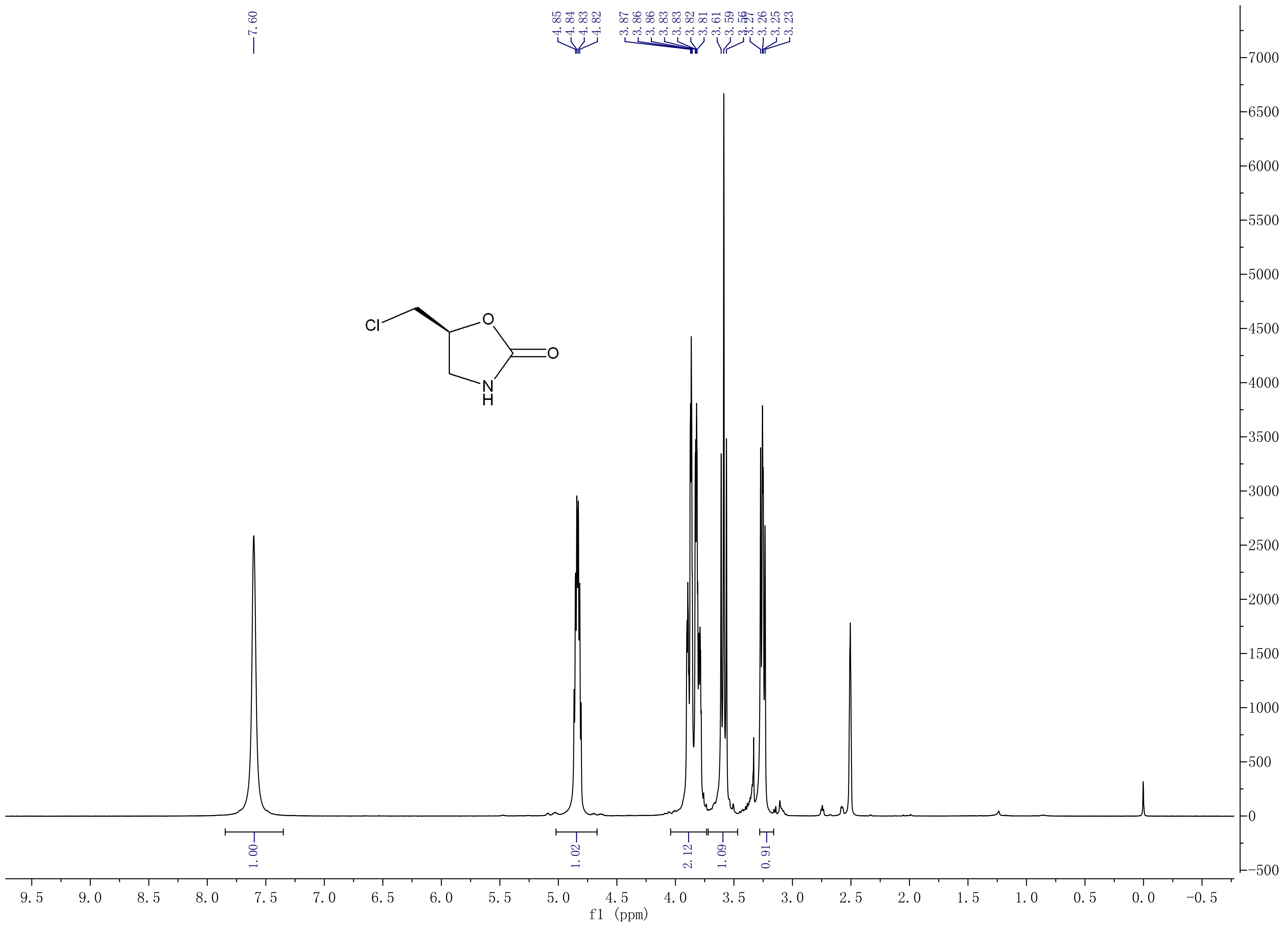
7.60
4.85
4.84
4.83
4.82
3.87
3.86
3.86
3.83
3.83
3.82
3.81
3.61
3.59
3.27
3.26
3.25
3.23
Cl
O
O
N
H
1.00
1.02
2.12
1.09
0.91
f1 (ppm)

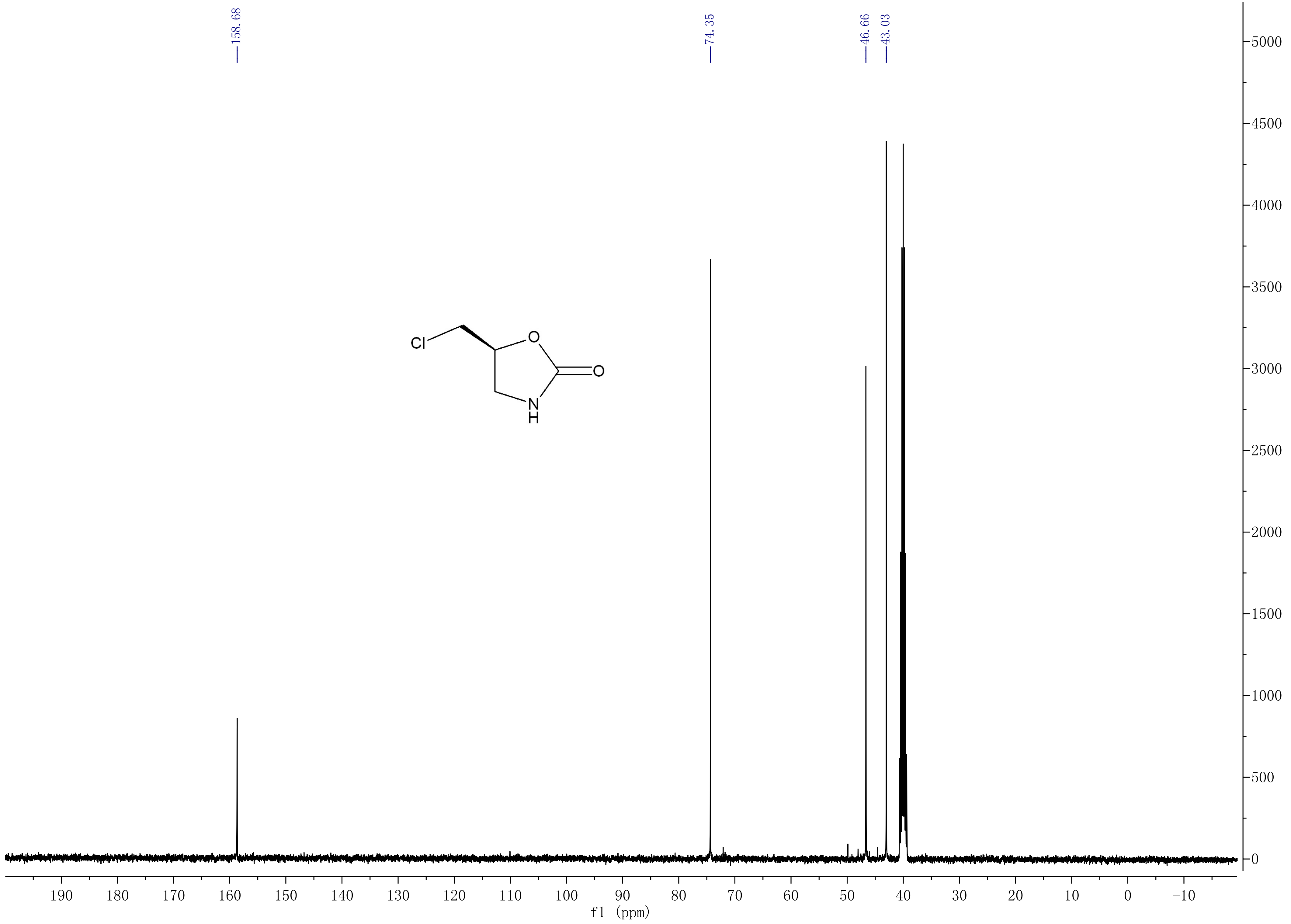
158.68
74.35
46.66
43.03
Cl
O
O
N
H
5000
4500
4000
3500
3000
2500
2000
1500
1000
500
0
190 180 170 160 150 140 130 120 110 100 90 80 70 60 50 40 30 20 10 0 -10
f1 (ppm)

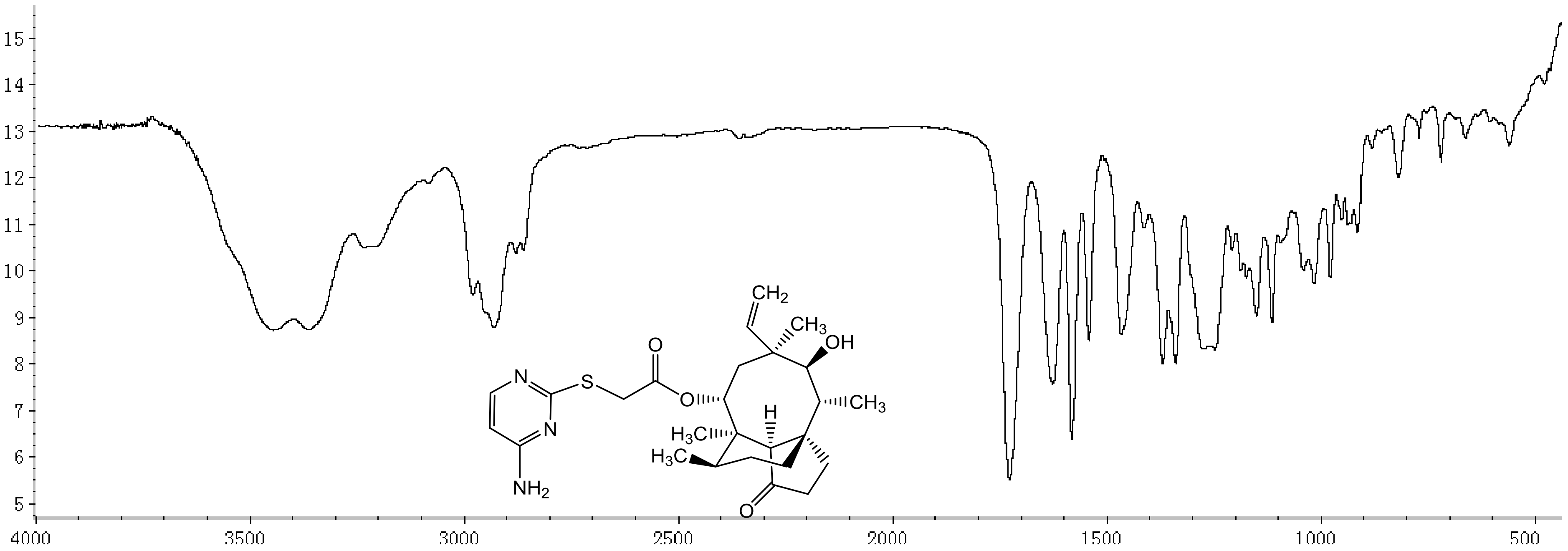
15
14
13
12
11
10
9
8
7
6
5
4000
3500
3000
2500
2000
1500
1000
500
CH2
CH3
OH
O
N
S
O
N
NH2
H3C
H3C
H
CH3
O

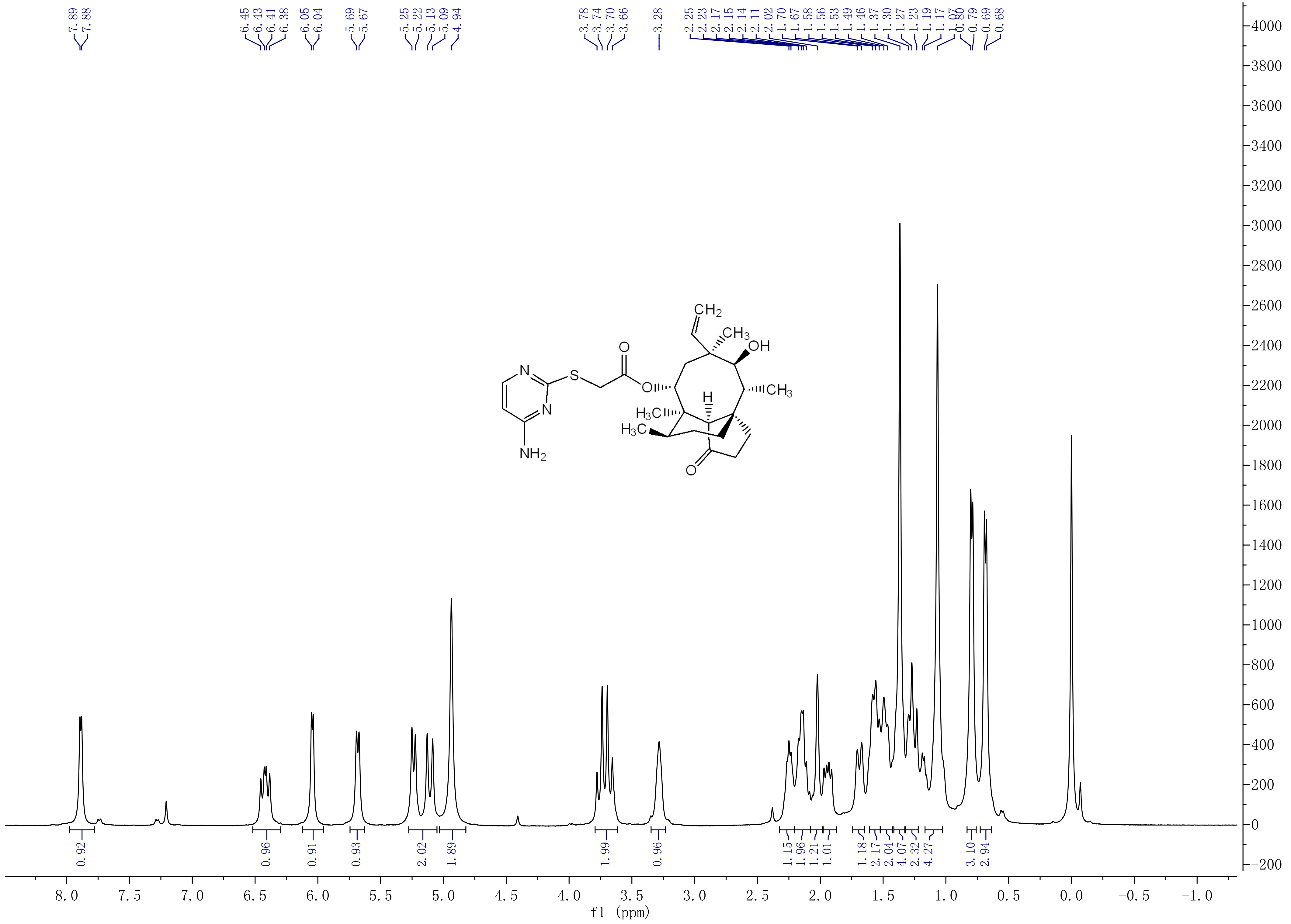

7.89 7.88 6.45 6.43 6.41 6.38 6.05 6.04 5.69 5.67 5.25 5.22 5.13 5.09 4.94 3.78 3.74 3.70 3.66 3.28 2.25 2.23 2.17 2.15 2.14 2.11 2.02 1.70 1.67 1.58 1.56 1.53 1.49 1.46 1.37 1.30 1.27 1.23 1.19 1.17 1.07 0.80 0.79 0.69 0.68
0.92 0.96 0.91 0.93 2.02 1.89 1.99 0.96 1.15 1.96 1.21 1.01 1.18 2.17 2.04 4.07 2.32 4.27 3.10 2.94
f1 (ppm)

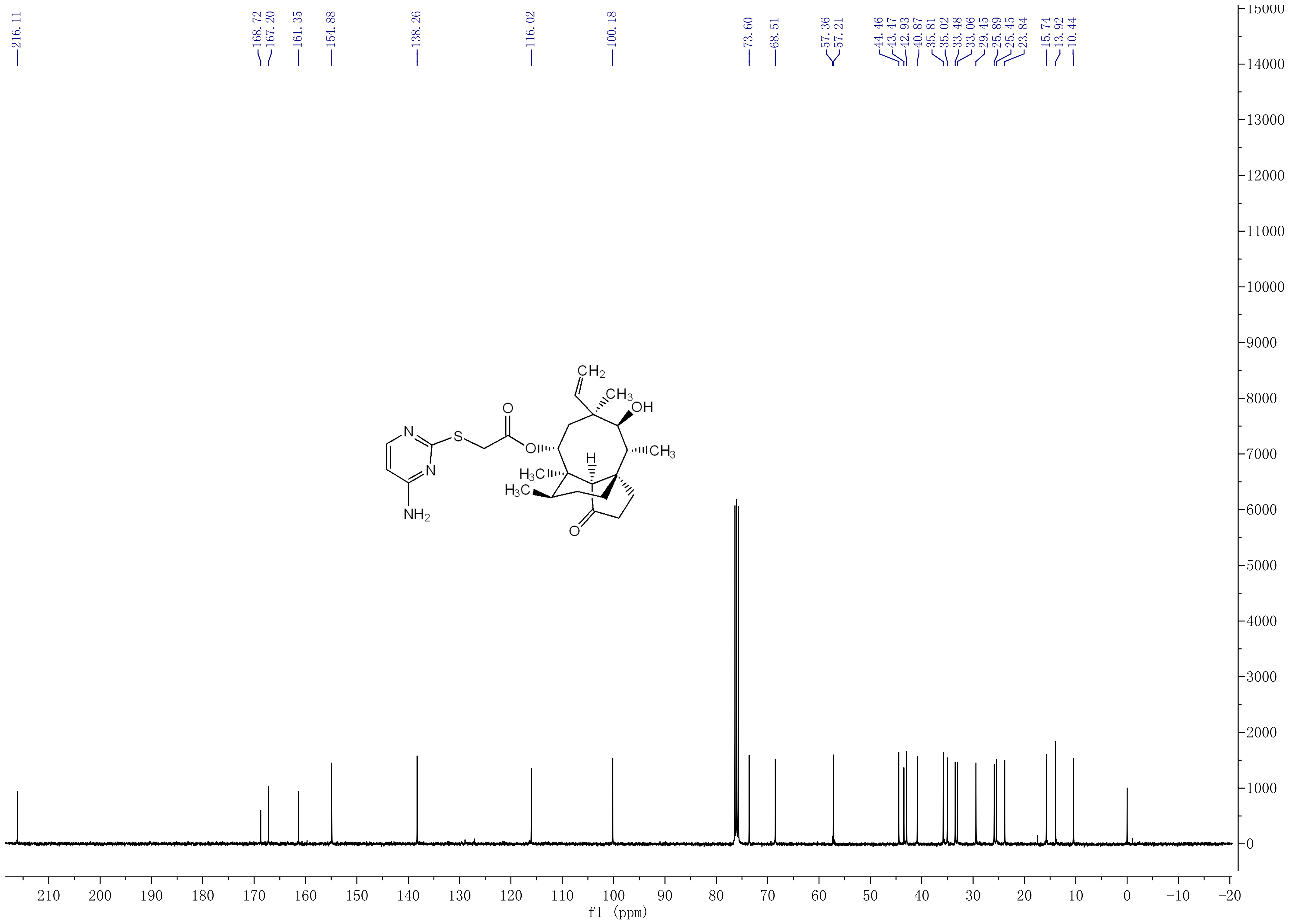
216.11
168.72
167.20
161.35
154.88
138.26
116.02
100.18
73.60
68.51
57.36
57.21
44.46
43.47
42.93
40.87
35.81
35.02
33.48
33.06
29.45
25.89
25.45
23.84
15.74
13.92
10.44
CH2
CH3
OH
O
N
S
O
N
CH3
H
H3C
H3C
NH2
O
210 200 190 180 170 160 150 140 130 120 110 100 90 80 70 60 50 40 30 20 10 0 −10 −20
f1 (ppm)
15000 14000 13000 12000 11000 10000 9000 8000 7000 6000 5000 4000 3000 2000 1000 0

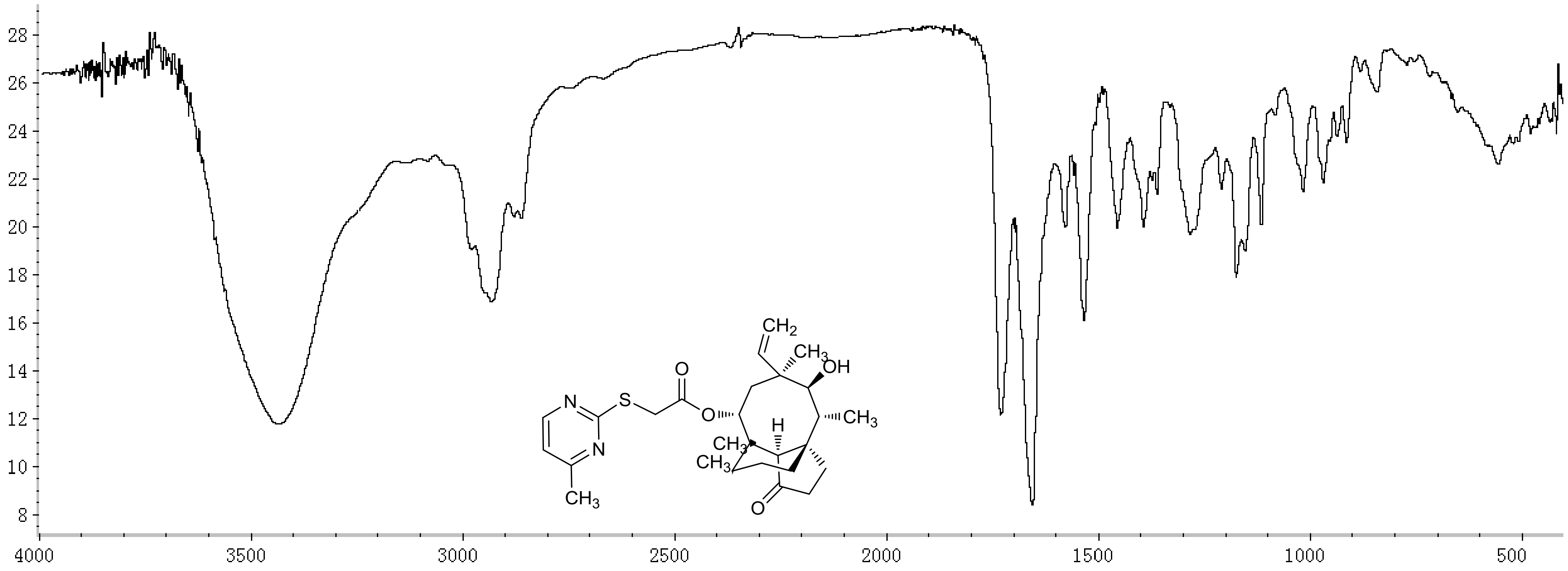
28
26
24
22
20
18
16
14
12
10
8
4000
3500
3000
2500
2000
1500
1000
500
CH2
CH3
OH
O
S
N
N
CH3
O
H
CH3
CH3
CH3
O

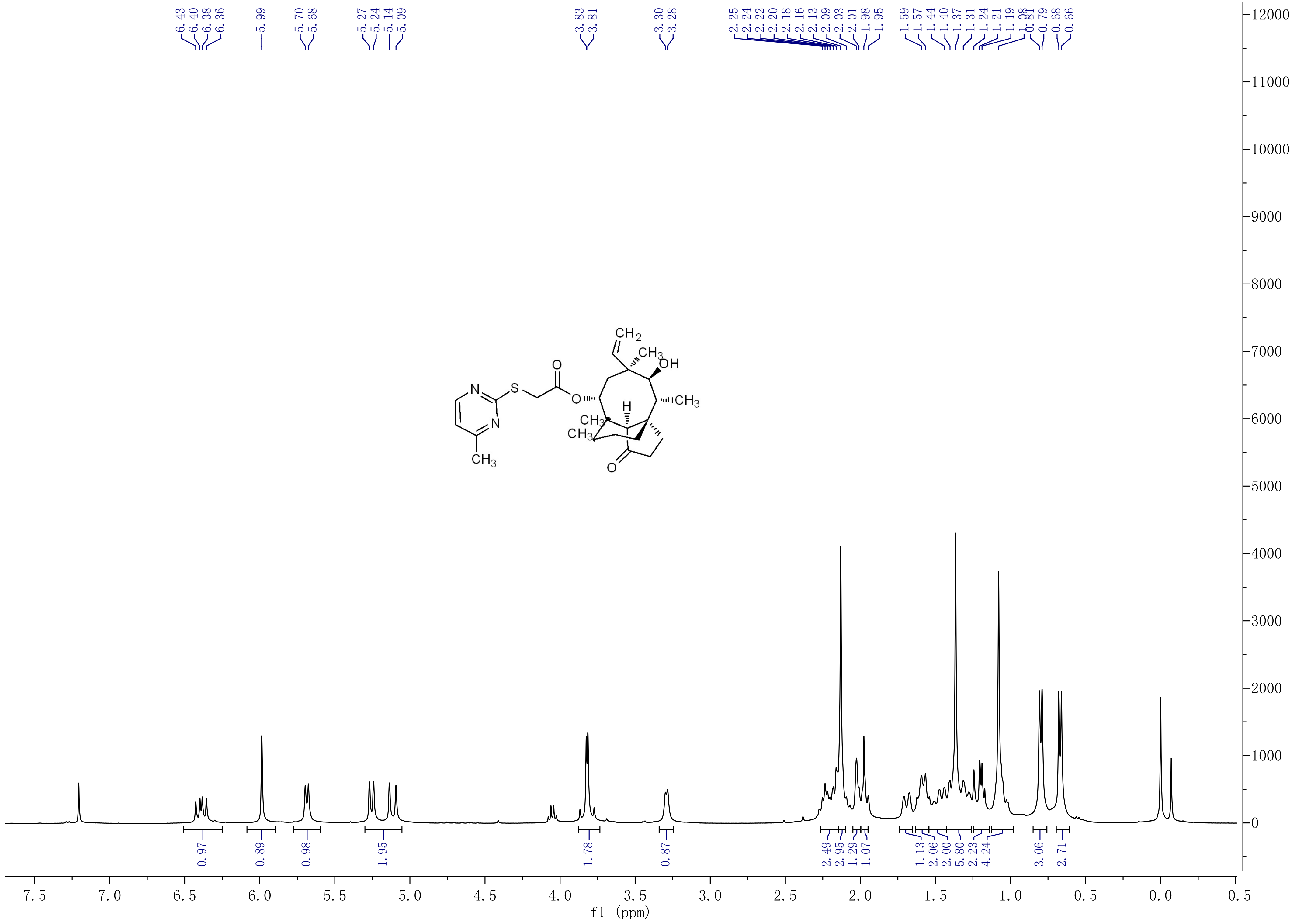
6.43 6.40 6.38 6.36
5.99
5.70 5.68
5.27 5.24 5.14 5.09
3.83 3.81
3.30 3.28
2.25 2.24 2.22 2.20 2.18 2.16 2.13 2.09 2.03 2.01 1.98 1.95
1.59 1.57 1.44 1.40 1.37 1.31 1.24 1.21 1.19 1.08 0.81 0.79 0.68 0.66
0.97
0.89
0.98
1.95
1.78
0.87
2.49 2.95 1.29 1.07
1.13 2.06 2.00 5.80 2.23 4.24
3.06 2.71
CH2
CH3
OH
O
N
S
N
O
H
CH3
CH3
CH3
O
CH3
12000
11000
10000
9000
8000
7000
6000
5000
4000
3000
2000
1000
0
7.5 7.0 6.5 6.0 5.5 5.0 4.5 4.0 3.5 3.0 2.5 2.0 1.5 1.0 0.5 0.0 -0.5
f1 (ppm)

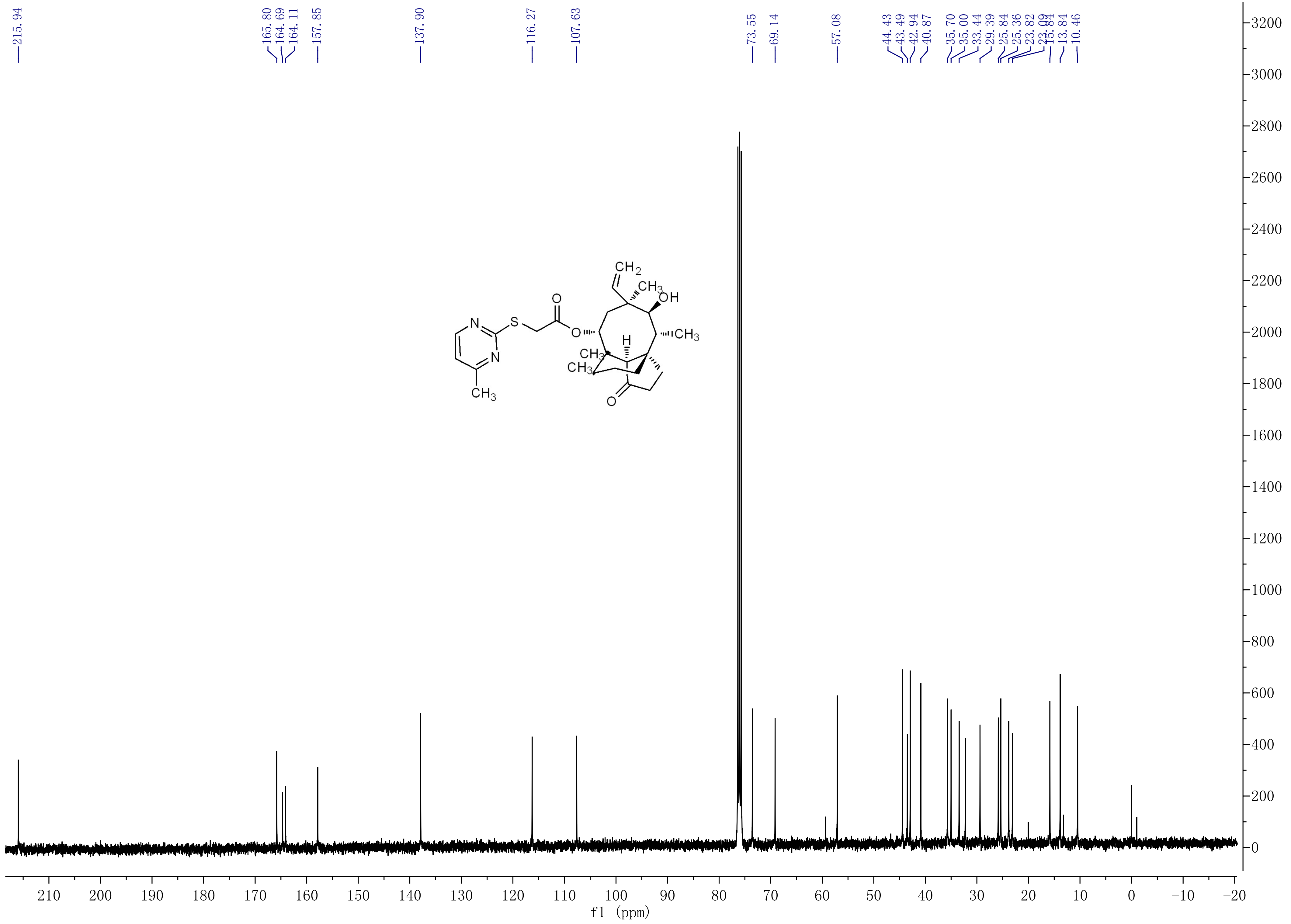

215.94
165.80
164.69
164.11
157.85
137.90
116.27
107.63
73.55
69.14
57.08
44.43
43.49
42.94
40.87
35.70
35.00
33.44
29.39
25.84
25.36
23.82
13.84
10.46
f1 (ppm)

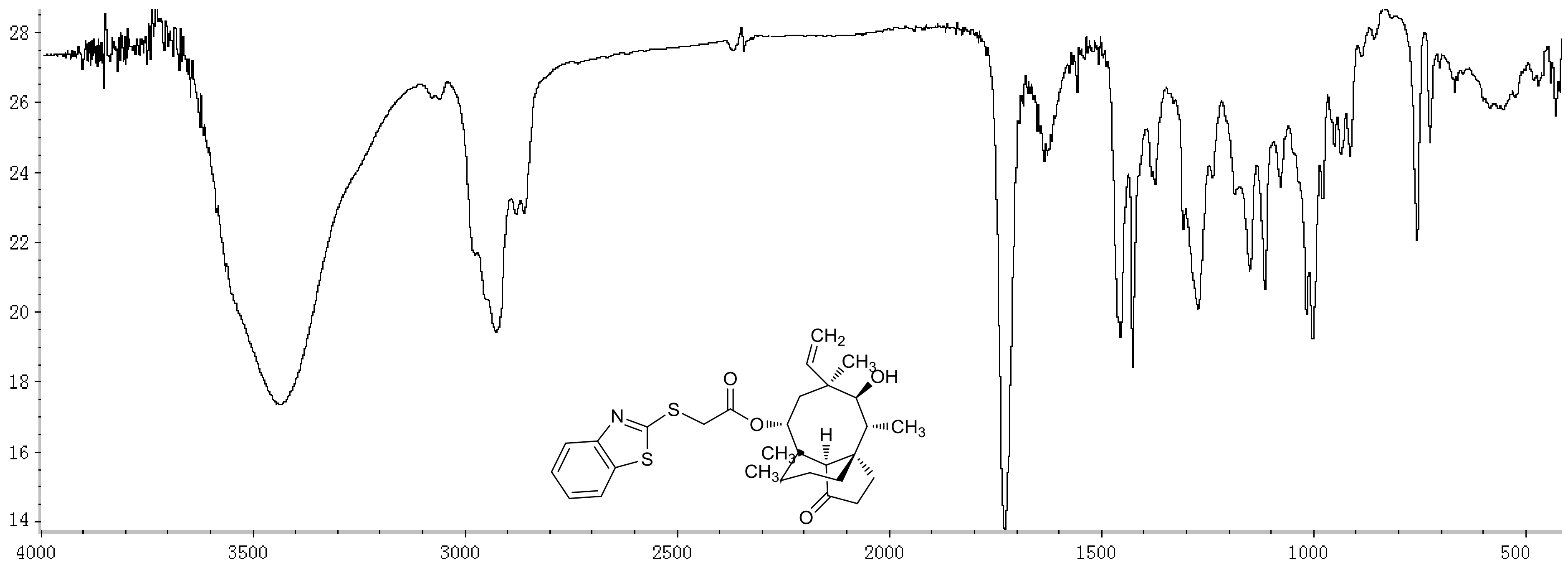

28
26
24
22
20
18
16
14
4000
3500
3000
2500
2000
1500
1000
500
CH2
CH3
OH
O
N
S
O
S
H
CH3
CH3
CH3
O

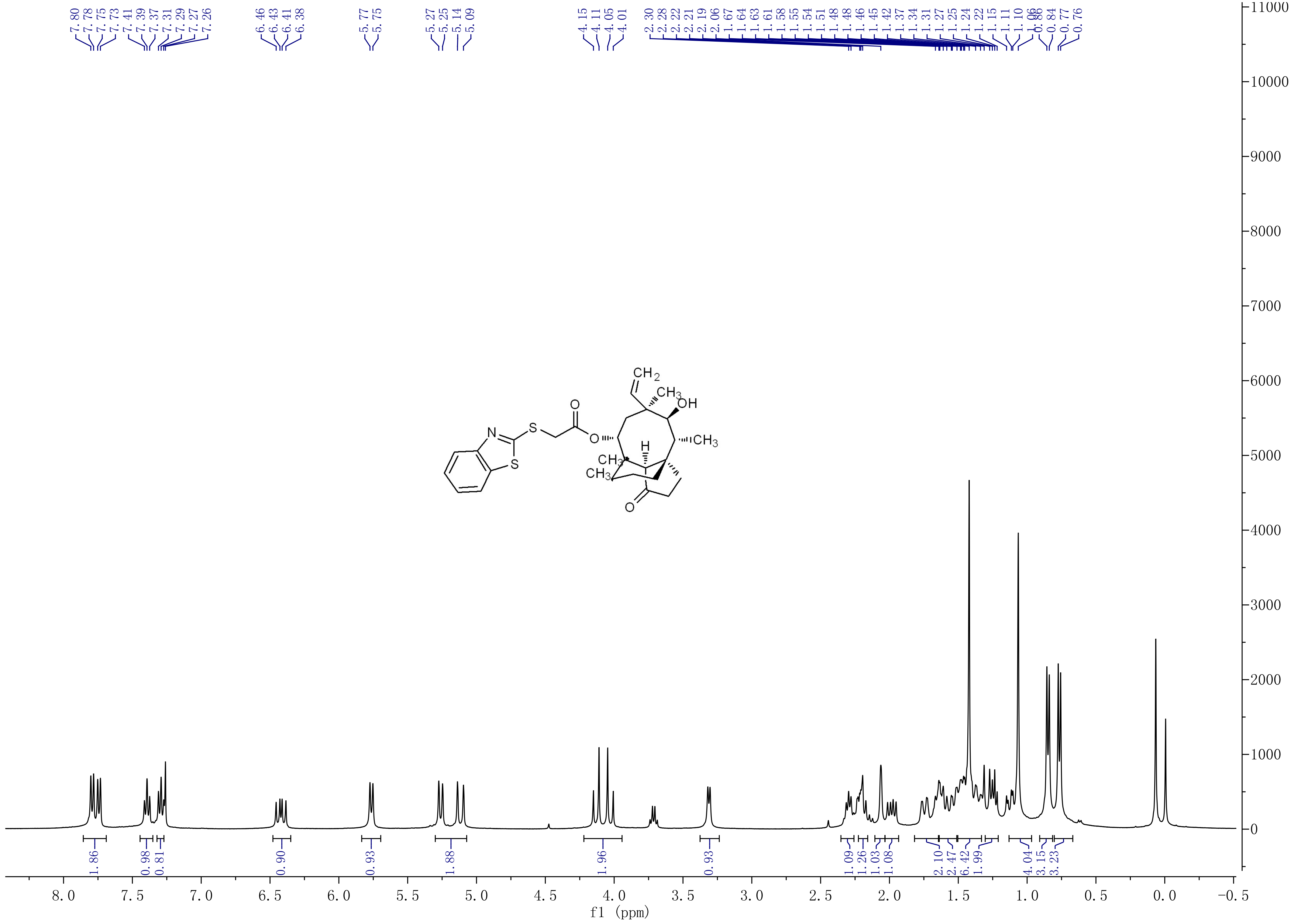

7.80
7.78
7.75
7.73
7.41
7.39
7.37
7.31
7.29
7.27
7.26
6.46
6.43
6.41
6.38
5.77
5.75
5.27
5.25
5.14
5.09
4.15
4.11
4.05
4.01
2.30
2.28
2.22
2.21
2.19
2.06
1.67
1.64
1.63
1.61
1.58
1.55
1.54
1.51
1.48
1.48
1.46
1.45
1.42
1.37
1.34
1.31
1.27
1.25
1.24
1.22
1.15
1.11
1.10
1.06
0.86
0.84
0.77
0.76
11000
10000
9000
8000
7000
6000
5000
4000
3000
2000
1000
0
CH2
CH3
OH
O
N
S
O
CH3
H
CH3
CH3
O
1.86
0.98
0.81
0.90
0.93
1.88
1.96
0.93
1.09
1.26
1.03
1.08
2.10
2.47
6.42
1.99
4.04
3.15
3.23
8.0
7.5
7.0
6.5
6.0
5.5
5.0
4.5
4.0
3.5
3.0
2.5
2.0
1.5
1.0
0.5
0.0
-0.5
f1 (ppm)

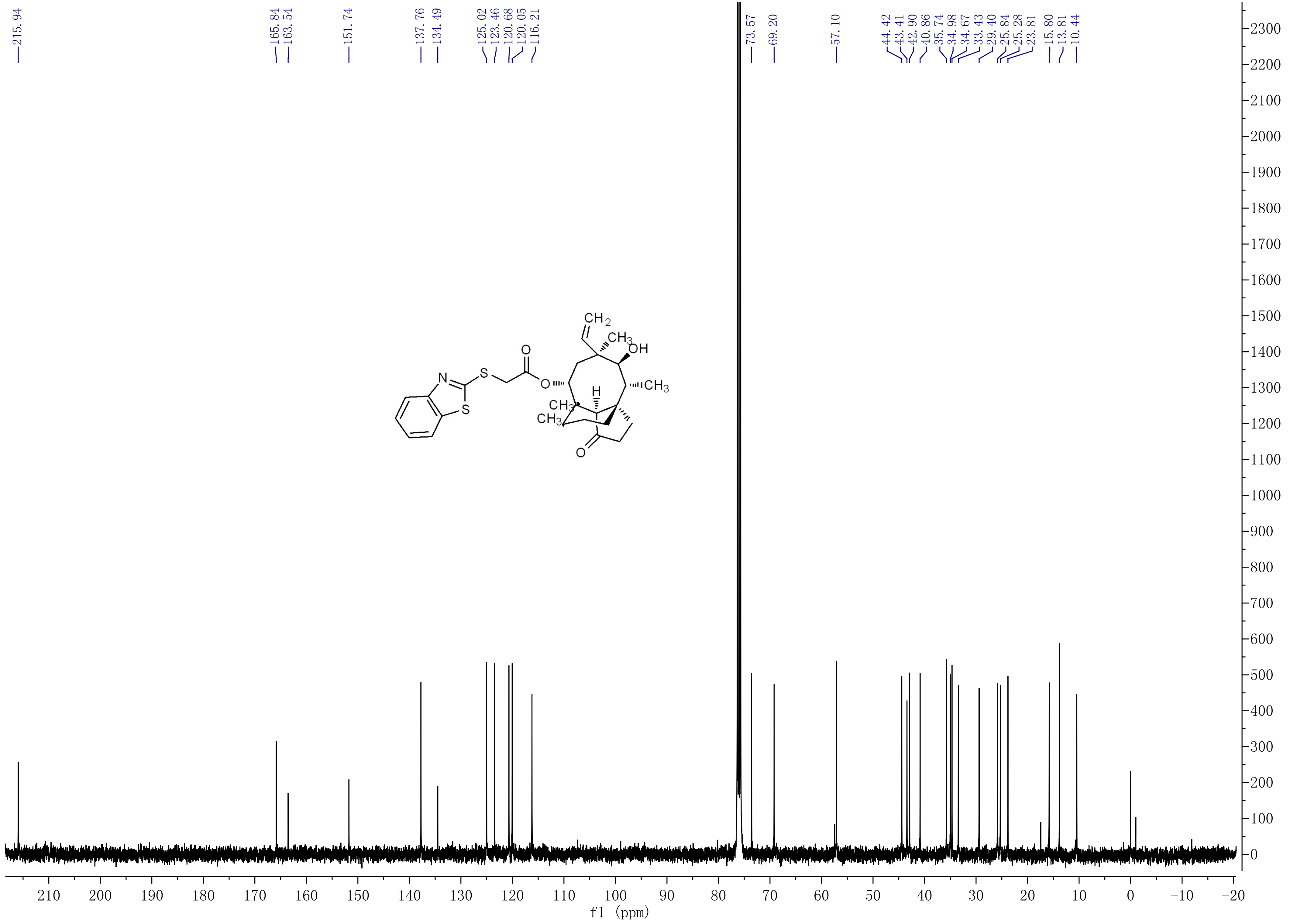
215.94
165.84
163.54
151.74
137.76
134.49
125.02
123.46
120.68
120.05
116.21
73.57
69.20
57.10
44.42
43.41
42.90
40.86
35.74
34.98
34.67
33.43
29.40
25.84
25.28
23.81
15.80
13.81
10.44
f1 (ppm)

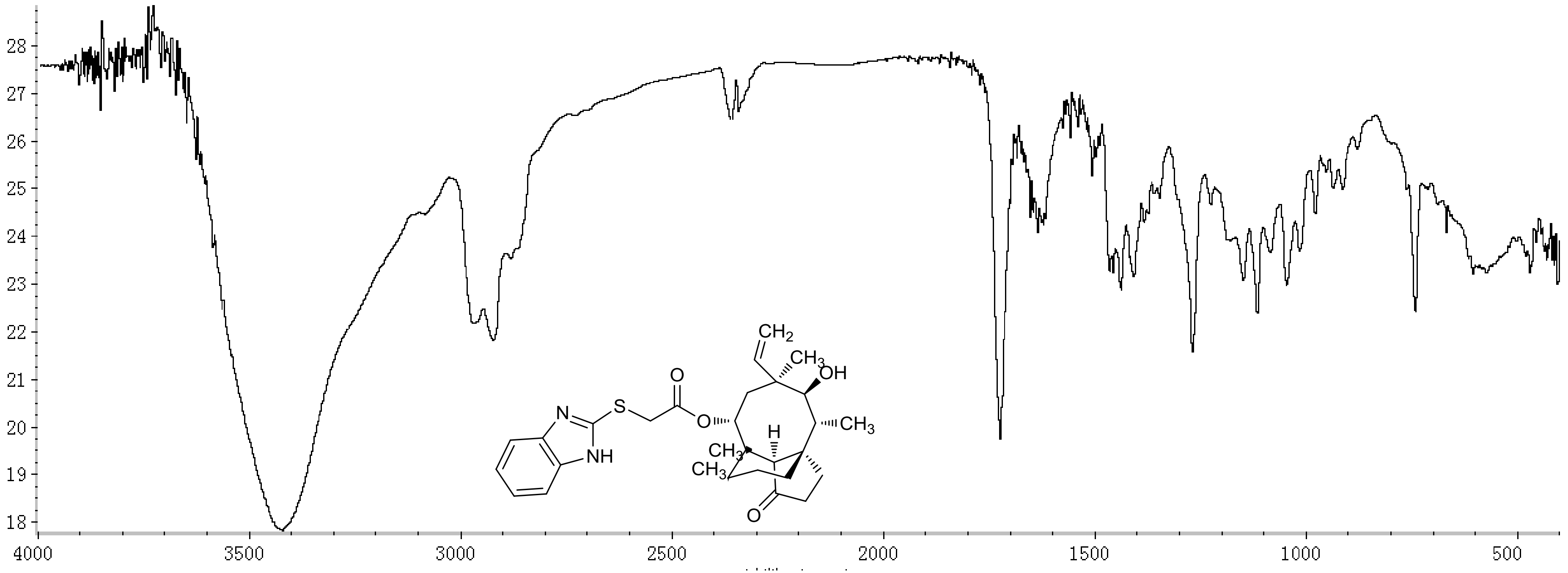
28
27
26
25
24
23
22
21
20
19
18
4000
3500
3000
2500
2000
1500
1000
500
CH2
CH3
OH
O
N
S
NH
O
H
CH3
CH3
CH3
O

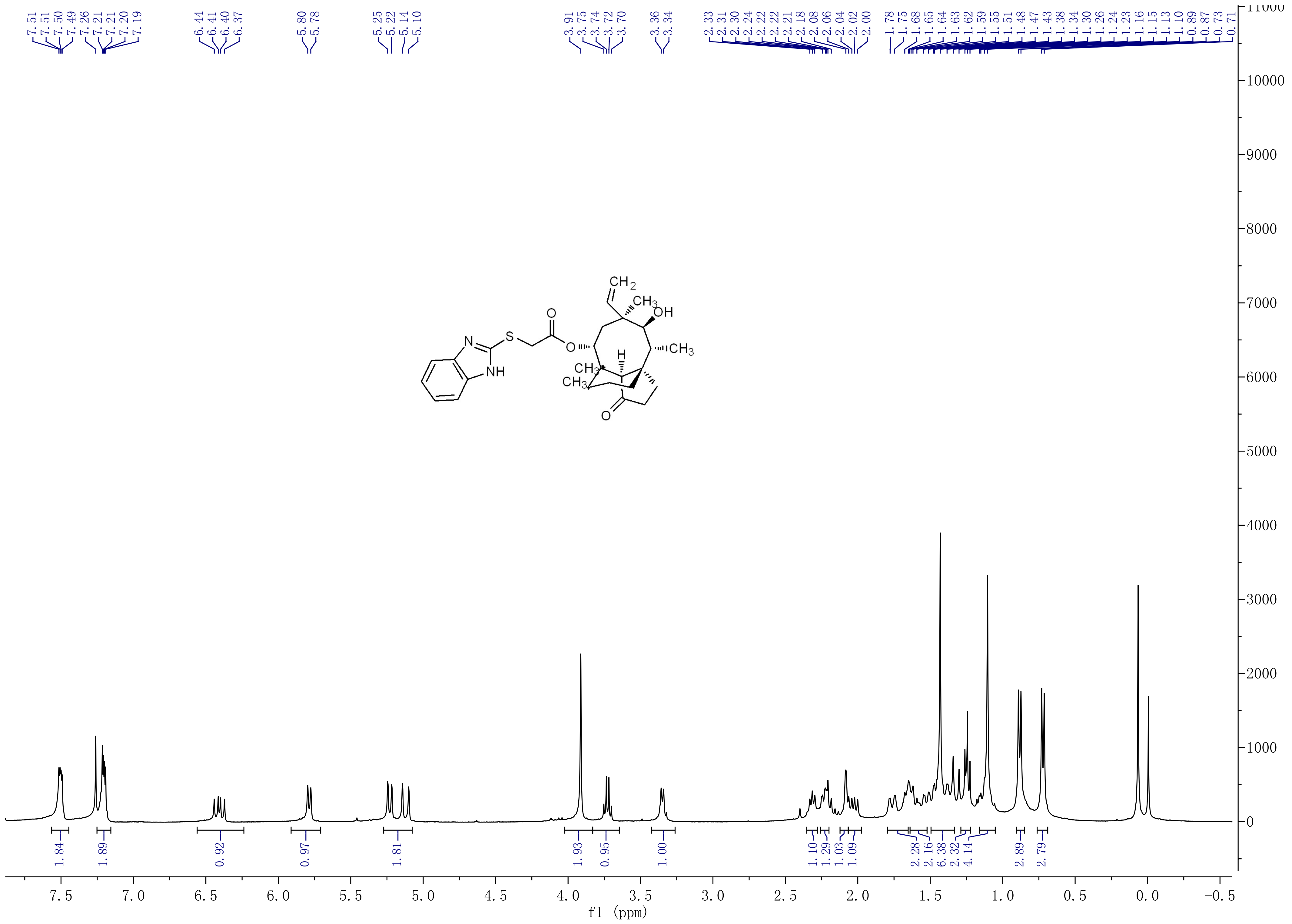

7.51 7.51 7.50 7.49 7.26 7.21 7.21 7.20 7.19
6.44 6.41 6.40 6.37
5.80 5.78
5.25 5.22 5.14 5.10
3.91 3.75 3.74 3.72 3.70
3.36 3.34
2.33 2.31 2.30 2.24 2.22 2.22 2.21 2.18 2.08 2.06 2.04 2.02 2.00
1.78 1.75 1.68 1.65 1.64 1.63 1.62 1.59 1.55 1.51 1.48 1.47 1.43 1.38 1.34 1.30 1.26 1.24 1.23 1.16 1.15 1.13 1.10 0.89 0.87 0.73 0.71
1.84 1.89 0.92 0.97 1.81 1.93 0.95 1.00 1.10 1.29 1.03 1.09 2.28 2.16 6.38 2.32 4.14 2.89 2.79
CH2
CH3
OH
O
N
S
NH
O
CH3
H
CH3
CH3
O
f1 (ppm)

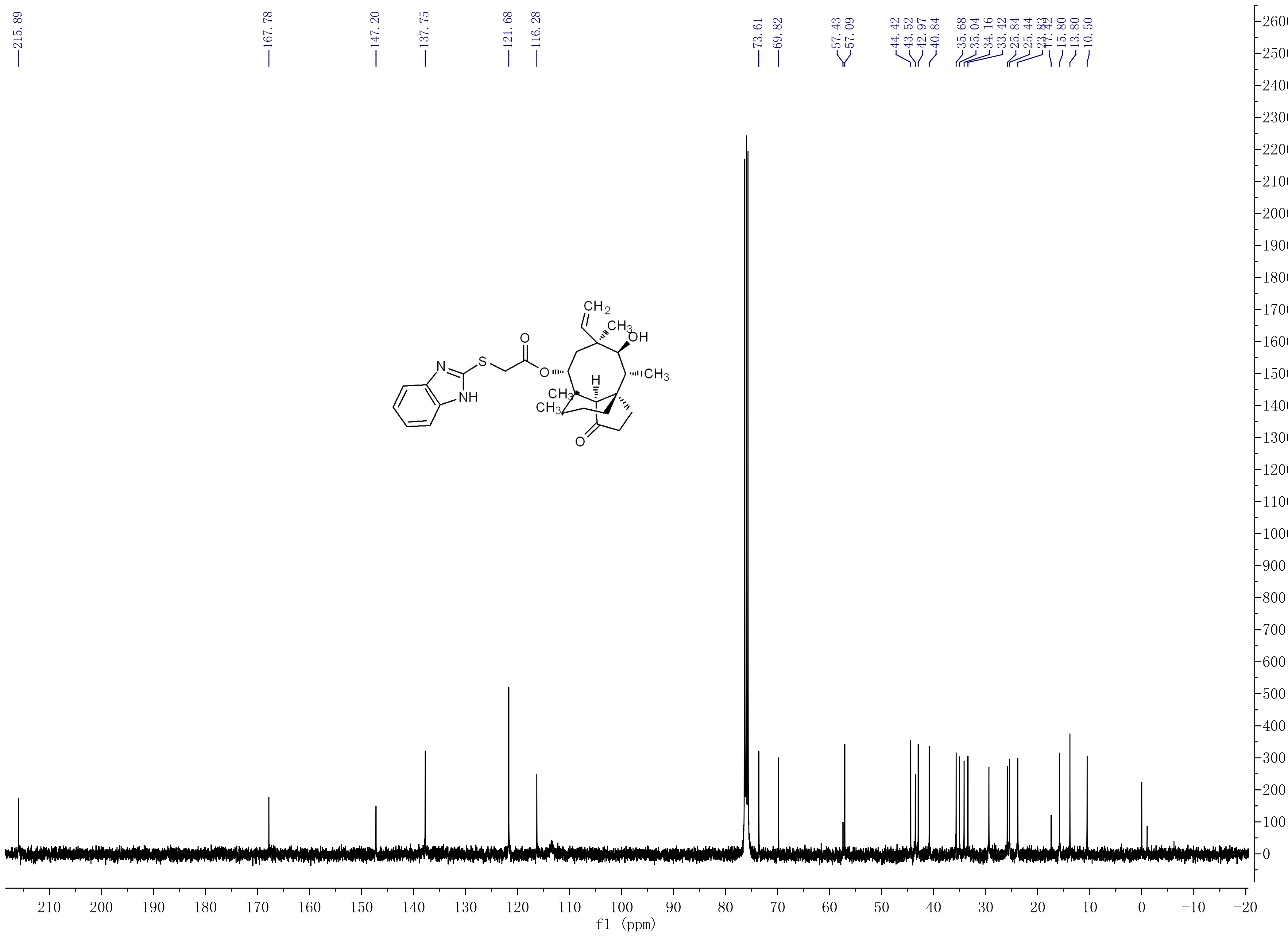

215.89
167.78
147.20
137.75
121.68
116.28
73.61
69.82
57.43
57.09
44.42
43.52
42.97
40.84
35.68
35.04
34.16
33.42
25.84
25.44
15.80
13.80
10.50
CH2
CH3
OH
O
N
S
NH
O
H
CH3
CH3
CH3
O
f1 (ppm)

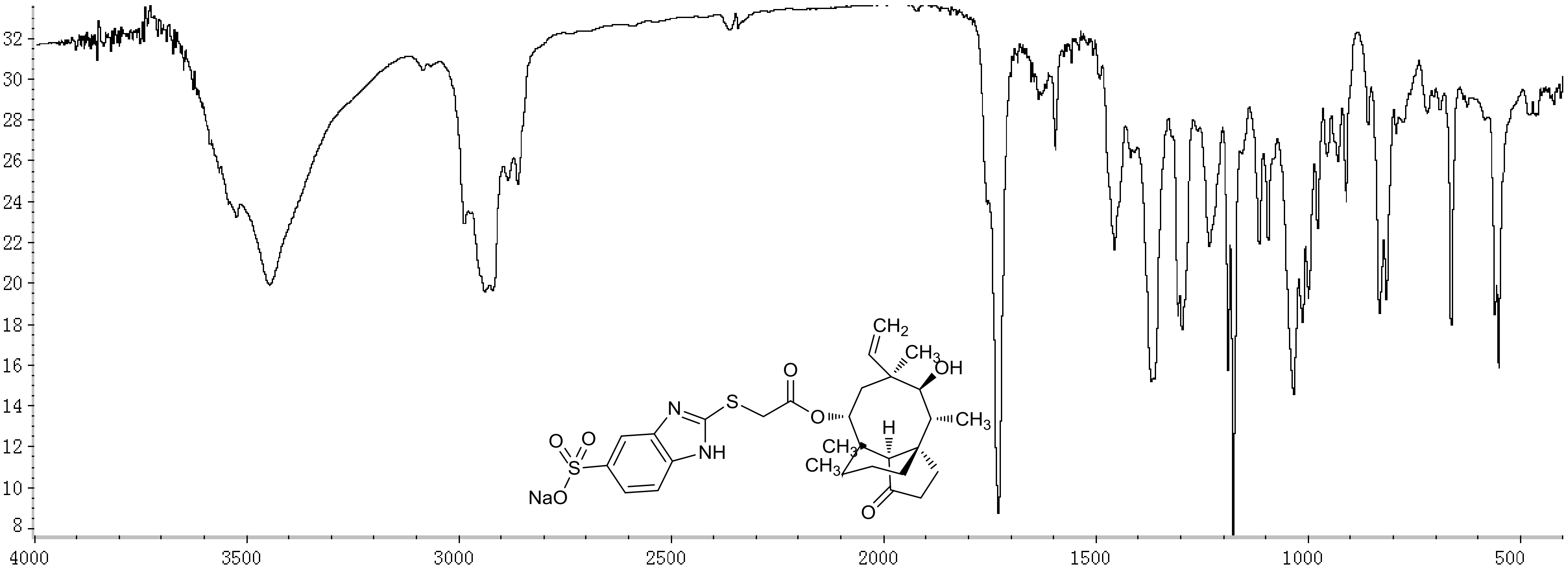

32
30
28
26
24
22
20
18
16
14
12
10
8
4000
3500
3000
2500
2000
1500
1000
500
CH2
CH3
OH
O
N
S
O
CH3
H
O
O
NH
CH3
S
CH3
NaO
O

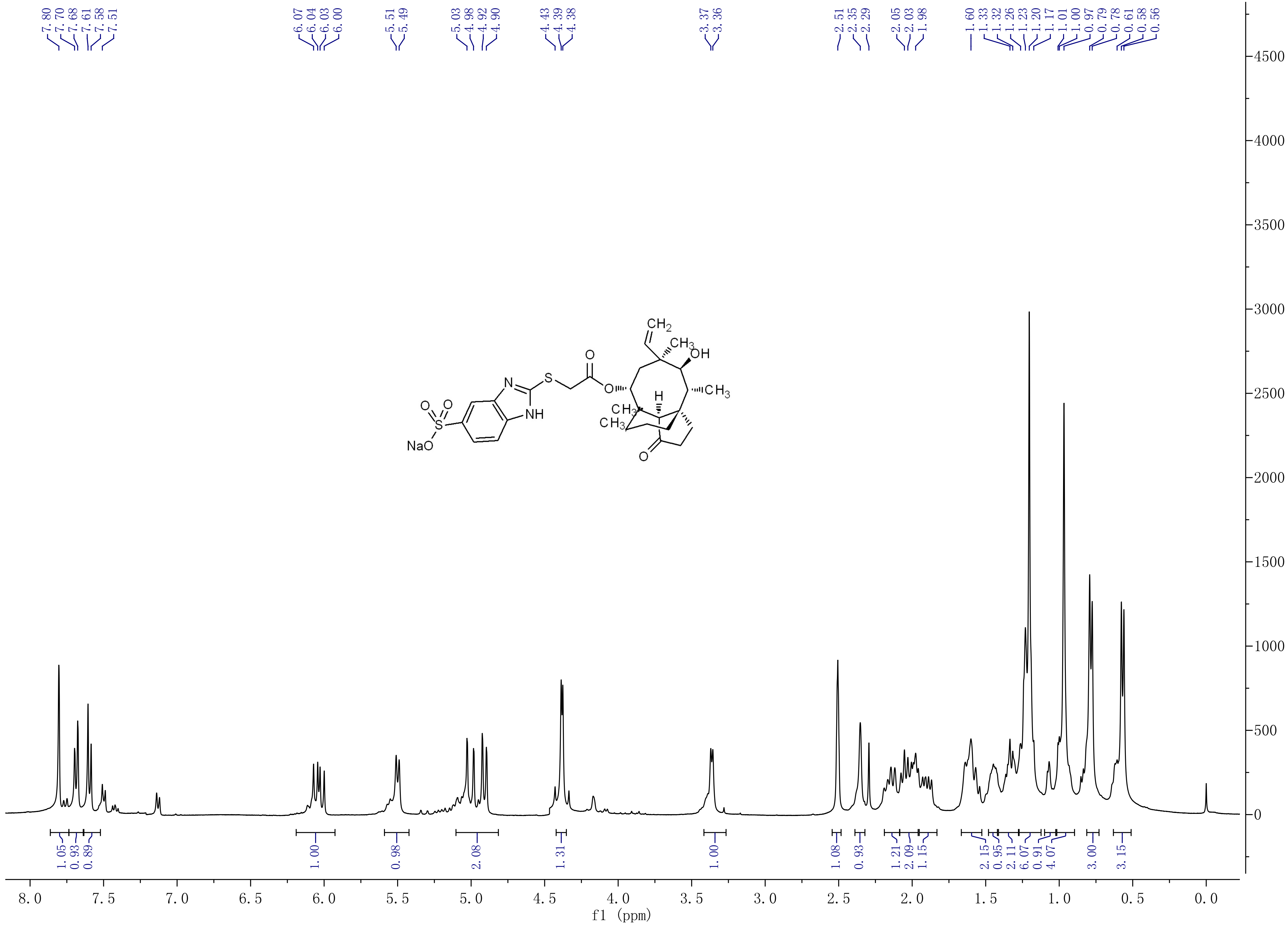
7.80 7.70 7.68 7.61 7.58 7.51
6.07 6.04 6.03 6.00
5.51 5.49
5.03 4.98 4.92 4.90
4.43 4.39 4.38
3.37 3.36
2.51 2.35 2.29
2.05 2.03 1.98
1.60 1.33 1.32 1.26 1.23 1.20 1.17 1.01 1.00 0.97 0.79 0.78 0.61 0.58 0.56
1.05 0.93 0.89 1.00 0.98 2.08 1.31 1.00 1.08 0.93 1.21 2.09 1.15 2.15 0.95 2.11 6.07 0.91 4.07 3.00 3.15
8.0 7.5 7.0 6.5 6.0 5.5 5.0 4.5 4.0 3.5 3.0 2.5 2.0 1.5 1.0 0.5 0.0
f1 (ppm)
4500 4000 3500 3000 2500 2000 1500 1000 500 0
NaO
S
O
O
N
NH
S
O
O
CH2
CH3
OH
CH3
H
CH3
CH3
O

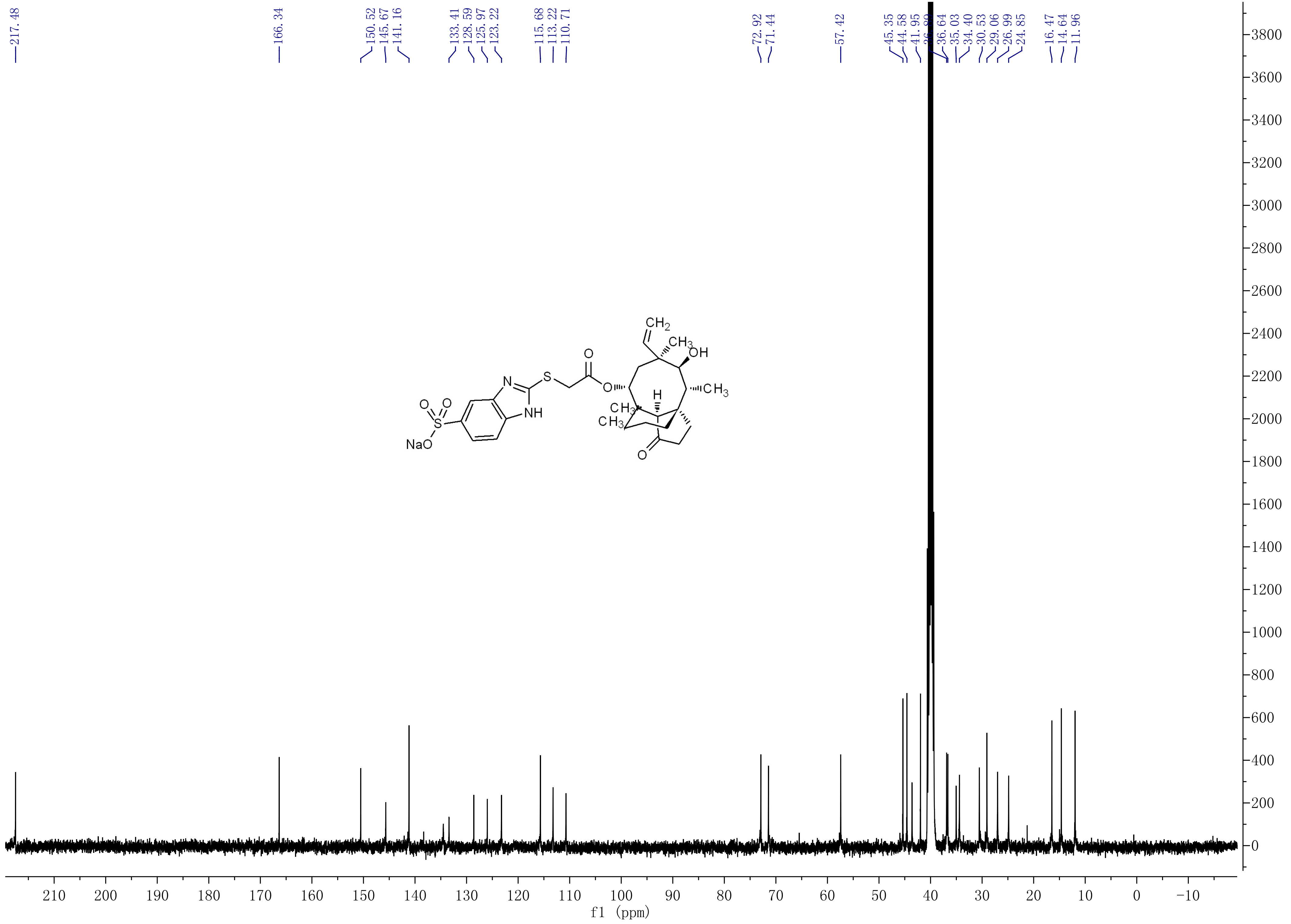

217.48
166.34
150.52
145.67
141.16
133.41
128.59
125.97
123.22
115.68
113.22
110.71
72.92
71.44
57.42
45.35
44.58
41.95
36.64
35.03
34.40
30.53
29.06
26.99
24.85
16.47
14.64
11.96
CH2
CH3
OH
O
N
S
NH
O
O
S
NaO
H
CH3
CH3
CH3
O
3800
3600
3400
3200
3000
2800
2600
2400
2200
2000
1800
1600
1400
1200
1000
800
600
400
200
0
210 200 190 180 170 160 150 140 130 120 110 100 90 80 70 60 50 40 30 20 10 0 -10
f1 (ppm)

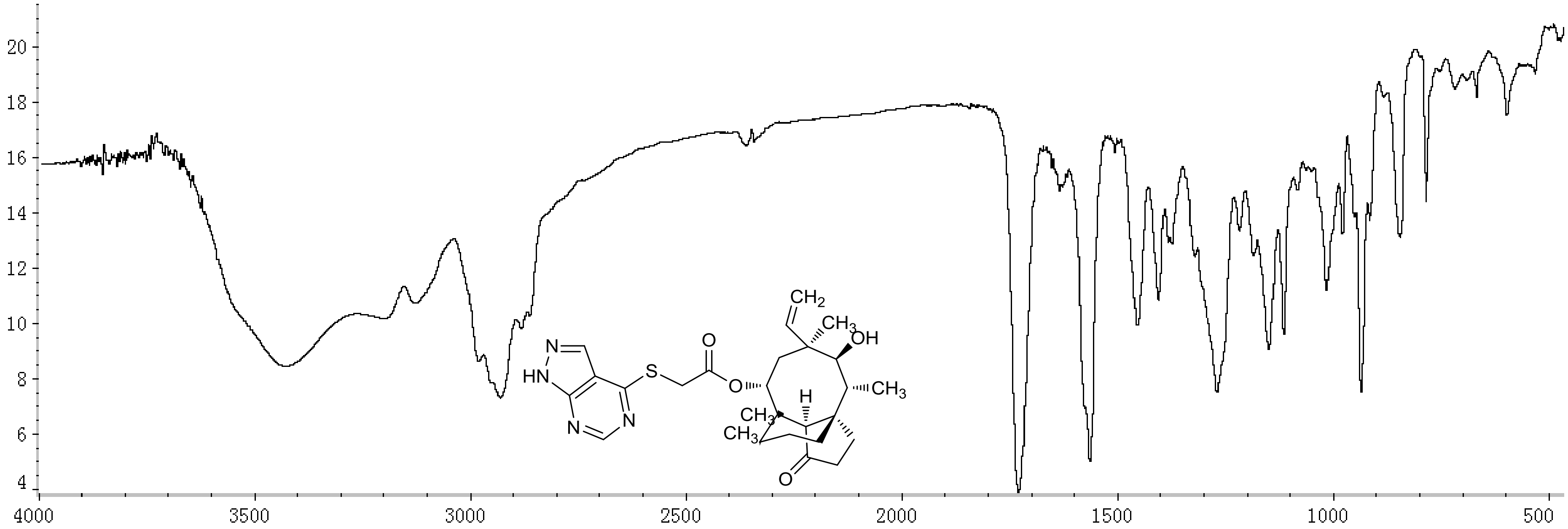

20
18
16
14
12
10
8
6
4
4000
3500
3000
2500
2000
1500
1000
500

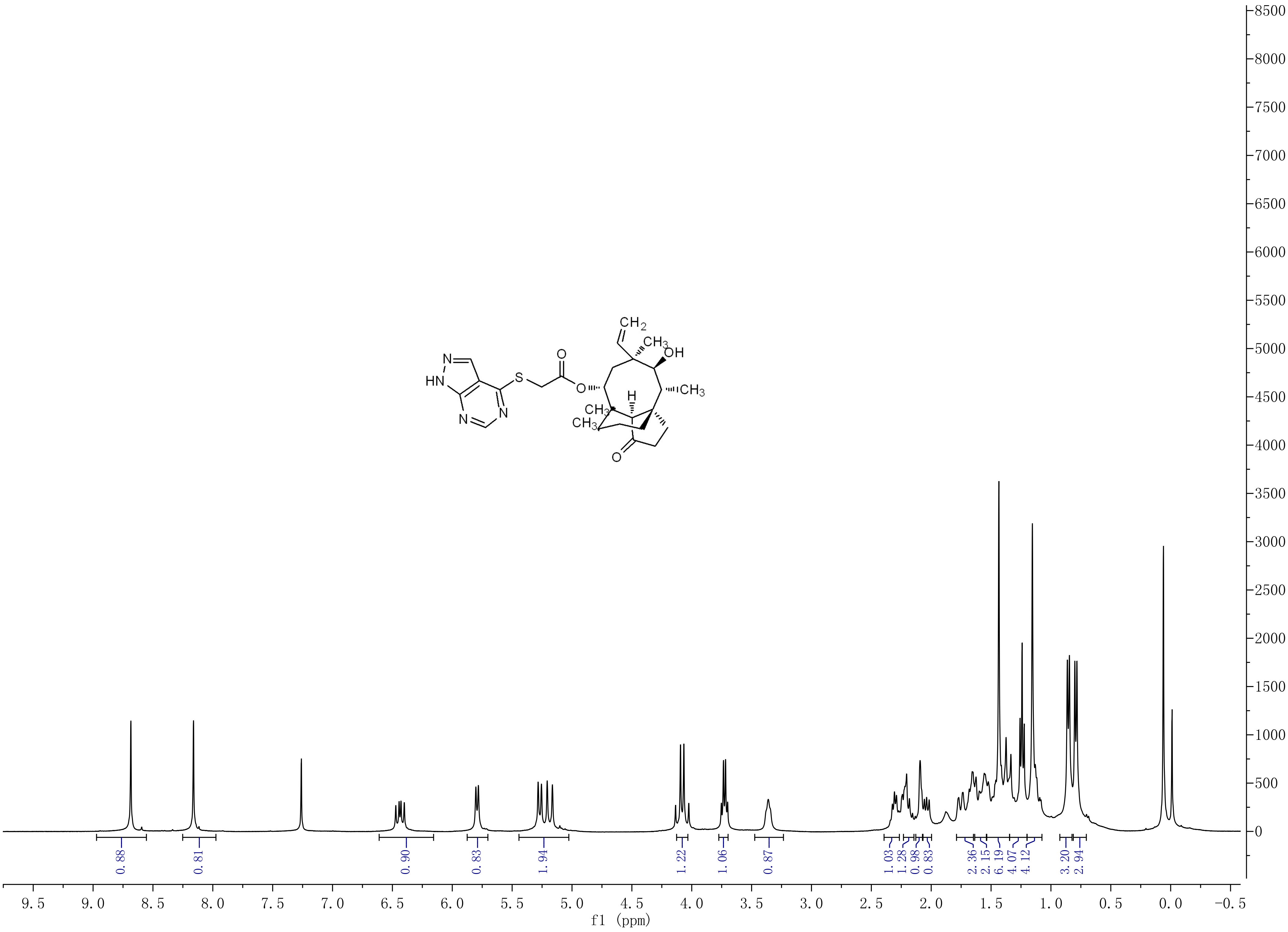

CH2
CH3
OH
CH3
H
CH3
CH3
O
N
HN
S
O
N
N
0.88
0.81
0.90
0.83
1.94
1.22
1.06
0.87
1.03
1.28
0.98
0.83
2.36
2.15
6.19
4.07
4.12
3.20
2.94
9.5
9.0
8.5
8.0
7.5
7.0
6.5
6.0
5.5
5.0
4.5
4.0
3.5
3.0
2.5
2.0
1.5
1.0
0.5
0.0
-0.5
f1 (ppm)
8500
8000
7500
7000
6500
6000
5500
5000
4500
4000
3500
3000
2500
2000
1500
1000
500
0

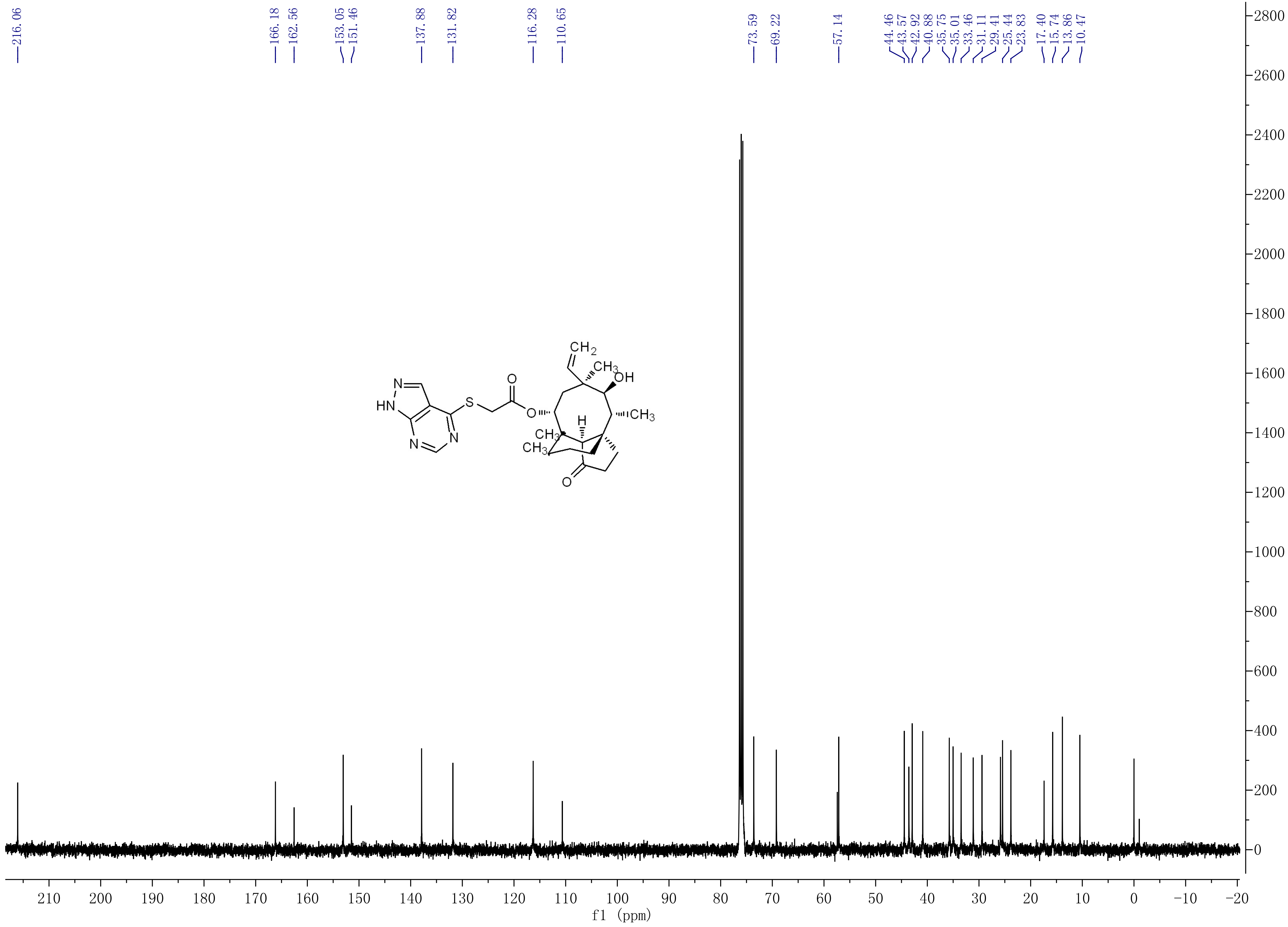
216.06
166.18
162.56
153.05
151.46
137.88
131.82
116.28
110.65
73.59
69.22
57.14
44.46
43.57
42.92
40.88
35.75
35.01
33.46
31.11
29.41
25.44
23.83
17.40
15.74
13.86
10.47
CH2
CH3
OH
O
N
HN
S
CH3
H
CH3
CH3
N
N
O
2800
2600
2400
2200
2000
1800
1600
1400
1200
1000
800
600
400
200
0
210 200 190 180 170 160 150 140 130 120 110 100 90 80 70 60 50 40 30 20 10 0 −10 −20
f1 (ppm)

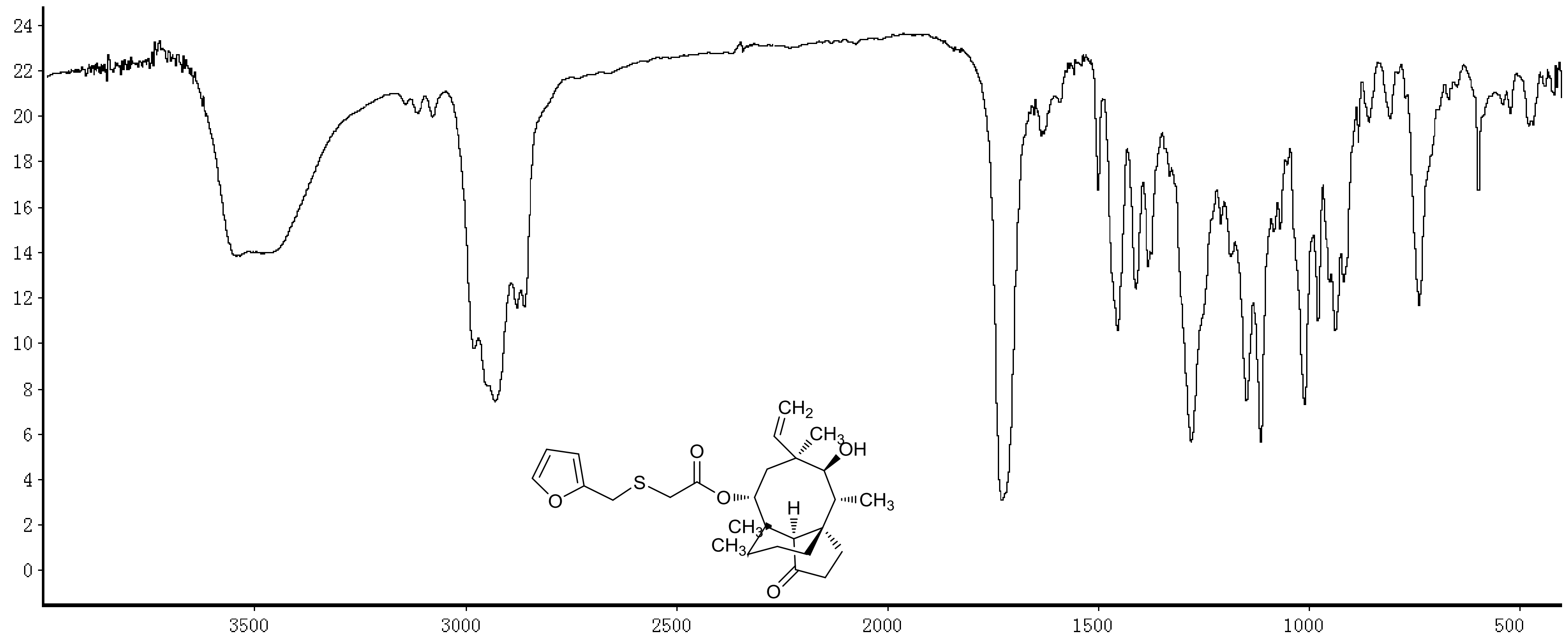

24
22
20
18
16
14
12
10
8
6
4
2
0
3500
3000
2500
2000
1500
1000
500
CH2
CH3
OH
O
S
O
O
H
CH3
CH3
CH3
O

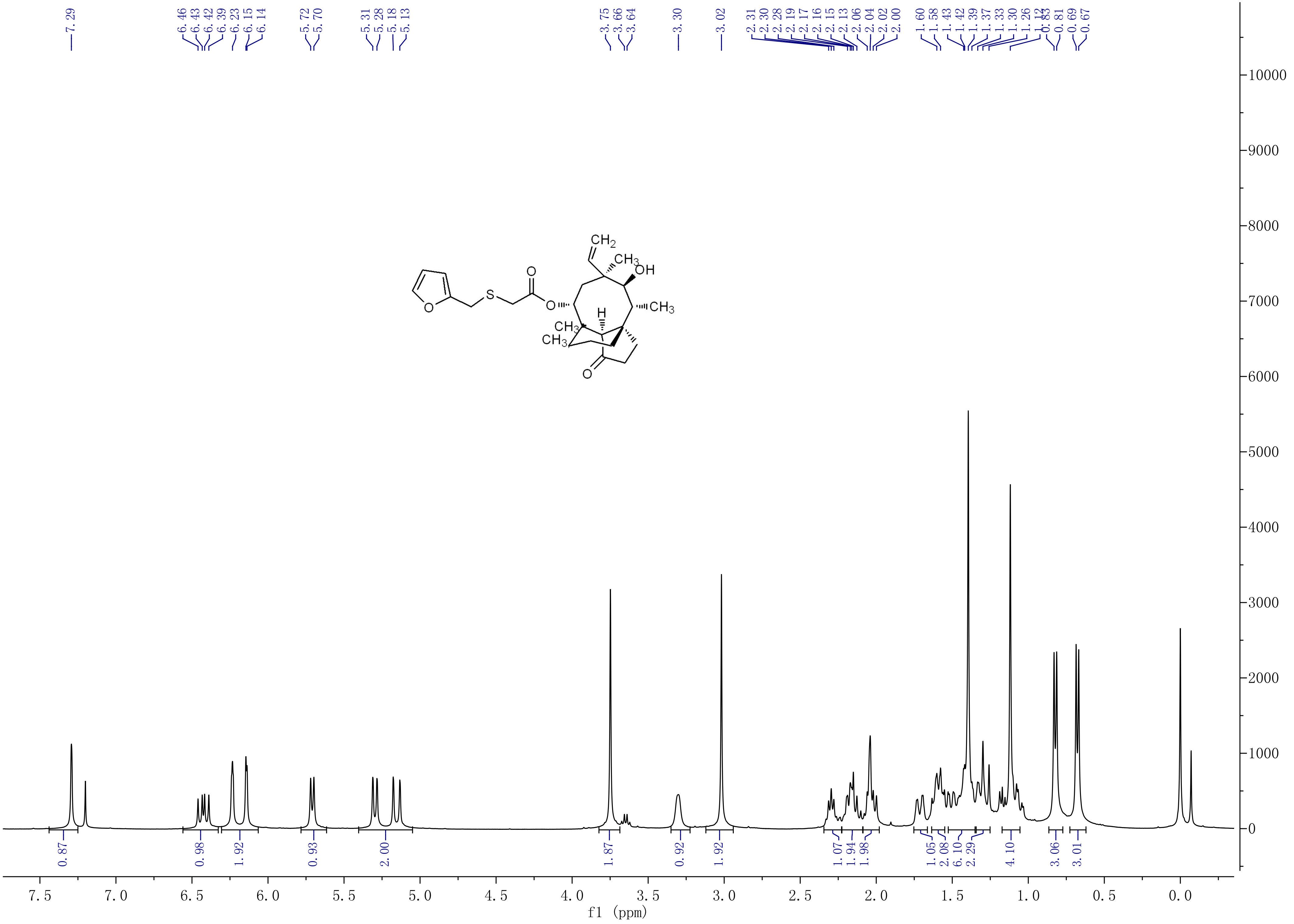

7.29
6.46 6.43 6.42 6.39 6.23 6.15 6.14
5.72 5.70
5.31 5.28 5.18 5.13
3.75 3.66 3.64
3.30
3.02
2.31 2.30 2.28 2.19 2.17 2.16 2.15 2.13 2.06 2.04 2.02 2.00
1.60 1.58 1.43 1.42 1.39 1.37 1.33 1.30 1.26 1.12 0.83 0.81 0.69 0.67
0.87 0.98 1.92 0.93 2.00 1.87 0.92 1.92 1.07 1.94 1.98 1.05 2.08 6.10 2.29 4.10 3.06 3.01
f1 (ppm)

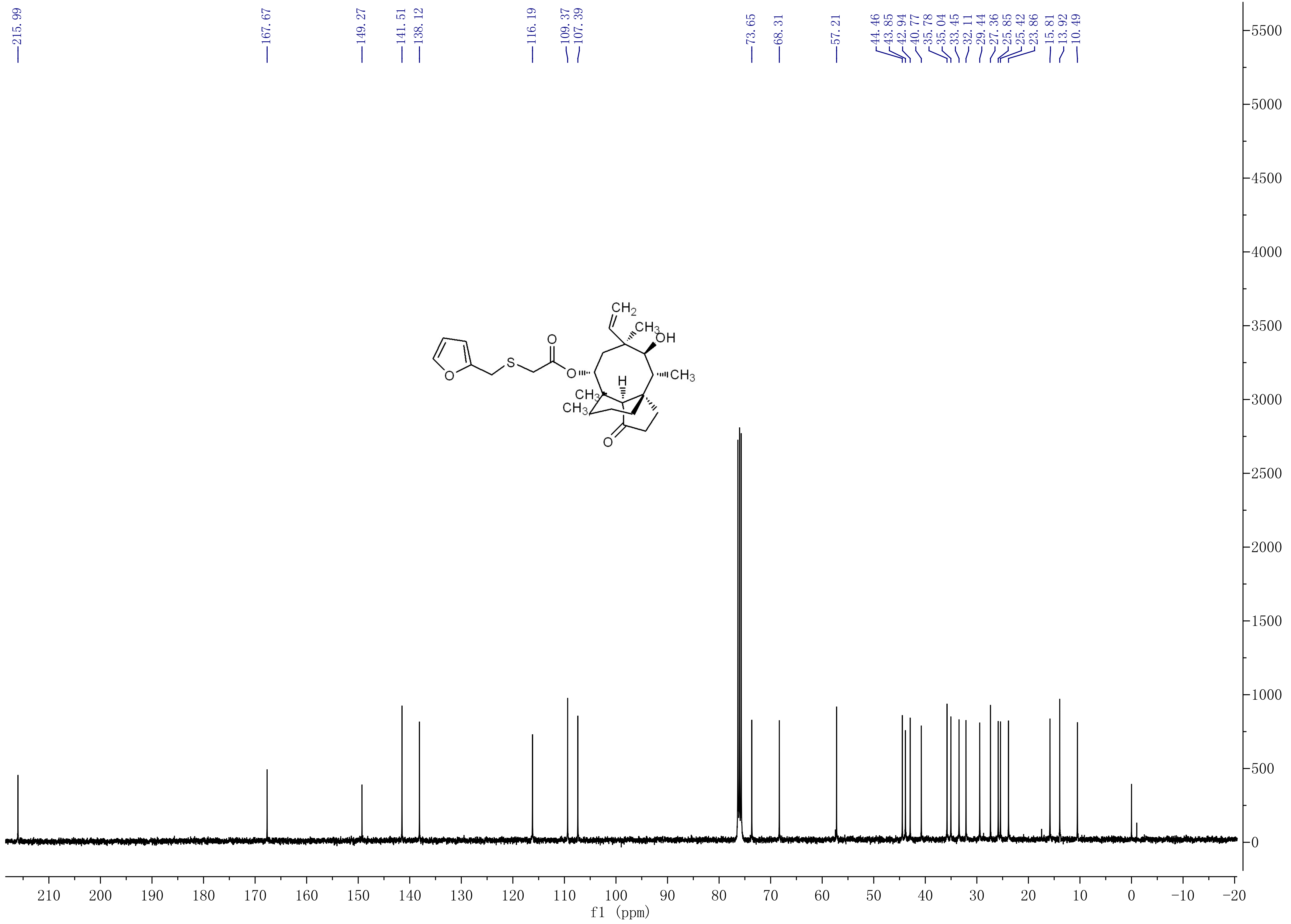
215.99
167.67
149.27
141.51
138.12
116.19
109.37
107.39
73.65
68.31
57.21
44.46
43.85
42.94
40.77
35.78
35.04
33.45
32.11
29.44
27.36
25.85
25.42
23.86
15.81
13.92
10.49
CH2
CH3
OH
O
S
O
O
CH3
H
CH3
CH3
O
210 200 190 180 170 160 150 140 130 120 110 100 90 80 70 60 50 40 30 20 10 0 -10 -20
f1 (ppm)
5500 5000 4500 4000 3500 3000 2500 2000 1500 1000 500 0

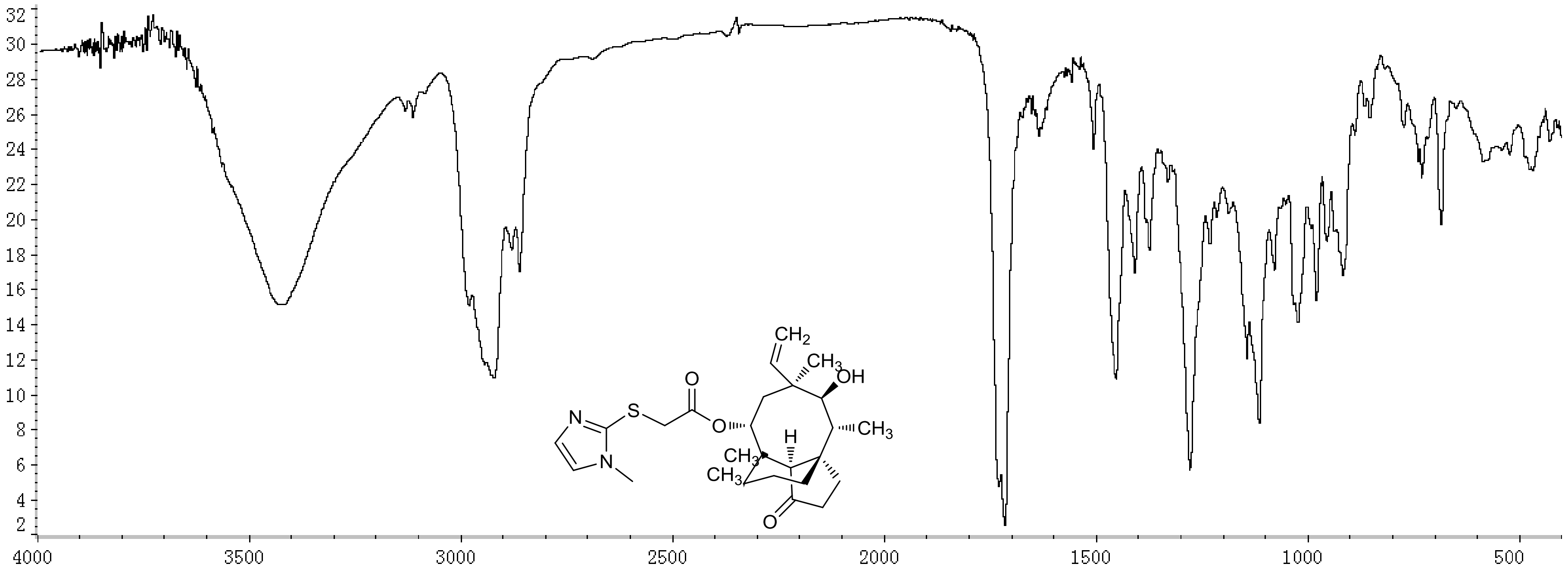
32
30
28
26
24
22
20
18
16
14
12
10
8
6
4
2
4000
3500
3000
2500
2000
1500
1000
500
N
S
O
O
N
CH2
CH3
OH
CH3
H
CH3
CH3
O

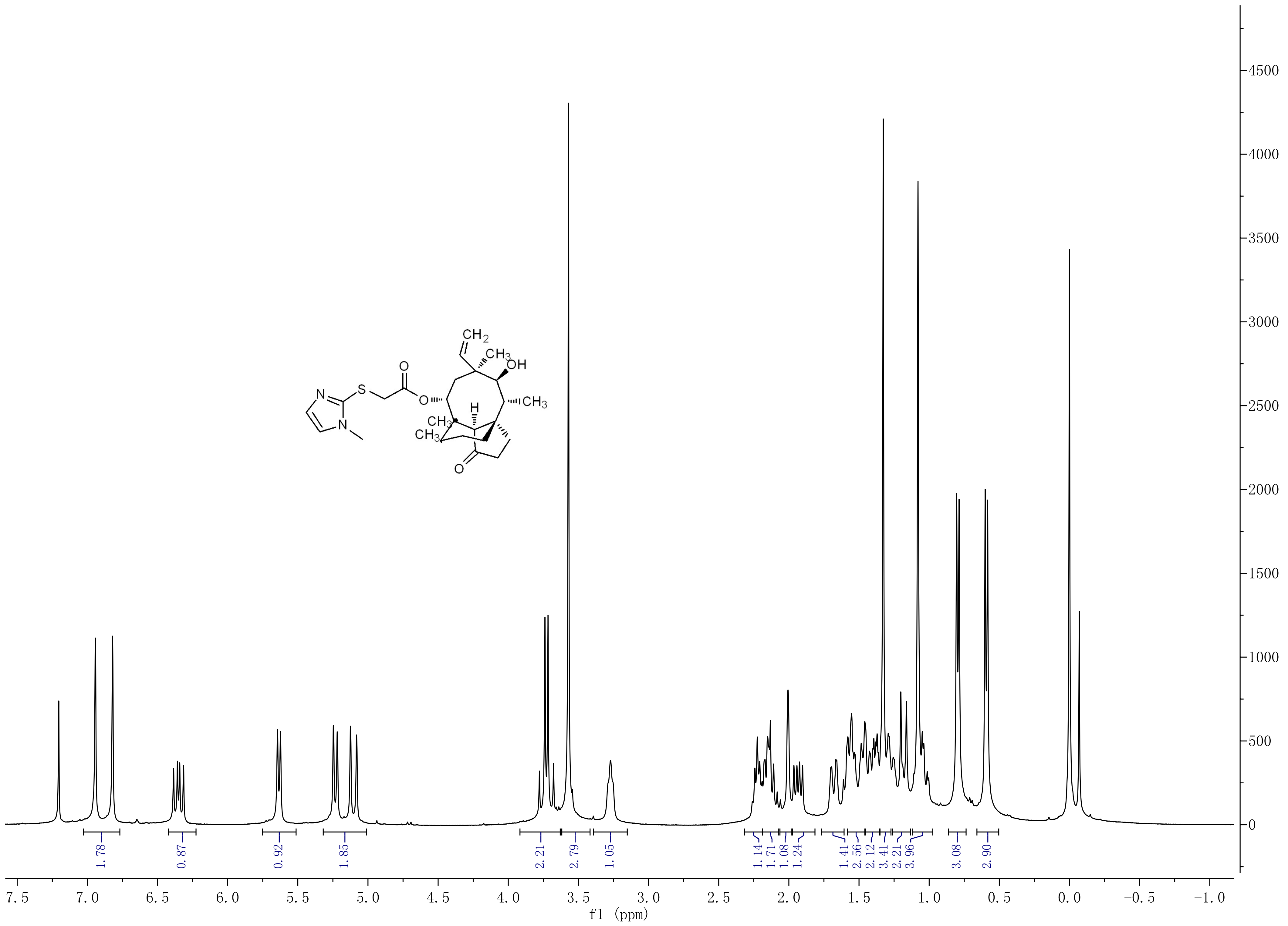

CH2
CH3
OH
O
N
S
O
CH3
H
CH3
CH3
N
O
1.78
0.87
0.92
1.85
2.21
2.79
1.05
1.14
1.71
1.08
1.24
1.41
2.56
2.12
3.41
2.21
3.96
3.08
2.90
7.5
7.0
6.5
6.0
5.5
5.0
4.5
4.0
3.5
3.0
2.5
2.0
1.5
1.0
0.5
0.0
-0.5
-1.0
f1 (ppm)
4500
4000
3500
3000
2500
2000
1500
1000
500
0

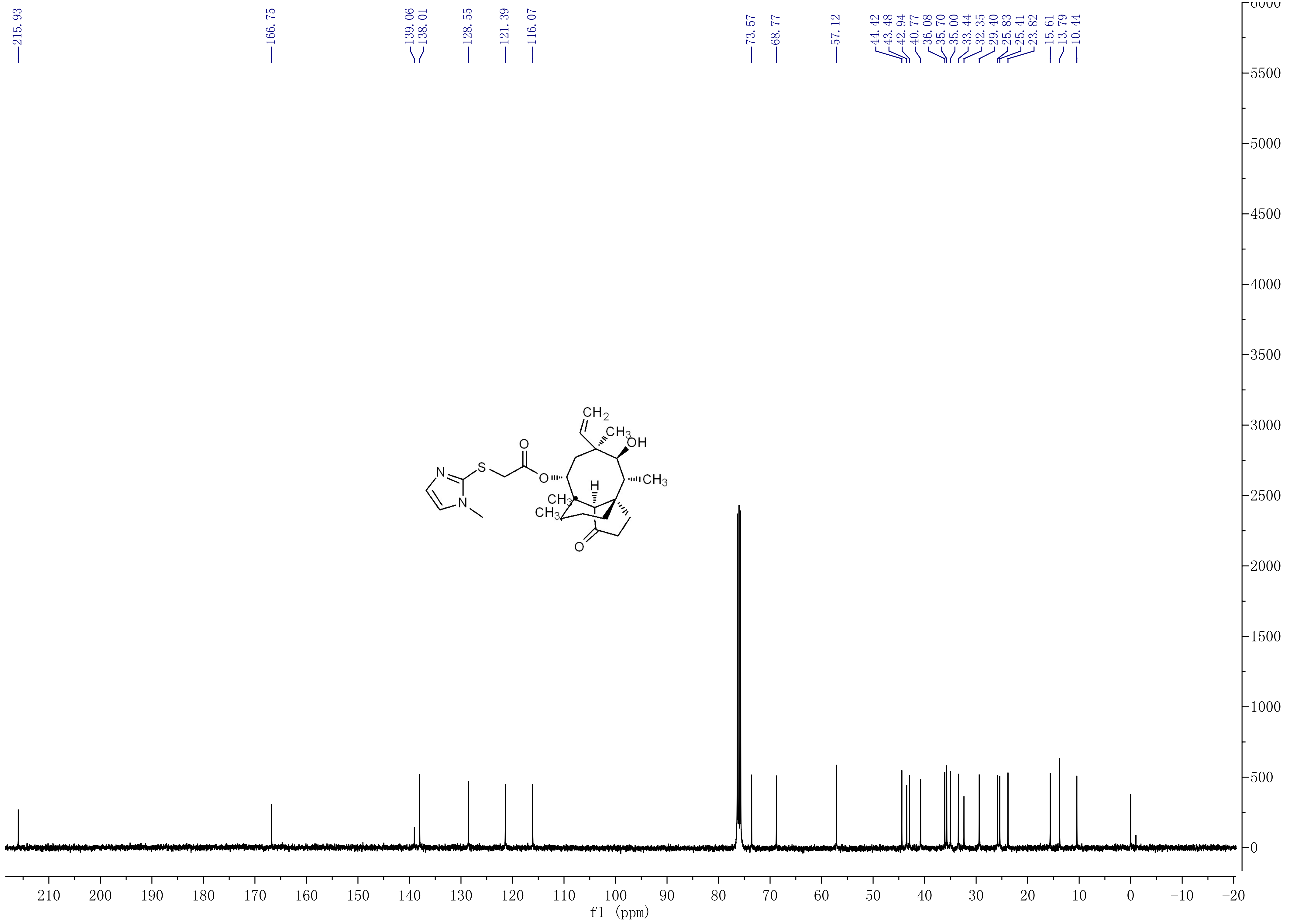

215.93
166.75
139.06
138.01
128.55
121.39
116.07
73.57
68.77
57.12
44.42
43.48
42.94
40.77
36.08
35.70
35.00
33.44
32.35
29.40
25.83
25.41
23.82
15.61
13.79
10.44
CH2
CH3
OH
O
S
N
N
O
CH3
H
CH3
CH3
O
f1 (ppm)

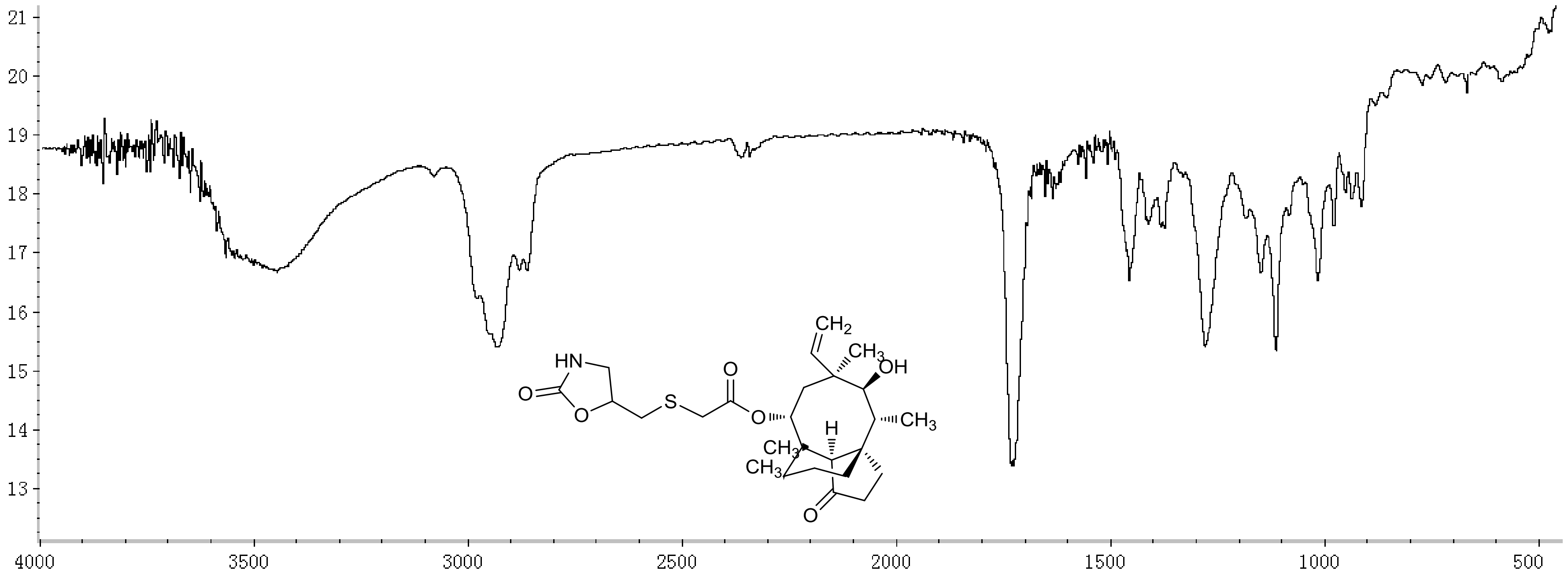
21
20
19
18
17
16
15
14
13
4000
3500
3000
2500
2000
1500
1000
500
HN
O
O
S
O
O
CH2
CH3
OH
CH3
H
CH3
CH3
O

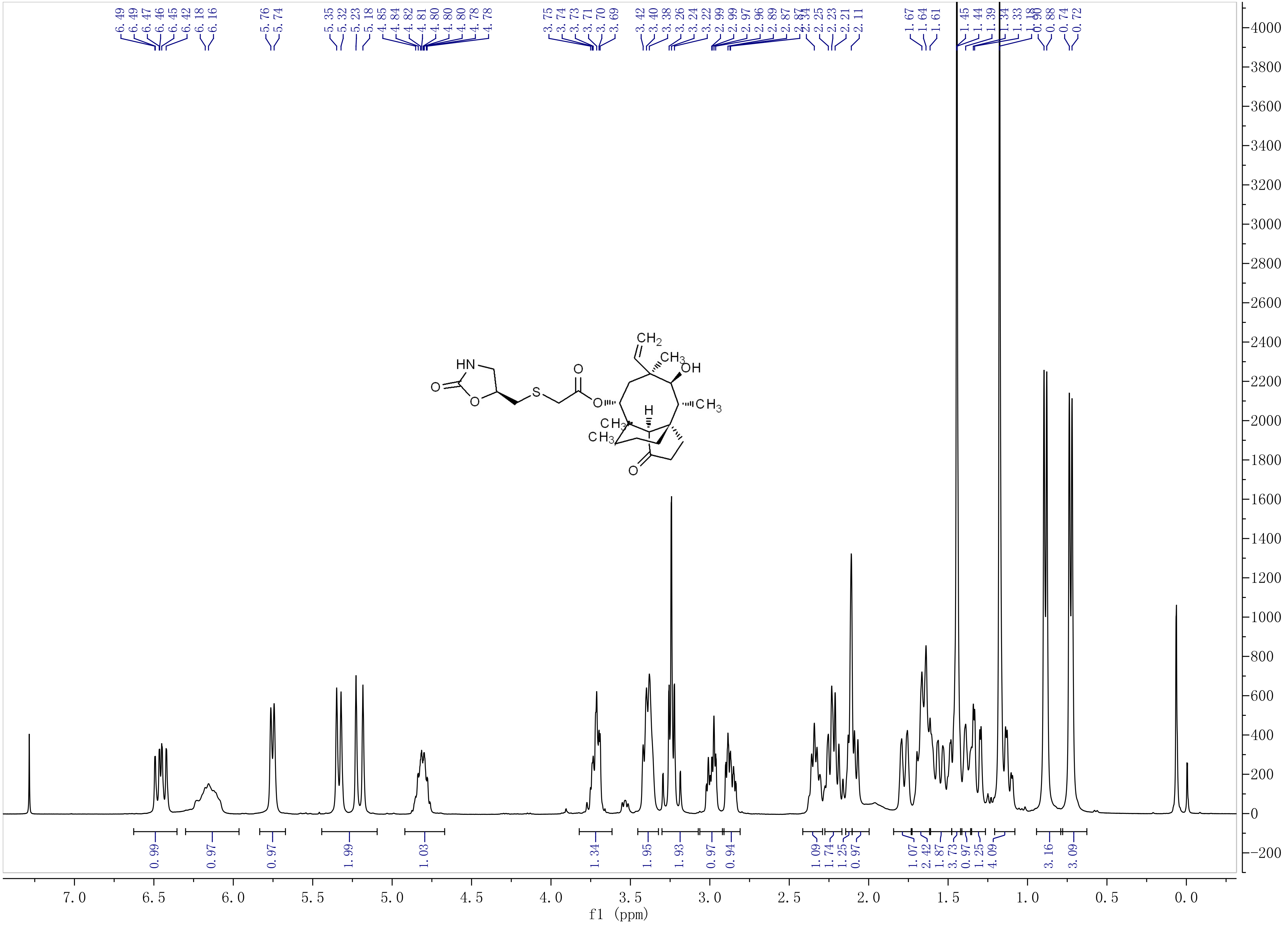
6.49 6.49 6.47 6.46 6.45 6.42 6.18 6.16
5.76 5.74
5.35 5.32 5.23 5.18 4.85 4.84 4.82 4.81 4.80 4.80 4.78 4.78
3.75 3.74 3.73 3.71 3.70 3.69
3.42 3.40 3.38 3.26 3.24 3.22 2.99 2.99 2.97 2.96 2.89 2.87 2.87 2.34 2.25 2.23 2.21 2.11
1.67 1.64 1.61
1.45 1.44 1.39 1.34 1.33 1.18 0.90 0.88 0.74 0.72
HN
O
O
S
O
O
CH2
CH3
OH
CH3
H
CH3
CH3
O
0.99
0.97
0.97
1.99
1.03
1.34
1.95
1.93
0.97
0.94
1.09
1.74
1.25
0.97
1.07
2.42
1.87
3.73
0.97
1.25
4.09
3.16
3.09
f1 (ppm)

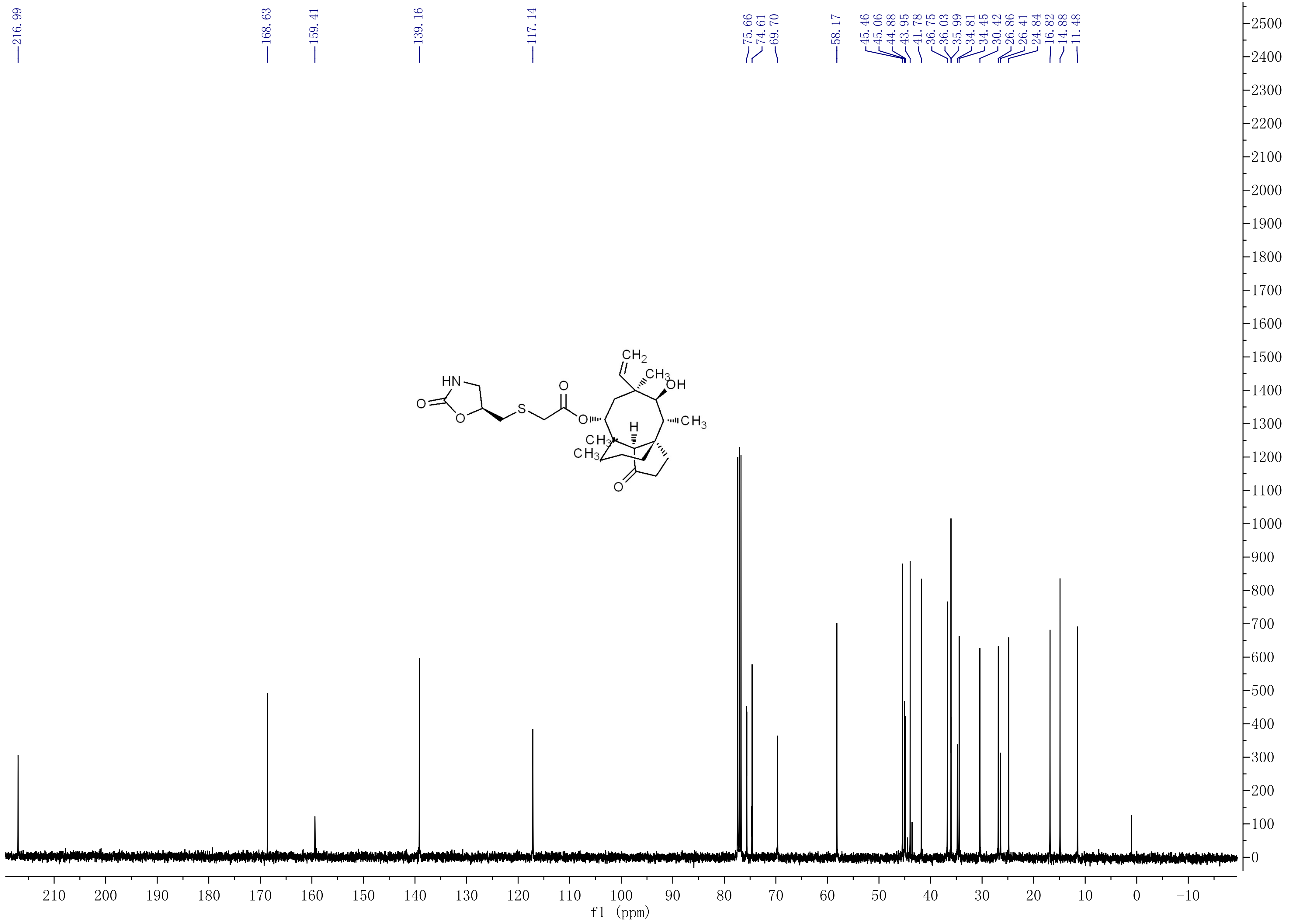
216.99
168.63
159.41
139.16
117.14
75.66
74.61
69.70
58.17
45.46
45.06
44.88
43.95
41.78
36.75
36.03
35.99
34.81
34.45
30.42
26.86
26.41
24.84
16.82
14.88
11.48
HN
O
O
S
O
O
CH2
CH3
OH
CH3
H
CH3
CH3
O
210 200 190 180 170 160 150 140 130 120 110 100 90 80 70 60 50 40 30 20 10 0 −10
f1 (ppm)
2500 2400 2300 2200 2100 2000 1900 1800 1700 1600 1500 1400 1300 1200 1100 1000 900 800 700 600 500 400 300 200 100 0
